# Differential Metabolic Dysregulations in Hepatocellular Carcinoma and Cirrhosis: Insights into Lipidomic Signatures

**DOI:** 10.3390/biom15111575

**Published:** 2025-11-10

**Authors:** Cristina-Paula Ursu, Luminița Elena Furcea, Bogdan Procopeț, Răzvan Alexandru Ciocan, Ștefan Ursu, Claudia Diana Gherman, Dan Vălean, Rodica Sorina Pop, Emil Ioan Moiș, Horia Ștefănescu, Carmen Socaciu, Nadim Al Hajjar, Florin Graur

**Affiliations:** 1 3rd Surgery Department, “Iuliu Hațieganu” University of Medicine and Pharmacy, Croitorilor Street, No. 19-21, 400162 Cluj-Napoca, Romania; pop_cristina_paula@elearn.umfcluj.ro (C.-P.U.); ursu_stefan@elearn.umfcluj.ro (Ș.U.); drmoisemil@elearn.umfcluj.ro (E.I.M.); florin.graur@umfcluj.ro (F.G.); 2 “Prof. Dr. Octavian Fodor” Regional Institute of Gastroenterology and Hepatology, Croitorilor Street, No. 19-21, 400162 Cluj-Napoca, Romania; 3 3rd Medical Department, “Iuliu Hațieganu” University of Medicine and Pharmacy, Croitorilor Street, No. 19-21, 400162 Cluj-Napoca, Romania; 4 Department of Surgery-Practical Skills, “Iuliu Hațieganu” University of Medicine and Pharmacy, Marinescu Street No. 23, 400337 Cluj-Napoca, Romania; 5 Department of Community Medicine, “Iuliu Hațieganu” University of Medicine and Pharmacy, Avram Iancu Street, No. 31, 400347 Cluj-Napoca, Romania; drsorinapop@yahoo.com; 6 Center for Applied Biotechnology Biodiatech, SC Proplanta, 400478 Cluj-Napoca, Romania

**Keywords:** hepatocellular carcinoma, cirrhosis, lipidomics, biomarkers, sphingolipids, glycerophospholipids

## Abstract

Hepatocellular carcinoma (HCC), the most common primary liver malignancy, usually develops in patients with cirrhosis, yet the metabolic mechanisms that distinguish the two conditions remain poorly understood. This study aimed to explore metabolic dysregulations in HCC compared with cirrhosis and to identify potential biomarkers, especially lipids, with diagnostic and prognostic value. We prospectively studied 81 patients—41 with HCC and 40 with cirrhosis—using high-resolution UHPLC-QTOF-ESI^+^-MS to characterize their serum lipidome. Across both groups, 322 metabolites were identified, but their distribution was strikingly different. Patients with HCC showed higher levels of sphingolipids, glycerophospholipids, diglycerides, sterols, and certain fatty acids, reflecting tumor-related metabolic rewiring. In contrast, cirrhotic patients had increased D-glucose, 5-hydroxymethyluracil, lysophospholipids, acylcarnitines, and specific fatty acid derivatives. Several lipids, such as CerPE(d16:2/24:1(2OH)), SM(d18:0/14:0), PA(36:6), and GlcCer(d18:1/12:0), displayed excellent discriminative accuracy, highlighting their role as putative biomarkers. These findings underscore the importance of lipid metabolic reprogramming in HCC, characterized by membrane remodeling, energy adaptation, and oxidative stress resistance. Integrating lipidomic profiling into clinical practice could improve early detection and risk stratification in cirrhotic patients. Larger, multicenter studies are needed to validate these biomarkers and assess their therapeutic implications.

## 1. Introduction

Hepatocellular carcinoma (HCC) is the leading form of primary liver malignancy and remains a significant global health issue, as liver cancer is the second most common cause of malignant-related mortality worldwide [1,2,3]. A significant percentage of cases occur in individuals with chronic liver illness, particularly associated with hepatitis B virus (HBV) or hepatitis C virus (HCV) infections, as well as those with a history of chronic alcohol intake. Furthermore, the prevalence of non-viral hepatocellular carcinoma is rising due to a significant increase in obesity, diabetes, and alcohol consumption among young individuals [1,4,5]. Nonalcoholic fatty liver disease (NAFLD) instances are projected to rise by 21% by 2030, while nonalcoholic steatohepatitis (NASH) cases will go up by 63%. The overall prevalence of NAFLD among adults (aged ≥ 15 years) is anticipated to reach 33.5% by 2030. Consequently, there will be a corresponding rise in decompensated cirrhosis, with HCC anticipated to escalate by 137% [6,7,8].

The worldwide incidence of HCC is increasing, requiring enhanced methods for detection, diagnosis, and ongoing surveillance of the illness [1,2]. Patients with HCC are typically identified at an intermediate or advanced stage. A suitable treatment method is generally determined by taking into consideration tumor stage, liver function, and performance status, in accordance with the new Barcelona Clinic Liver Cancer (BCLC) staging system. Surgical excision yields optimal outcomes regarding overall and disease-free survival for patients with very early or early hepatocellular carcinoma and intact liver function [9,10,11,12,13,14]. Therefore, there is a need for more accurate disease indicators, especially for the early identification of HCC, with specific and sensitive biomarkers serving as stronger primary indicators. As previously stated, liver cirrhosis is a significant risk factor for the development of HCC (80–90% of liver cirrhosis (LC) patients). The predominant diagnostic methods employed to survey these patients are liver ultrasonography and alpha-fetoprotein (AFP). The limited sensitivity and specificity of the aforementioned markers demand further research into novel biomarkers [10,15,16,17,18,19].

Recent breakthroughs in oncology have focused on the emerging field of metabolomics, specifically focusing on the crucial role of lipids in hepatocellular carcinoma, since the liver contains a plethora of lipids and other metabolites that are perpetually undergoing various biochemical transformations. The alteration of lipid metabolism is a key factor in carcinogenesis, with an extensive variety of lipid metabolites exhibiting complex cancer-promoting effects that have yet to be fully clarified [17,18]. Moreover, metabolic reprogramming refers to a series of changes that occur during carcinogenesis and produce metabolites which enhance the survival of cancer cells. The two most well researched pathways of such metabolic reprogramming are the Warburg effect and glutaminolysis. Modifying cellular energy processes is a crucial survival tactic of cancer cells, reflected by the promotion of aerobic glycolysis and lactic fermentation over mitochondrial oxidative phosphorylation, which explains the Warburg effect. The high “starvation” of lipids in neoplastic tissues is compensated by external uptake and de novo lipogenesis; consequently, fatty acid oxidation is also increased in various tumor forms [17,20,21,22].

HCC is generally marked by the upregulation of genes associated with fatty acid synthesis, including ATP-citrate lyase (ACLY), acetyl-CoA carboxylase (ACC), and fatty acid synthase (FASN), which facilitate the conversion of citrate to acetyl-CoA, malonyl-CoA, and fatty acids, respectively. Fatty acids are transformed into monounsaturated fatty acids (MUFA), which serve as precursors for the formation of triacylglycerols (TAG) [18,22,23,24,25]. Additional studies corroborate the findings of elevated saturated and monounsaturated fatty acids, while polyunsaturated fatty acids diminish in HCC. Nonetheless, the pathogenesis of HCC is intricate, including multiple variables including cytokine profiles, endoplasmic reticulum stress, insulin resistance, oxidative stress, gut microbiota, and genetic predispositions. Furthermore, lipid alterations become fundamental in the genesis and adaptation of HCC [17,18,23]. Research indicated that metabolites associated with lipid and amino acid metabolism can distinguish hepatocellular carcinoma patients from those with cirrhosis, and can forecast disease progression [26,27,28,29].

UHPLC-QTOF-ESI^+^-MS technology offers significant advantages for the metabolic fingerprinting and profiling of blood from such patients, including high sensitivity for detecting analytes, a high-resolution separation, and accurate mass measurements for confident compound identification. Furthermore, it can provide comprehensive and detailed analysis characterization of lipids or other metabolites of interest in biofluids, to be finally proposed as diagnosis and prognosis biomarkers [30,31].

The present study aims to compare the metabolic dysregulations between patients with cirrhosis and HCC, focusing primarily on specific lipidomic signatures along with other metabolites identified under similar analytical conditions, to uncover potential biomarkers for disease diagnosis and progression.

## 2. Materials and Methods

### 2.1. Study Population

The present paper was realized by conducting an analysis of a prospectively collected database. The patients from which we collected data and samples were admitted at the Surgical and Gastroenterology and Hepatology Departments of the “Prof. Dr. Octavian Fodor” Regional Institute of Gastroenterology and Hepatology, Cluj-Napoca, Romania, between March 2023 and June 2024. The patients admitted at the Surgical Department were 41 individuals diagnosed with HCC (BCLC 0, A and B), waiting for surgical or ablative treatment with curative intent, while the patients admitted at the Gastroenterology and Hepatology Department were 40 patients with liver cirrhosis (C–P classification, A and B). Patients with HCC were diagnosed in accordance with the EASL guidelines via imaging and/or histology, while staging was conducted based on the BCLC score. Patients with cirrhosis, serving as the control group, were diagnosed by non-invasive techniques including abdominal ultrasonography, transient elastography, serology, or liver biopsy. Additionally, the control group displayed multiple etiologies for liver cirrhosis, including hepatitis B or C virus, persistent alcohol intake, and non-alcoholic steatohepatitis. All participants chosen for this study were above 18 years of age. All patients were classified as Child–Pugh class A or B and patients with HCC were assessed upon admission using the Eastern Cooperative Oncology Group (ECOG) Performance Status Scale, which uses a grading system from 0 to 5 based on the patient’s functional capacity regarding self-care, daily activities, and physical abilities such as walking and working [32,33].

A comprehensive analysis was conducted on both digital and paper written medical records of individuals who were diagnosed with HCC and cirrhosis, following certain inclusion and exclusion criteria. All of the information needed to complete the study was collected in excel forms and re-evaluated prior to statistical analysis. Demographic information (e.g., gender, age, environment) was collected. Clinical information and paraclinical examinations (e.g., the presence of hepatitis B or C virus infection, chronic alcohol intake, steatohepatitis, chronic liver disease, total and conjugated bilirubin, albumin, total proteins, thrombocytes, INR, AFP, GOT (glutamic-oxaloacetic transaminase), GPT (glutamic-pyruvic transaminase), ascites, and encephalopathy) were evaluated. TE-LSM and HVPG were assessed, and Child–Pugh (C–P) classification and BCLC staging were established at admission prior to therapy allocation. Moreover, a TAC score was calculated for each individual [34,35].

Operative details, including the type of surgical procedure, the duration of intervention, intraoperative blood loss, and the histopathological characteristics of the respective specimen (e.g., number and dimension of nodules) were introduced. Further, postoperative data regarding follow-up (postoperative liver decompensation and survival) were obtained. Patients with advanced hepatocellular carcinoma who did not necessitate curative intervention, those with concurrent malignancies other than HCC, individuals with compromised performance status, or those with health conditions (acute inflammatory diseases, significant coagulation disorders, poorly controlled diabetes mellitus, NYHA IV cardiac insufficiency, KDOQI stage 4/5 chronic renal disease, immunosuppressed individuals, or those currently undergoing steroid therapy) that precluded surgical or ablative treatment were excluded from the study.

The cohort was investigated prospectively, and serum lipid parameters were evaluated in previously stored serum samples at −80 °C.

### 2.2. Patients’ Follow-Up

Patients were followed up 3 and 6 months after the surgical treatment to assess the recurrence of HCC. Additionally, data concerning postoperative liver decompensation and survival were acquired. Postoperative decompensation was characterized by the occurrence of any of the following during patient follow-up: liver failure, jaundice (defined as hyperbilirubinemia >3 mg/dL), hepatic encephalopathy, ascites, post-hepatectomy hemorrhage, or acute kidney injury. Mortality was defined as the incidence of death until the end of the study timeline.

Patients with advanced hepatocellular carcinoma who did not necessitate curative intervention, those with concurrent malignancies other than HCC, individuals with compromised performance status, or those with health conditions (acute inflammatory diseases, significant coagulation disorders, poorly controlled diabetes mellitus, NYHA IV cardiac insufficiency, KDOQI stage 4/5 chronic renal disease, immunosuppressed individuals, or those currently undergoing steroid therapy) that precluded surgical or ablative treatment were excluded from the study.

### 2.3. Lipidomic Analysis

#### 2.3.1. Sample Preparation and Processing

Blood samples were collected by venipuncture in sterile Kima vacutainers with silicium as a coagulant factor, from both patient categories after admission and prior to surgical intervention for individuals with HCC. Each container was maintained at room temperature for thirty minutes and subsequently subjected to centrifugation, and the resultant serum was preserved at −80 °C. They were assigned confidential numerical identifiers. To 0.2 mL of blood serum, 0.8 mL of a solvent mixture (Methanol–Acetonitrile–Methyl Tert-Butyl Ether at a ratio of 1:1:0.25) was added. All reagents were of high purity (UHPLC grade) and were supplied by Merck (Merck GmbH, Darmstadt, Germany). The mixture was vortexed for 30 s and maintained at −20 °C for 24 h to facilitate protein precipitation. Following defrosting, the vials underwent centrifugation at 12,500 rpm for 10 min, after which the supernatant was collected, filtered through 0.2 µm PTFE filters (Millipore, Burlington, VT, USA), and transferred into autosampler vials for metabolomic analysis. Fifteen microliters of Internal Standard (2 mg/mL Doxorubicin hydrochloride) were added to 0.5 mL of supernatant.

#### 2.3.2. UHPLC-QTOF-ESI^+^-MS Analysis

The UHPLC-MS analysis was performed on a Bruker Daltonics MaXis Impact (Bruker GmbH, Bremen, Germany) device including a Thermo Scientific HPLC UltiMate 3000 system with a Dionex Ultimate quaternary pump delivery (Waltham, MA, USA) and ESI^+^-QTOF-MS detection, on a C18 reverse-phase column (Kinetex, Phenomenex, Torrance, CA, USA) (UPLC C18) (5 µm, 4.6 × 150 mm) at 25 °C and at a flow rate of 0.8 mL/min. The injection volume was 25 microliters. The mobile phase was represented by a gradient of an eluent A (pure water containing 0.1% formic acid) and eluent B (Methanol–Acetonitrile–Isopropanol, 1:1:1, containing 0.1% formic acid). The gradient system consisted of 70% A (min 0), 30% A (min 4), 0% A (min 7), 30% A (min 10), and 70% A (min 13), followed by 2 min isocratic elution with 70% A. The total running time was 15 min. Parallel quality control samples were generated by combining 0.2 mL of serum from each sample within each group. To ensure excellent data repeatability, the QC samples were run again, after every ten samples. Each sample was analyzed in duplicate.

**Figure 1:**
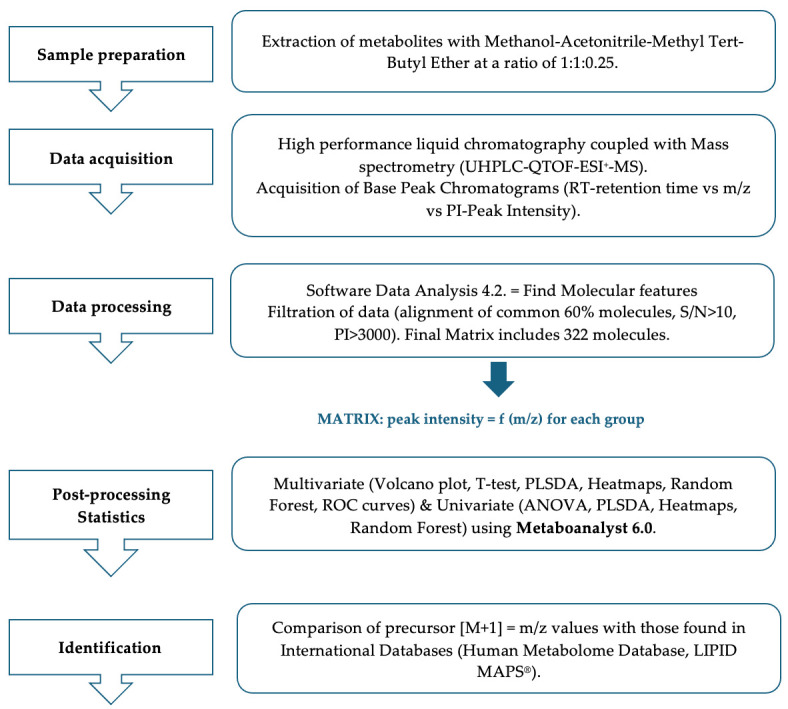
The methodology steps applied in this study.

The MS parameters were set for a mass range between 100 and 1000 Da. The nebulizing gas pressure was set at 2.8 bar, the drying gas flow at 12 L/min, and the drying gas temperature at 300 °C. During each chromatographic run, a calibration with Sodium formate was performed. The control of the instrument and data processing used the specific software provided by Bruker Daltonics, namely Chromeleon (7.2 version), TofControl 3.2, Hystar 3.2, and Data Analysis 4.2. The steps of the whole methodology applied in this case are presented in Figure 1.

#### 2.3.3. Data Processing and Statistical Analysis

The baseline clinical and paraclinical characteristics of our cohort were obtained by statistical analysis using SPSS (IBM, version 26), considering the significance value at *p* < 0.05.

The data were processed using Data Analysis 4.2. First, the individual Total Ion Chromatograms (TICs) were registered, then transformed to Base Peak Chromatograms (BPCs), and the compound spectra were recorded using the FMF (Find Molecular Features) function. The FMF matrix contained the retention time, the peak areas and intensities, and the signal/noise (S/N) ratio for each separated molecule together with its *m*/*z* (mass-to-charge ratio) value representing the precursor [M+1] value. In BPCs, the number of separated compounds ranged between 1200 and 1800 molecules. The molecules with a retention time lower than 1.6 (void volume of the LC column) and peak intensities lower than 3000 were eliminated, and the ones with S/N values below 10 were skipped. The *m*/*z* values of 60% common molecules found in samples were aligned using the online software (computational tool) from https://www.bioinformatics.org/bioinfo-af-cnr/NEAPOLIS/ (accessed on 30 August 2024) and the final matrix including 322 molecules (*m*/*z* values) for each sample was submitted to statistical analysis as a .csv file and processed by the online software Metaboanalyst 6.0 (www.metaboanalyst.ca). Different algorithms for multivariate analysis and biomarker analysis were applied, to collect complementary information, as presented in Table 1. The experimental precursor *m*/*z* values (adduct [M+H^+^]) were compared with the average of theoretical isotopic mass and a mass tolerance of 0.05 Da, according to International Databases, the Human Metabolome Database (https://hmdb.ca/), and LIPID MAPS^®^ Lipidomics Gateway (https://www.lipidmaps.org (accessed on 3 September 2024).

### 2.4. Ethical Approval

The study was approved by the ethical department of the “Prof. Dr. Octavian Fodor” Regional Institute of Gastroenterology and Hepatology, Cluj-Napoca, Romania (15602/22 December 2022) and the “Iuliu Hatieganu” University of Medicine and Pharmacy, Cluj-Napoca, Romania (No. 24/13 February 2023). In each situation, informed consent regarding data and sample collection was obtained in accordance with the principles outlined in the Declaration of Helsinki.

## 3. Results

### 3.1. Descriptive Characteristics of Enrolled Patients

The baseline clinical and paraclinical characteristics of the cohort are included in Table 2.

The patients enrolled in our research had a mean age of 66 years old in the HCC group and 60 years old in the cirrhotic patients’ group, with most of the patients belonging to the male sex. In this study, 43.9% of patients with HCC had overweight Body Mass Index (BMI) values, 14.6% displayed normal weight, and 41.4% were classified as obese. Most LC patients were classified as overweight (65%), whereas 27.5% were of normal weight. Considering the ECOG Scale, one patient out of 41 with HCC had a performance status (PS) of 2, while the rest were classified with a PS of 0 (3 patients) or 1 (37 patients), indicating that they either have the same physical abilities as before or have limitations in physically demanding activities but maintain mobility, and are therefore capable of performing light or sedentary tasks, such as minor housekeeping or office work.

Despite efforts to match patients in both categories regarding the etiology of liver disease, we observed a predominance of HCV patients in the HCC cohort, while most cirrhotic patients had a history of chronic alcohol consumption. The paraclinical examinations showed a total bilirubin mean value higher in the cirrhotic patients’ group. Significant changes were noted between the two groups concerning serum albumin levels and thrombocytes number. In liver cirrhosis, albumin levels and the count of platelets may be diminished due to the liver’s impaired ability to synthesize albumin, together with the presence of hypersplenism, decreased thrombopoietin synthesis, and bone marrow suppression regarding platelets. The liver is accountable for numerous metabolic activities related to lipids and lipoproteins; hence, serum lipid levels may decrease in liver cirrhosis. The basis for these modifications arises from the observation that, while hepatocellular carcinoma usually appears in cirrhotic livers, certain patients do not have underlying cirrhosis.

In the current study, 34.14% of patients had HCC without cirrhosis, while 21 patients had cirrhosis classified as Child–Pugh Class A. Although most individuals were already patients with liver cirrhosis, they were in a compensated state of the disease.

Regarding the following parameters, there was a statistically significant comparison between the two sets: ascites (14.6% HCC patients had ascites vs. 45% of cirrhotic patients; *p* = 0.001), Child–Pugh classification (21 HCC patients were classified as C–P A, 6 as C–P B and 14 had no cirrhosis; *p* = 0.001), HVPG (*p* = 0.02), TE-LSM (*p* = 0.001), and MELD score (87.5% of patients with liver cirrhosis had a score between 10 and 19; *p* = 0.001).

Patients admitted to the Surgical Department typically exhibited a range of complex oncological situations that necessitated specialized preoperative care. Each individual had a comprehensive investigation, and blood analysis was assessed immediately upon admission and prior to any medication delivery. Patients typically received hydroelectrolytic and acid-base support as necessary to preserve the body’s fluid and electrolyte equilibrium. We considered a variety of preventive measures: preoperative antimicrobial prophylaxis and thromboprophylaxis. Patients may required protein or albumin supplementation, hepatic or nutritional assistance, or medication for pain relief. Furthermore, patients with liver cirrhosis, after a thorough evaluation, received supportive medication and fluids customized to their clinical and paraclinical needs, without being scheduled for surgery, but rather for particular treatments. Certain patients with HCV underwent treatment with Sofosbuvir/Ledipasvir, whilst certain patients with HBV received Lamivudine therapy. Patients with hepatocellular carcinoma and alcoholic cirrhosis were abstinent.

Amongst the 41 patients with hepatocellular carcinoma, 11 had a documented tumoral recurrence and 16 patients died before the end of the research time interval.

### 3.2. Untargeted Multivariate Analysis of CIR vs. HCC Group

#### 3.2.1. Volcano Plot and Correlation Plot (CIR vs. HCC)

**Figure 2:**
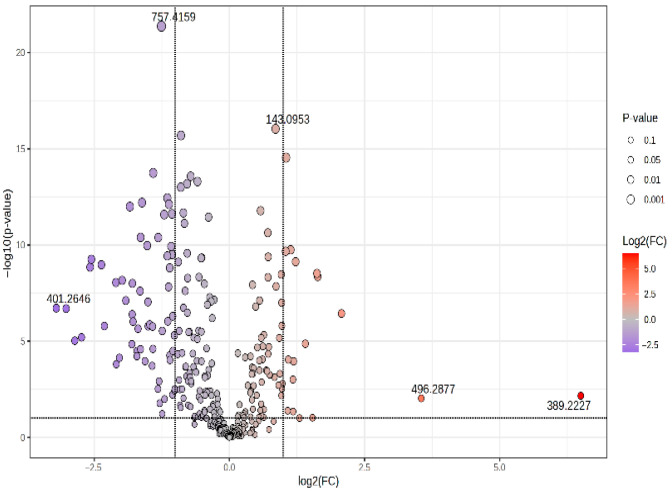
Volcano plot representing the *m*/*z* values of molecules with increased MS intensity levels in the HCC group (log2FC < 0) and decreased levels (log2FC > 0) comparative to the CIR group. The stratification of molecules was identified according to the correlation values (*T*-test, −log10 (FDR, *p*-value)).

The volcano plot (Figure 2) illustrates the results of a *T*-test by plotting the logarithm of −log10 (*p*-value) on the *y*-axis against the logarithm of the fold-change (log2FC) on the *x*-axis. The *m*/*z* values of compounds with log2FC > 0 indicate elevated levels in the CIR group and those with log2FC < 0 indicate decreased levels relative to the HCC group. Therefore, the upper-left section lists *m*/*z* values of molecules with elevated levels in the HCC group, while the upper-right portion lists *m*/*z* values of molecules with elevated levels in the CIR group. Two of the molecules with an increased level in the HCC group were CerPE(d16:2/24:1(2OH)) and 5,6-trans-25-Hydroxyvitamin D2, while the following components displayed a higher level in the cirrhotic patients’ group: 5-Hydroxymethyluracil, LysoPS (16:1), and N-Linoleoyl Taurine.

#### 3.2.2. Discriminatory Analysis by PLSDA

**Figure 3:**
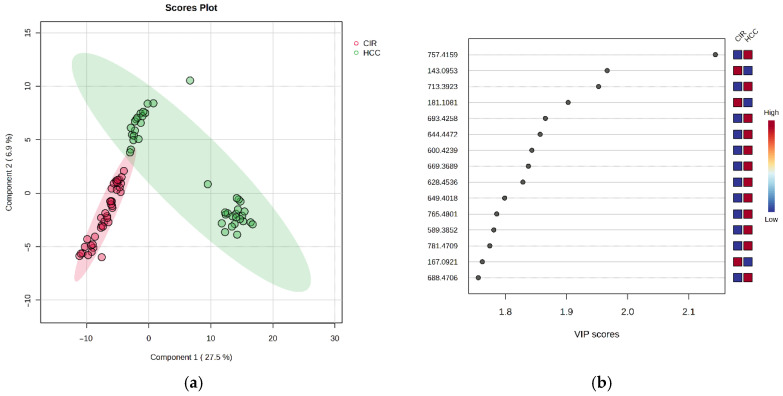
(**a**) Partial least squares discriminant analysis (PLSDA) plot showing the discrimination between the CIR and HCC groups. (**b**) PLSDA loadings plot, showing the VIP scores of the main 15 molecules selected as representative for the discrimination between the CIR and HCC groups.

The co-variance for the first five components was evaluated according to the supervised PLSDA (partial least squares discriminatory analysis) score plot. The explained co-variance of the CIR group vs. the HCC group was 34.4%, the discrimination being significant (Figure 3a). From the PLSDA loadings plot, the most relevant molecules which could be considered responsible for the discrimination were selected. Figure 3b represents the *m*/*z* values of these molecules and the VIP scores above 1, for the top 15 molecules, showing their relative variation (higher or smaller, colored in red and blue, respectively). The identification of these molecules is presented in Table S1 (attached as a Supplementary File). The CIR group was highly homogeneous, while the HCC group was less homogeneous, with an apparent split into two subgroups. The cross-validation algorithm when the first five components were considered indicated an accuracy over 0.9, a maximal value of R2 over 0.9, and Q2 values around 0.8, suggesting a good predictability for this model, which is significant when five components are considered.

#### 3.2.3. The Random Forest Analysis and Heatmap

**Figure 4:**
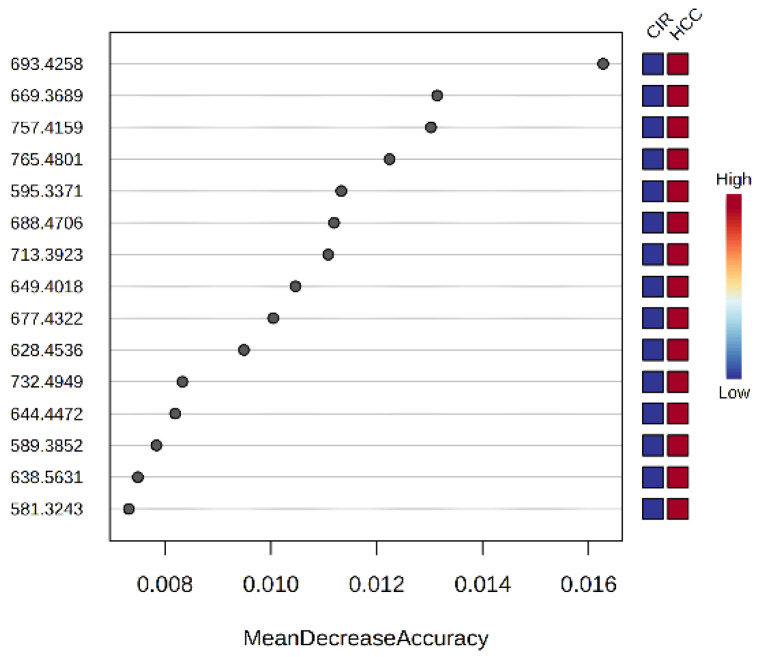
The MDA values vs. *m/z* of the top 15 molecules according to Random Forest (RF) analysis.

Figure 4 presents the Mean Decrease Accuracy values for the first 15 predictive molecules, according to Random Forest analysis.

Figure 4 presents the MDA (Mean Decrease Accuracy) values for the first 15 predictive molecules, according to Random Forest analysis. A higher MDA value indicates the importance of that metabolite in the predicting group. This analysis brings complementary information about the predictive molecules to be considered as putative biomarkers of differentiation between the CIR and HCC groups.

Some of the top 15 molecules with higher MDA values are identical with the ones mentioned in Figure 3b.

Table 3 integrates the complementary data obtained by different multivariate analysis, namely, the VIP scores, fold-change (FC), log2FC, *p*-values (as determined by *T*-test), and MDA scores from RF ranking of molecules which may discriminate the CIR and HCC groups. The relative variation is mentioned in each case.

The results show that mainly the polar molecules like D-Glucose, Methylxanthine, and 5-Hydroxymethyluracil showed lower levels in the HCC group. The rest of the molecules are lipids from different classes and showed higher levels in the HCC group, suggesting the upregulation of lipid metabolism in the HCC group compared to CIR. These results reflect the potential of different lipid classes to be considered biomarkers of differentiation between the CIR and HCC groups.

#### 3.2.4. Biomarker Analysis and Correlation Network

According to Metaboanalyst 6.0 software, the biomarker analysis includes the presentation of the receiver operating curve (ROC) and the values of the area under the ROC (AUC) as a useful tool to evaluate the accuracy, by representing the sensitivity versus specificity of each molecule which may be considered as relevant biomarker. Elevated AUC values approaching 1 for a certain compound indicate its enhanced predictive capability as a biomarker. Table 4 shows the identification of top 15 molecules, their decreasing AUC values, the *p*-values (cutoff of 0.001) and log2FC values for each molecule, as well its relative variation in the CIR vs. HCC group. The log2FC values obtained below signify an elevated (negative values) or diminished (positive values) concentration of the metabolite in the HCC compared to the CIR group. Significantly high AUC values above 0.950 showed that top 15 molecules might be considered as putative biomarkers of differentiation, mostly lipids with increased levels in the HCC group, belonging to sphingolipid and glycerophospholipid classes, to be considered as predictive molecules with good prognostic values for differentiation.

The DSPC Network, an algorithm included in the Metaboanalysys 6.0 was also applied and presents the links between the most representative metabolites in different lipid metabolic pathways, the nodes representing the input metabolites, while the edges represent the association measures (see Figure S1—attached as a Supplementary File). The metabolites identified are shown, as well the top correlations (edges) based on their *p*-value rankings (top 20%).

### 3.3. Semi-Targeted Metabolomic Analysis

The total number of 322 common molecules identified in the CIR and HCC groups were classified according to their molecular structure. Therefore, the metabolites were included in 11 classes of metabolites, as presented in Table S1.

#### 3.3.1. VIP Scores, FC Values, *p*-Values and Random Forest Ranking

Table S2 (attached as a Supplementary File) includes The VIP scores, fold-change (FC), log2(FC), *p*-values (as determined by volcano plot and *T*-test) and RF ranking of molecules from each of the 11 metabolite classes which may discriminate the CIR vs. HCC group. The relative variation is mentioned in each case. Figure S2 (attached as a Supplementary File) includes for each class of metabolites identified in the CIR and HCC groups, the PLSDA score plots, VIP scores, Cross Validation graph, volcano plot, Heatmap and Random Forest (Rf) plot.

We detected distinct concentrations of molecules in individuals with HCC and cirrhosis across each class of metabolites, indicating significant disparities between the two patient categories. The predominant free fatty acid molecules with elevated prevalence in HCC patients include Hydroxy-Eicosapentaenoic acid C20:5;0, Octatriacontanoic acid C38:0, Heptadecenoic acid C17:1, Arachidic acid C20:0, Stearic acid C18:0, and Tetracontahexaenoic acid C40:6, whereas Triacontatrienoic acid C30:0 was found to be elevated in cirrhotic patients. Concerning fatty acid derivatives, Linoleyl arachidate and Plamitamide are elevated in HCC patients, however Stearamide appears to be more significant for individuals with cirrhosis. Regarding glycerophospholipids, the most significant molecule for cirrhotic patients was PC (23:2;O), while PA 36:6, PA 34:0, and PA 42:4 was pertinent for patients with liver cancer. The control group exhibits elevated levels of LysoPC (19:3), acylcarnitines C16:1, C16:2, C14:0, as well as DG (35:1) and DG (33:4). Nonetheless, MGDG (34:3) is more prominently expressed in the HCC cohort. In relation to sphingolipids, CerPE(d16:2/24:1(2OH)) was elevated in the HCC group, whereas Sphingosine 18:2; O2, Cer(t18:1(6OH)/16:0(2OH)), C19 Sphingosine-1-phosphate, and CerPE(d14:2/16:0(2OH)) exhibited larger levels in the cirrhosis category. Patients with hepatocellular carcinoma show higher amounts of 25-Hydroxivitamin D2, Epoxy PGE1, PGF1α, Spermidine, N-Stearoyl phenylalanine, and N-Oleoyl ethanolamine, while those with cirrhosis presented increased concentrations of Cortisol, Alpha-androstenol, Dihydrocorticosterone, Corticosterone, Hydroxycortisone, Dihydroxycholesterol, Alpha-Tocotrienol, HETE-Ethanolamine, D-Glucose, and 5-Hydroxmethyluracil.

#### 3.3.2. Biomarker Analysis for Each Class of Metabolites

For each class of molecules, the biomarker analysis revealed the receiver operating curve (ROC) and the values of the area under the ROC (AUC). Higher values are correlated with larger predictions as a biomarker. Table 5 shows the range of AUC values (cutoff *p*-values of 0.01) and the relative variation in the most representative molecules in the CIR and HCC groups. This table summarizes the detailed data presented in Table S3 (attached as a Supplementary File) where, for each class of molecules, the molecules with the highest AUC values, *p*-values, and log2FC values are presented, as well as theirrelative variation in the CIR vs. HCC group.

### 3.4. Integration of Data from Multivariate and Biomarker Analysis

**Figure 5:**
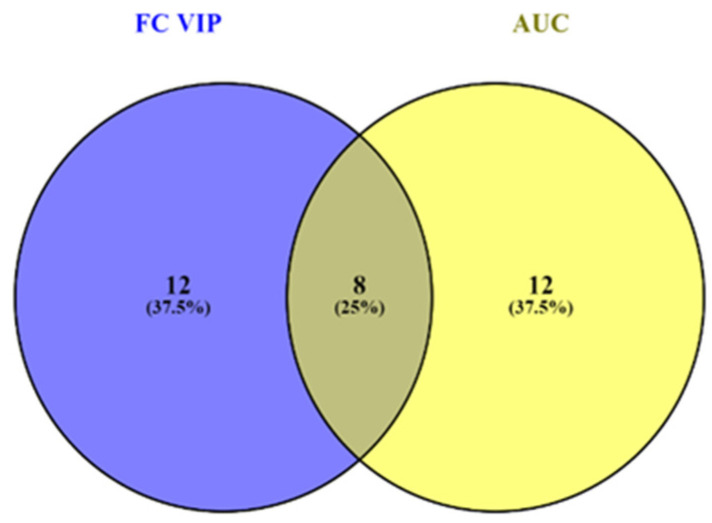
Results of Venny statistics: 8 molecules were commonly selected by untargeted and biomarker analysis.

The integration and filtration of the most relevant results was performed by Venny 2.1., an interactive tool for comparing lists of molecules, showing the Venn diagrams and identifying the common molecules which were found as representative by multivariate analysis (VIP scores and FC values) and biomarker analysis (Figure 5).

The most representative eight molecules selected by both untargeted multivariate analysis and biomarker analysis were D-Glucose and 5-Hydroxymethyluracil (HCC < CIR) and GlcCer(d18:1/12:0), PA(16:0/16:0), CerPE(d16:2/24:1(2OH), SM(d18:0/14:0), PA(36:6), and PS (18:0/16:0) (HCC > CIR).

Figure S3 (attached as a Supplementary File) shows the Venny statistical comparisons for the most representative molecules from each class of metabolites, selected by semi-targeted analysis and biomarker analysis.

## 4. Discussion

The expanding literature on lipid metabolism in hepatocellular carcinoma and cirrhosis pathogenesis has revealed new possible intervention targets and has enhanced our comprehension of lipid metabolism in these illnesses [18,27,28,29]. Lipids play several roles in cell biology, including membranes composition, energy provision for various cellular processes through fatty acid oxidation, and involvement as signaling molecules in transduction pathways. Alterations in lipid metabolism significantly contribute to carcinogenesis, involving a diverse array of lipids that serve crucial functions as energy sources or as building blocks for cell proliferation, apoptosis resistance, and immunodeficiency. The dysregulation of lipid metabolism in the etiology of HCC is increasingly acknowledged [18,27,28,29,34,35,36,37,38]. The most affected classes included glycerophospholipids (notably PC, PE, and PA), sphingolipids (SM, Cer, GlcCer), and mono- and diglycerides, each reflecting distinct aspects of hepatocellular metabolic remodeling.

The objective of our research was to contribute to the current literature and to identify potential biomarkers for individuals with liver cirrhosis at increased risk of developing HCC. We evaluated the lipidomic profiles of 81 patients, including 41 individuals diagnosed with hepatocellular carcinoma awaiting surgical or ablative treatment, and 40 diagnosed with C–P A or B cirrhosis hospitalized for investigations or follow-up. By UHPLC-MS analysis, 322 relevant molecules were selected from these patients, including 15 molecules that may serve as possible biomarkers of differentiation, exhibiting elevated levels in the cancer cohort. The current study demonstrated some significant differences between the HCC and CIR groups. Patients with HCC had elevated concentrations of sphingolipids (CerPE(d16:2/24:1(2OH)), SM(d18:0/14:0), and GlcCer(d18:1/12:0)), glycerophospholipids (PA(36:6), PA(42:4)), mono- and diglycerides (MGDG(34:3); DG(34:4)), and sterols like 25-Hydroxyvitamin D2 and Deoxycholic acid, while cirrhotic patients demonstrated elevated levels of D-Glucose and 5-Hydroxymethyluracil [39]. Fatty acids derivatives and lysophospholipids seemed to have a similar distribution amongst the two analyzed groups, with an increase in specific free fatty acids for cirrhotic patients (Docosapentenoic acid C22:5 and Palmitoleic acid C16:1). Therefore, this research emphasized the presence of sphingolipids, especially ceramides and glycerophospholipid molecules, in patients with HCC as opposed to cirrhotic patients, highlighting changes in lipid metabolism that can significantly contribute to HCC development.

Glycerophospholipids (GPLs) are a well known heterogeneous category of membrane lipids, characterized by a glycerol backbone attached to fatty acid acyl chains, and optionally connected to a polar head group, including choline, ethanolamine, inositol, and serine [18]. According to Tan et al., lipidomic profiles of HCC patients demonstrate a reduction in GPLs containing polyunsaturated fatty acids, such as arachidonic acid (C20:4) or Hydroxy-Eicosatetraenoic acid, a metabolite of C20:4, which is also expressed in our study. Furthermore, these changes come along with an increase in molecules containing monounsaturated fatty acids, such as C18:1, a long chain acylcarnitine [18,40,41]. The current study found an increase in acylcarnitines, such as C18:1;O2, which play roles in inflammation, and cell damage, all of which are associated with HCC progression, as well as free fatty acids such as stearic acid (C18:0) [18,40,41].

It is uncertain why PUFAs are depleted in HCC, especially since long chain acyl CoA synthetase 4, which activates arachidonic acid for phospholipid biosynthesis or oxidation, and fatty acid desaturase 2, which participates in long chain PUFA synthesis, have both been shown to be enhanced in HCCs. One probable argument is that PUFAs are destroyed by reactive oxygen species [18,42,43,44,45]. Oxidized phospholipids accumulate during NASH progression in mouse models, suggesting that a high oxidative damage burden may lead to hepatocarcinogenesis and PUFA peroxidation [46]. This study also suggests a contribution of the oxidative stress, which may be reflected by the presence of hydroxy-eicosapentaenoic acid and oxylipins (PGF1α, Epoxy-PGE1). Although we have not directly quantified oxidized phospholipids, LysoPCs can indicate membrane degradation caused by phospholipase A2 activation, in agreement with previous data [46,47]. The increase in MUFAs in HCC can be attributed to an increased expression of stearoyl-CoA desaturase (SCD), which converts saturated fatty acids into MUFAs. Lipidomic investigations of human HCC show a higher MUFA-to-PUFA ratio in the GPL composition of malignancies, a pattern which may be attributed to oxidative stress. However, as mentioned below, this could indicate an intentional rewiring of tumor metabolism to promote development. The enrichment in monounsaturated species (C18:1) and the depletion of highly unsaturated chains (C20:4, C22:6) suggest a shift toward enhanced SCD1 and reduced fatty acid desaturase 2 activity, reflecting a reconfiguration of desaturation and elongation of pathways characteristic of tumor metabolism [18,36,38,48].

Ferroptosis is a form of cell death that occurs following lipid peroxidation; therefore, lipid metabolism is particularly involved in this process. This type of cell death can be suppressed by iron chelators, lipophilic antioxidants, inhibitors of lipid peroxidation, and PUFAs depletion. Moreover, ferroptosis has been associated with degenerative disease, carcinogenesis, stroke, traumatic brain injury, kidney degeneration, and ischemia-reperfusion injuries [42]. PUFAs, which include easily extracted bis-allylic hydrogen atoms, are prone to lipid peroxidation and are required for the occurrence of ferroptosis; thus, the number and distribution of polyunsaturated fatty acids determines the degree of lipid peroxidation and the rate at which ferroptosis is effective. Lipidomic studies reveal that phosphatidylethanolamines (PEs) carrying arachidonic acid or its elongation product, adrenic acid (C22:4), are crucial phospholipids that undergo oxidation and push cells toward ferroptotic death [18,38,42,49]. The reduced involvement of PUFAs into membrane phospholipids has been shown to promote a resistance to ferroptosis. Moreover, MUFA in a high quantity, promoted by SCD activity, is protective against it, promoting cancer cell proliferation and survival. SCD1 overexpression occurs in response to chemotherapeutics, which promotes chemoresistance in HCC cells. Recent research indicates that cancer cells require SCD1-driven MUFA production to thrive in low-lipid conditions [18,42]. Additionally, studies show that certain oxylipins, including the pro-inflammatory 20-HETE, are essential in ferroptosis and are enhanced in cirrhosis, correlating with disease severity and negative outcomes such as increased mortality. This is associated with their function in facilitating chronic inflammation and fibrosis in the liver. The present research identified several oxylipins (as detailed in Table S1 and Table 5), which may be regarded as key elements of ferroptosis, and precursors to cirrhosis [50].

Sphingolipids are a class of lipids, highly expressed in our research as HCC markers, that contain an aliphatic amino alcohol chain, like sphingosine. Ceramides are composed of a sphingosine molecule attached to a fatty acid chain [18,37,51,52]. There are research papers that have shown a decrease in ceramides in patients with HCC [53,54]. However, some have reported the accumulation of ceramides in human HCC specimens, like our current study [37,55]. Although there is no clear understanding of these discrepancies, research suggests an increase in ceramide flux to alternate destinations such as sphingomyelin, S1P, and glycosphingolipid accumulation in hepatocellular carcinoma [18,37]. The formation of glucosylceramide (GlcCer) from ceramides is elevated in hepatocellular carcinoma, as indicated by Miura et al., with various hexosylceramide species that are markedly elevated in human HCC. Ceramides are recognized for their role in mediating apoptosis, influencing cell growth inhibition, promoting apoptosis, regulating senescence, and facilitating autophagy [18,37].

Both the tissue and serum levels of sphingolipid metabolites were reported to be upregulated in patients with HCC, as opposed to normal tissue or compared with cirrhotic patients [37]. Some metabolites, such as C16-ceramide and S1P, were eventually proposed as novel diagnostic indicators for patients with HCC with liver damage, as confirmed by our research. Moreover, Miura et al. identified HexCer and SM as overexpressed in liver tumor tissue, which indicates that these metabolites have a significant role in HCC, despite their established suppressive role in cancer [37]. As previously stated, there are studies that report contradictory findings, such as Uranbileg et al. [55] and Grammatikos et al. [56], who showed increased levels of ceramide in the serum of patients with HCC, while Cai et al. [57] reported a high expression of SphK in hepatocellular carcinoma tissue. Like other malignancies, S1P is higher in tumor tissue compared to healthy tissue [58].

Ceramide, sphingolipid, and glycerophospholipid levels are elevated in HCC, indicating a shift toward increased membrane synthesis (necessary for accelerated tumor proliferation), improved lipid storage and metabolic adaptability, and increased fatty acid oxidation to meet cancer cells’ energy requirements [18,23,37,38].

Reduced D-glucose levels in HCC demonstrate the Warburg effect (aerobic glycolysis), supporting the hypothesis of metabolic reprograming in cancer cells [18,20,21]. Furthermore, our study has a large lipid class coverage, analyzing 11 distinct metabolite categories by both untargeted and semi-targeted approaches, allowing a more accurate class-level profiling as compared to other studies [17,29].

The increase in a panel of 20 lipids involving phosphocholine, ceramide, fatty acyl carnitine, lysophosphocholine, sphingomyelin, and lysophosphatidylinositol was observed in Lu et al. [28]. This study involved 50 HCC patients paired with 24 healthy individuals. In the HCC group, 46 subjects were affected by HBV and one was positive for anti-hepatitis C virus. The research was conducted on liver tissues collected from the oncological patients and serum collected from each subject. The lipid signatures with diagnostic and prognostic potential were evaluated in an independent batch of serum samples. A total of 20 hepatic and 40 serum lipid signatures were found; nevertheless, there was a minimal statistical association between them. The findings revealed that triglycerides and phosphatidylethanolamine-based plasmalogens (PEp) predominantly affected altered serum lipids. In serum, Pep (36:4) and (40:6) showed a notable ability to differentiate HCC patients from healthy controls and were substantially correlated with HCC tumor grades (*p* < 0.05), thus qualifying them for consideration as possible diagnostic and prognostic biomarkers for HCC. In addition, Lu et al. focused on early diagnosis, whilst our research used an untargeted and semi-targeted workflow to distinguish cirrhotic and HCC patients [28,36].

Rashid et al. [59] showed the importance of lipidomics in distinguishing 20 patients diagnosed with HCC, who had underlying LC vs. 20 LC patients. Most patients had HCV. The mean age of the patients was similar to our cohort, between 58 and 59 years old. Patients with HCC had higher levels of xanthine and uric acid, indicating a higher ROS production, therefore influencing HCC evolution. While Rashid et al. reported high levels of lysophosphatidylethanolamines and triglycerides, our study showed an increase in sphingolipids, glycerophospholipids, diacylglycerols, phosphatidic acids, and lysophosphatidylinositols. Phosphatidylethanolamines were elevated in both studies. Fatty acids and bile acids were decreased in Rashid et al. [59], while polar metabolites were downregulated in our research. Furthermore, both studies demonstrated a strongly altered lipid metabolism in HCC and LC [59]. Cohort differences and methodology would explain the discrepancies, but each one of the studies points out that major lipid remodeling is a hallmark in HCC development.

Powell et al. focused on the early detection of HCC in patients with cirrhosis. The analytical approach of the current research and the study conducted by Powell et al. was untargeted LC–MS-based lipidomics with high resolution separation and mass accuracy, showing an upregulation in triglycerides (TG) species in the HCC group, while lysophospholipids and certain sphingomyelins were reduced. Despite the varying sampling timelines, the overlap in altered lipid classes (higher triglycerides and lower levels of lysophosphatidylcholines and sphingomyelins) improves the repeatability of these biomarkers across disease phases. The similarities of the findings suggest membrane remodeling and altered phospholipid turnover, with an emphasis on energy storage dysregulation in HCC regarding our study [29].

Lipidomic and metabolomic research may yield analogous or contradictory results due to various barriers. To ensure robustness, the classification models were internally cross-validated, showing high accuracy (R^2^ > 0.9, Q^2^ ≈ 0.8) and consistent discrimination between groups. Receiver operating characteristic analysis confirmed strong diagnostic performance for the top molecules, with AUC values exceeding 0.90. There are several factors that can alter lipidomic profiles, such as diet, BMI, microbiome, gender, age, ethnicity, and comorbidities [60,61,62,63]. Several of the identified lipid changes are connected to organelle-specific dysfunctions. The buildup of phosphatidic acid and diacylglycerol indicates increased endoplasmic reticulum-lipid droplet signaling and membrane curvature stress, while altered sphingolipid profiles imply disrupted plasma membrane dynamics. Reduced polyunsaturated phospholipids and acylcarnitines suggest mitochondrial β-oxidation overload. These findings support the idea that HCC encompasses the coordinated alteration of membrane and energy-related lipid networks across many organelles [18,23,37,64,65].

### Study Limitations

This research has several limitations that should be acknowledged. The restricted cohort of individuals arises from the limited number of patients with an early diagnosis of HCC, which is the primary rationale for our investigation into this specific topic, but also because they were recruited from a single healthcare center. The sample size was modest, and comprehensive data regarding lifestyle, diet, and genetic background—factors that potentially affect lipid metabolism and variability—was unavailable [26,27,60,66]. The analysis was performed exclusively in the positive ESI mode; therefore, some lipid subclasses that ionize more efficiently in a negative mode may be underrepresented. In addition, while multivariate models were internally cross-validated and demonstrated high accuracy, an external validation cohort was not included. Finally, forthcoming multicentric studies using supplementary ionization modes, organelle-specific lipidomics, and larger, clinically representative cohorts are warranted to confirm and extend these observations.

## 5. Conclusions

The present study highlights significant alterations in lipid metabolism between individuals with hepatocellular carcinoma and cirrhosis, expressed by sphingolipids, glycerophospholipids, sterols, and specific fatty acids, that can be considered as putative biomarkers. These findings reflect the metabolic reprogramming characteristic of HCC, supporting membrane remodeling, enhanced energy production, oxidative stress adaptability, and resistance to ferroptosis. Several detected metabolites demonstrated high predictive accuracy and were consistent with previously reported results, reinforcing their potential clinical utility. Integrating lipidomic profiling into current diagnostic and prognostic strategies could improve early detection and risk stratification in cirrhotic patients. Future validation in larger, multicenter, and longitudinal cohorts using complementary ionization lipidomics, metabolic flux tracing, and organelle-targeted analyses remains essential to confirm these results and also to explore their therapeutic implications.

## Figures and Tables

**Table 1:** Different statistical analyses applied in this study: untargeted multivariate analysis of 322 common metabolites found in the cirrhosis (CIR) and HCC groups, followed by semi-targeted analysis of 11 classes of metabolites, mainly lipids.

Untargeted multivariate analysis: comparison of metabolic fingerprints between the CIR and HCC groups (322 metabolites with MW 120–760 Da)	➢ Volcano plot fold-change values (*p* < 0.1) and correlation *T*-test (*p* < 0.05) ➢ PLSDA and loadings (VIP scores—top 15 molecules) ➢ Cross validation (accuracy, R2 and CV) ➢ Heatmap including dendrogram ➢ Random Forest and MDA scores—top 15 molecules (classification and prediction) ➢ Biomarker analysis (AUC values) ➢ DSPC Network (from KEGG database) ➢ Venny 2.1 diagram to select and identify common molecules found as representative by multivariate analysis (VIP scores and FC values) and Biomarker analysis
Semi-targeted analysis: comparison of the profiles of 11 metabolite classes. Free fatty acids (FFAs) (n = 53) Fatty acid derivatives (n = 17) Glycerophospholipids (n = 36) Lysophospholipids (n = 32) Acylcarnitines (n = 25) Mono- and diglycerides (n = 22) Sphingolipids (n = 37) Sterol lipids (n = 33) Oxilipins (n = 11) Antioxidant lipids (n = 5) Polar molecules (n = 51)	

**Table 2:** Demographic, paraclinical, and clinical characteristics of study population (HCC vs. Cirrhosis group).

Parameters	HCC Group (n = 41)	Cirrhosis Group (n = 40)	*p*-Value
Age	66.95 (±9.02)	60.03 (±8.87)	0.001
Sex, no (%) **			0.94
Female	11 (28.6%)	11 (27.5%)	
Male	30 (73.2%)	29 (72.5%)	
Environment (%) **			0.58
Rural	12 (29.3%)	14 (35%)	
Urban	29 (70.7%)	26 (65%)	
Etiology (%) **			0.001
HCV	11 (26.8%)	5 (12.5%)	
HBV	7 (17.1%)	5 (12.5%)	
Alcohol intake	8 (19.5%)	30 (75%)	
Deceased (%)			0.58
Yes	9 (21.9%)	7 (17.5%)	
No	32 (78.1%)	33 (82.5%)	
ALAT *	26.3 (19.7–46.5)	28.8 (21–51.9)	0.74
ASAT *	32 (26–50.5)	53.1 (32.9–97.6)	0.11
TB *	0.7 (0.5–1.35)	1.79 (0.88–2.85)	0.04
Albumin *	4.33 (0.47)	3.63 (0.61)	0.001
Platelet count *	180.05 (84.54)	142.75 (85.96)	0.06
AFP *	5.27 (2.64–37.6)	4.13 (2.30–6.39)	0.34
INR *	1.15 (0.19)	1.40 (0.28)	0.03
Triglycerides *	100.80 (64.54)	83.57 (37.18)	0.12
Cholesterol *	155.78 (63.74)	134.10 (43.42)	0.14
Ascites **			0.001
Yes	6 (14.6%)	18 (45%)	
No	35 (85.4%)	22 (55%)	
Child-Pugh classification **			0.001
A	21 (51.2%)	16 (40%)	
B	6 (14.6%)	24 (60%)	
without cirrhosis	14 (34.1%)	0	
HVPG *	8.72 (4.21)	12.88 (3.27)	0.02
TE-LSM *	14.49 (13.80)	51.49 (25.63)	0.001
TAC score		N/A	N/A
very low	1 (2.4)		
low	9 (22)		
medium	27 (65.9)		
high	4 (9.8)		
BCLC **		N/A	N/A
0	4 (9.2%)		
A	34 (82.9%)		
B	3 (7.3%)		
MELD score **			0.001
≤9	24 (58.5%)	5 (12.5%)	
10–19	17 (41.5%)	35 (87.5%)	
Milan criteria **		N/A	N/A
In	13 (31.7%)		
Out	28 (68.3%)		

* = values expressed as median IQR; ** = values expressed as absolute value + per cents; HCC = hepatocellular carcinoma; *p* = level of significance; N/A = not assessed; HCV = hepatitis C virus; HBV = hepatitis B virus; ALAT = alanine aminotransferase; ASAT = aspartate aminotransferase; TB = total bilirubin; AFP = alpha-fetoprotein; INR = international normalized ratio; HVPG = hepatic venous pressure gradient; TE-LSM = transient elastography liver stiffness measurement; TAC score = tumor burden score, alpha-fetoprotein levels, and Child–Pugh classification; BCLC = Barcelona Clinic Liver Cancer; MELD = model for end-stage liver disease.

**Table 3:** The VIP scores, fold-change (FC), log2(FC), *p*-values (as determined by volcano plot and *T*-test), and RF ranking of molecules which may discriminate the CIR and HCC groups. The relative variation is mentioned in each case.

*m*/*z*		VIP	FC	log2FC	*p*-Value	MDA	Relative Variation
181.1081	D-Glucose	1.903	2.069	1.049	2.88 × 10^−15^	<0.07	HCC < CIR
167.0921	Methylxanthine	1.762	2.008	1.01	1.65 × 10^−12^	<0.07	HCC < CIR
143.0953	5-Hydroxymethyluracil	1.967	1.863	0.898	2.00 × 10^−19^	<0.07	HCC < CIR
595.3371	LysoPI 18:3	1.781	0.715	−0.484	8.77 × 10^−9^	0.0113	HCC > CIR
638.5631	Cer(d16:2/24:0(2OH))	1.070	0.715	−0.482	3.13 × 10^−4^	0.0074	HCC > CIR
600.4239	DG(34:0)	1.843	0.681	−0.554	4.25 × 10^−12^	<0.07	HCC > CIR
644.4472	GlcCer(d18:1/12:0)	1.857	0.624	−0.680	9.47 × 10^−13^	0.0082	HCC > CIR
688.4706	PE 32:2	1.756	0.568	−0.815	2.41 × 10^−11^	0.0112	HCC > CIR
713.3923	CerPE(d14:2/24:1)	1.953	0.551	−0.859	6.30 × 10^−15^	0.0111	HCC > CIR
628.4536	DG 36:0	1.829	0.549	−0.866	1.50 × 10^−12^	0.0095	HCC > CIR
581.3243	LysoLPC 22:1	1.263	0.490	−1.026	2.01 × 10^−11^	0.0073	HCC > CIR
589.3852	LysoPI O-18:0	1.781	0.461	−1.118	7.67 × 10^−13^	0.0078	HCC > CIR
669.3689	DG 40:6	1.838	0.459	−1.124	1.50 × 10^−8^	0.0131	HCC > CIR
649.4018	PA(16:0/16:0)	1.799	0.452	−1.147	3.63 × 10^−13^	0.0105	HCC > CIR
732.4949	PC(16:0/16:1)	1.006	0.433	−1.21	2.63 × 10^−12^	0.0083	HCC > CIR
757.4159	CerPE(d16:2/24:1(2OH))	2.144	0.419	−1.256	4.28 × 10^−22^	0.0130	HCC > CIR
677.4322	SM(d18:0/14:0)	1.678	0.402	−1.31	4.08 × 10^−11^	0.0100	HCC > CIR
693.4258	PA(36:6)	1.866	0.377	−1.409	1.80 × 10^−14^	0.0163	HCC > CIR
765.4801	PS(18:0/16:0)	1.786	0.326	−1.615	6.27 × 10^−13^	0.0122	HCC > CIR
781.4709	PA 42:4	1.774	0.279	−1.840	1.01 × 10^−12^	<0.07	HCC > CIR

**Table 4:** The *m*/*z* values, area under the curve (AUC), *p*-values and log2FC values for the putative biomarkers in blood serum of CIR vs. HCC group.

Identification	AUC	*p*-Value	Log2 FC	Relative Variation
CerPE(d16:2/24:1(2OH))	1	1.51 × 10^−20^	−1.014	HCC > CIR
Cer(t18:0/20:0(2OH))	0.991	1.01 × 10^−12^	−0.636	HCC > CIR
5-Hydroxymethyluracil	0.990	4.27 × 10^−19^	0.946	HCC < CIR
PA(36:6)	0.988	1.04 × 10^−13^	−1.132	HCC > CIR
DG(34:4)	0.981	5.37 × 10^−12^	−0.852	HCC > CIR
PA(16:0/16:0)	0.980	2.64 × 10^−12^	−0.896	HCC > CIR
D-Glucose	0.976	2.07 × 10^−16^	1.127	HCC < CIR
PA(34:4)	0.975	8.97 × 10^−13^	−0.564	HCC > CIR
DG (44:12)	0.973	4.60 × 10^−15^	−0.671	HCC > CIR
PS(18:0/16:0)	0.969	2.50 × 10^−12^	−1.340	HCC > CIR
5-Methoxytryptophan	0.963	2.13 × 10^−8^	0.628	HCC < CIR
SM(d18:0/14:0)	0.962	1.51 × 10^−10^	−1.047	HCC > CIR
GlcCer(d18:1/12:0)	0.962	8.46 × 10^−13^	−0.502	HCC > CIR
Palmitoleyl linolenate	0.955	7.82 × 10^−11^	−0.583	HCC > CIR
Cer(t18:0/18:0(2OH))	0.955	3.36 × 10^−12^	−0.385	HCC > CIR

**Table 5:** The AUC value ranges for each class of molecules which, according to biomarker analysis, can be considered as putative biomarkers of differentiation between the CIR and HCC groups, with their relative variation (HCC > CIR vs. HCC < CIR).

Metabolite Classes	AUC	HCC > CIR	HCC < CIR
Free fatty acids	0.934–0.742	C17:1; C38:0; C40:6; C20:5-O; C18:0; C14:0	C22:5; C14:2; C30:3; C16:1; C22:6
Fatty acid derivatives	0.810–0.503	Linoleyl arachidate; Linoleyl linoleate; Linoleyl arachidonate; Stearyl stearate; Oleyl palmitate	Stearamide; Docosenamide; Amino-octanoic acid; Myristyl palmitate; Myristoleyl arachidonate
Glycerophospholipids	0.877–0.648	PA 42:4; PS 34:0; PA 38:6; PG O-34:4; PA 36:6	PC (23:2; O); PA 30:2; PA(O-18:0/16:0); PA 23:0; PE 30:3
Lysophospholipids	0.793–0.586	LysoPC(20:3); LysoPC (22:1); LysoPC(22:6); LysoPI (18:3); LysoPA (18:1)	LysoPC (19:3); LysoPE (22:6); LysoPE 18:0); LysoPE (16:1)
Acylcarnitines	0.777–0.516	C18:1;O2; CAR 20:0; CAR 12:2; CAR 26:0; CAR 12:0;O; CAR 12:1;O; CAR 16:1;O; CAR 12:1; CAR 18:2; CAR 14:0	CAR16:2; CAR 14:0; CAR 16:1; C16:0; CAR 18:3;O
Mono- and Diglycerides	0.830–0.518	DG40:7; MGDG (34:3); MGMG (16:2); DG(42:0); DG(34:1)	DG(33:4); DG(35:1); MG(20:4); DG (44:0); DG (40:1)
Sphingolipids	0.911–0.666	CerPE(d16:2/24:1(2OH)); SM(d18:1/18:1); SM(d18:0/14:0); CerPE(d16:2/20:1(2OH)); GlcCer(d18:1/14:0); CerPE(d16:1/16:0); Cer(t18:0/20:0(2OH))	Sphingosine18:2; O2; CerPE(d14:2/16:0(2OH)); Cer(t18:1(6OH)/16:0(2OH)); C19 Sphingosine-1-phosphate; Cer(t18:0/19:0(2OH)); Cer(d18:2/20:1)
Sterol lipids	0.771–0.580	25-Hydroxyvitamin D2; 3-Oxocholic acid; 21-hydroxypregnenolone; Dihomocholic acid; Deoxycholic acid; Ketodeoxycholic acid	Cortisol; Alpha-androstenol; Dihydrocorticosterone; Corticosterone; Hydroxycortisone; Dihydroxycholesterol; 18:0 Cholesterol ester; 12-Estrone 3-sulfate; Estradiol-17beta; Cortisol 21-sulfate
Antioxidants	0.898–0.524	Ascorbyl palmitate, all-trans-retinyl oleate; beta-carotene	Alpha-Tocotrienol
Oxylipins	0.729–0.502	PGF1a; Epoxy PGE1; Hydroxy-PGF1a; 15-HETE-GABA	HETE-Ethanolamine, PGE3; PGA2; 9-HODE; Lipoxin A4
Polar molecules	0.870–0.715	N-Oleoylethanolamine; 5-Hydroxymethyluracil; Proline betaine; Phosphoserine; Trytptophan, N-stearoyl phenylalanine	N-Acetyl-D-glucosamine; N-Palmitoyltryptamine; D-Glucose; Taurine; L-Homocysteine sulfate 5-Hydroxymethyluracil

## Data Availability

The raw data supporting the conclusions of this article will be made available by the authors on request due to privacy and ethical considerations regarding the patients admitted at the “Prof. Dr. Octavian Fodor” Regional Institute of Gastroenterology and Hepatology, Cluj-Napoca, Romania.

## Supplementary Materials

The following supporting information can be downloaded at: https://www.mdpi.com/article/10.3390/biom15111575/s1, Table S1: Identification of different classes of metabolites. Table S2: The VIP scores, fold-change (FC), log2(FC), *p*-values, and RF ranking of molecules from each of the 11 metabolite classes which may discriminate between the CIR vs. HCC group. Table S3. Biomarker analysis. AUC values for each class of metabolites. Figure S1: The Debiased Sparse Partial Correlation Network. Figure S2: Comparative figures per metabolite classes. Figure S3: The most representative molecules from each class of metabolites.
