# Supplementary material for: Differential Metabolic Dysregulations in Hepatocellular Carcinoma and Cirrhosis: Insights into Lipidomic Signatures
Source: Biomolecules. 2025 Nov 10;15(11):1575. doi: 10.3390/biom15111575 (PMC12650657; doi:10.3390/biom15111575)
Supplement: Supplementary file 1 [file biomolecules-15-01575-s001.zip › Figure S1. The Debiased Spase Partial Correlation Network.pdf]

Figure S1. The Debiased Spase Partial Correlation (DSPC) Network, established according to the input data of metabolites identified in this study

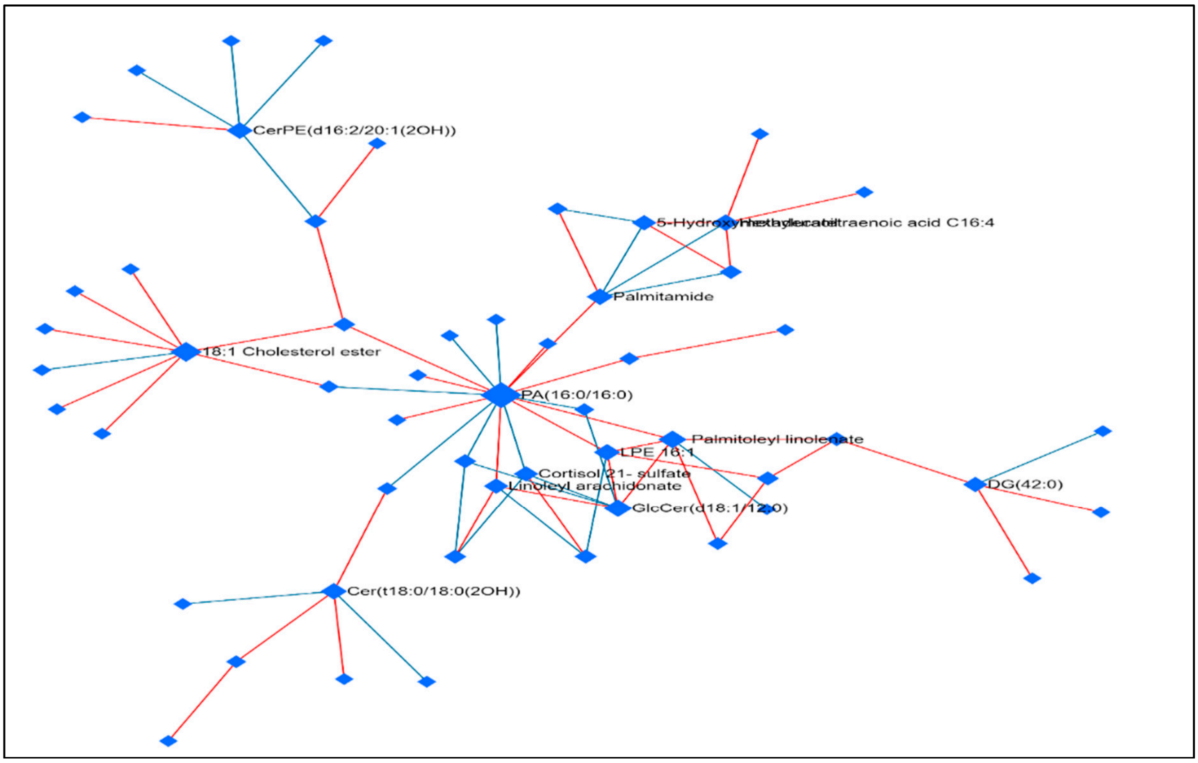

The DSPC Network, an algorithm included in the Metaboanalysys 6.0 was also applied and presents the links between the most representative metabolites in different lipid metabolic pathways, the nodes representing the input metabolites, while the edges represent the association measures. The metabolites identified are shown, as well the top correlations (edges) based on their p-value rankings (top 20%).
