# Supplementary material for: Differential Metabolic Dysregulations in Hepatocellular Carcinoma and Cirrhosis: Insights into Lipidomic Signatures
Source: Biomolecules. 2025 Nov 10;15(11):1575. doi: 10.3390/biom15111575 (PMC12650657; doi:10.3390/biom15111575)
Supplement: Supplementary file 1 [file biomolecules-15-01575-s001.zip › Figure S2. Comparative figures per metabolite classes.pdf]

Figure S2. Graphic results of semi targeted analysis for each class of metabolites identified in groups CIR and HCC: PLSDA and sPLSDA score plots, PLSDA VIP score and sPLSDA loadings, Cross Validation graph, Network links, Volcano Plot, Heatmap, Random Forest (Rf) plot.

1. Free fatty acids

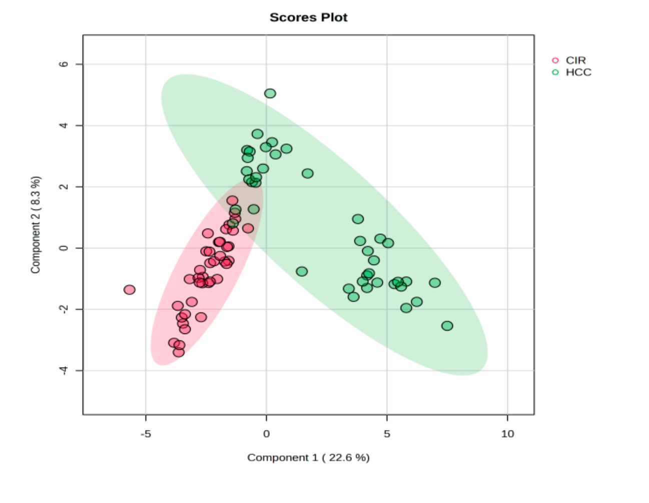

PLSDA score plot

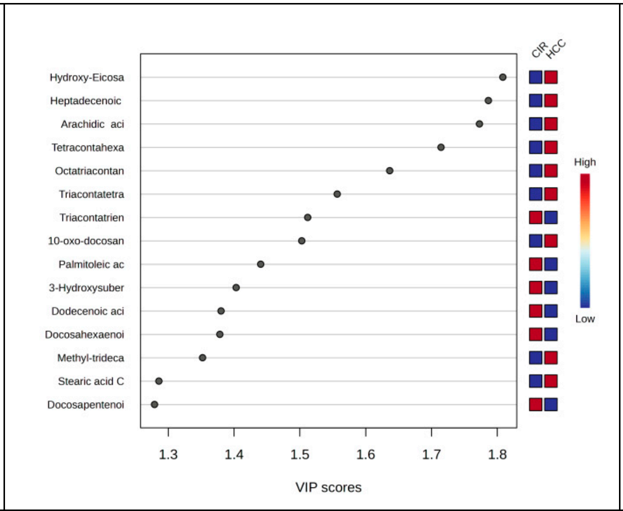

PLSDA loadings VIP score

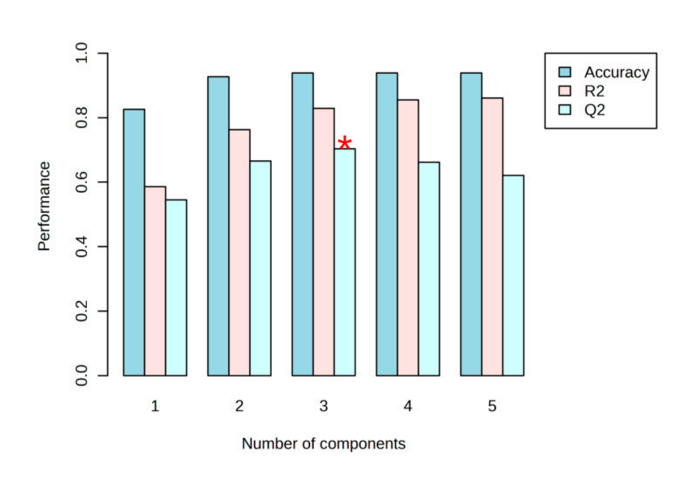

Cross Validation Graph

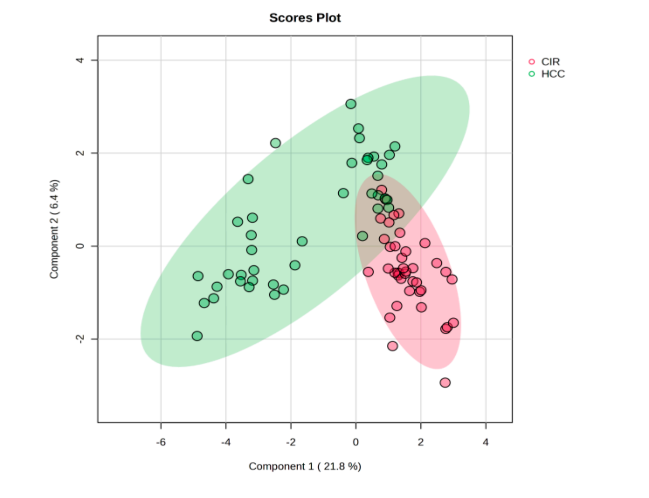

sPLSDA score plot

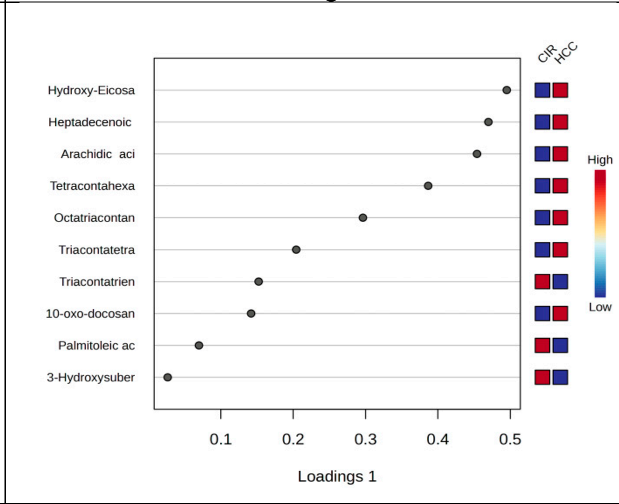

sPLSDA Loadings plot

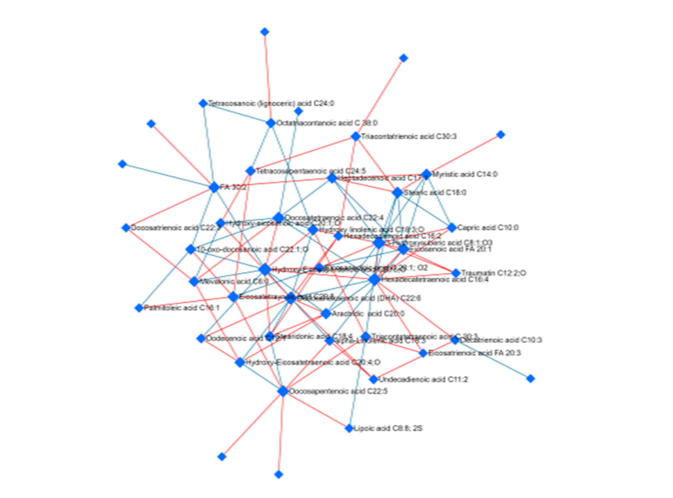

Network links

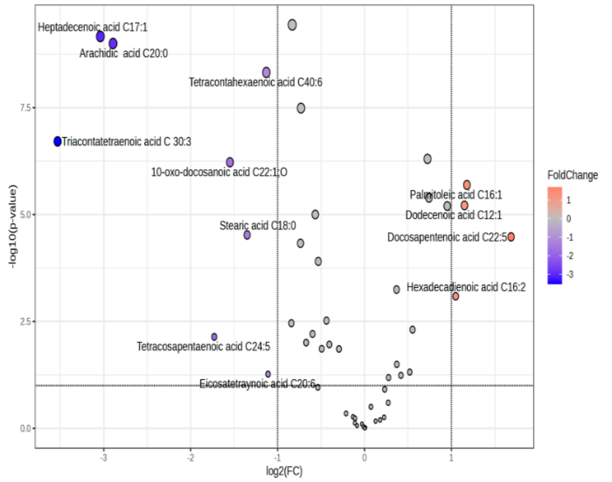

Volcano Plot

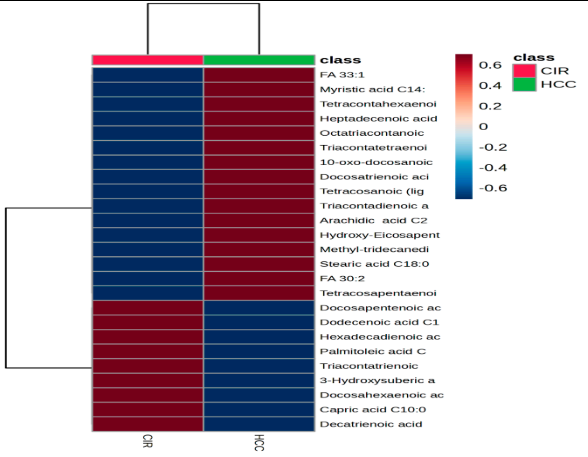

Heatmap - considering mean features per group

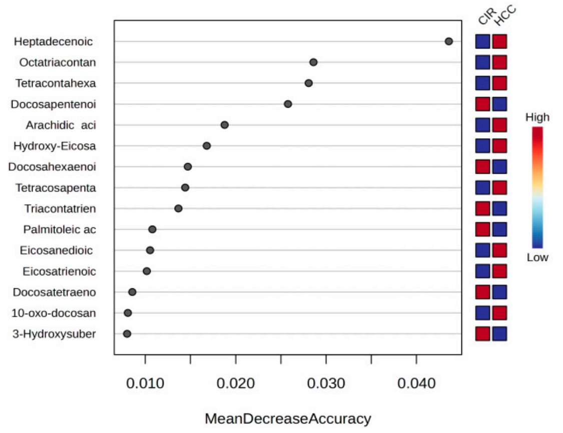

Random Forest (Rf) plot

2. Fatty acids derivatives

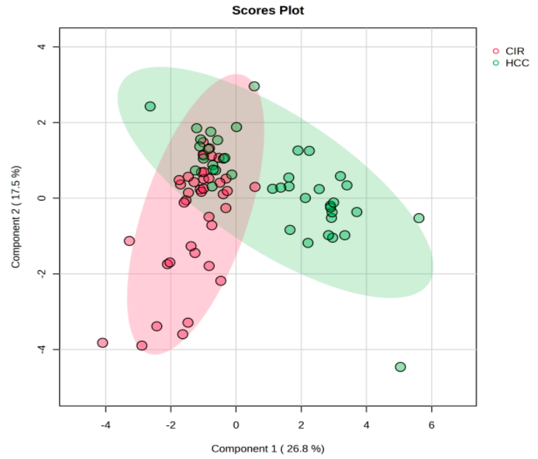

PLSDA score plot

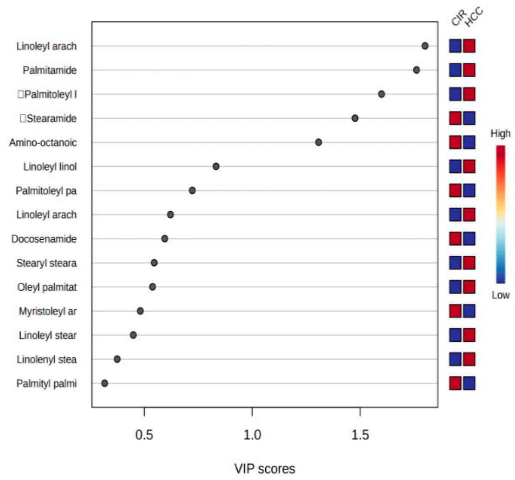

PLSDA loadings VIP score

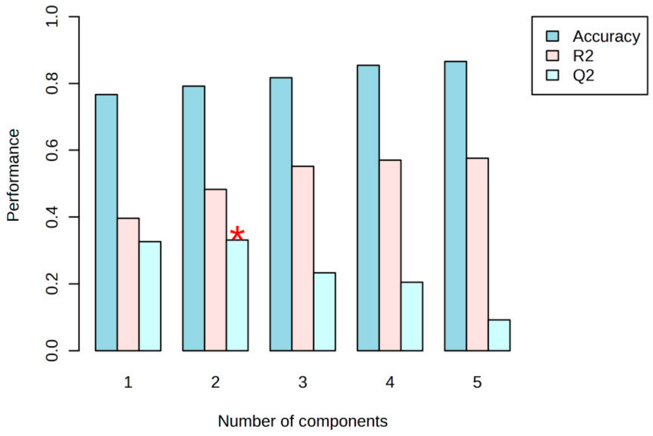

Cross Validation Graph

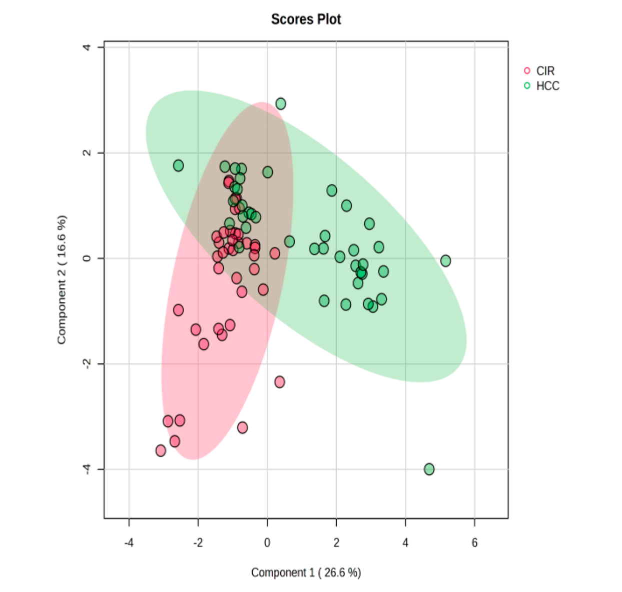

sPLSDA score plot

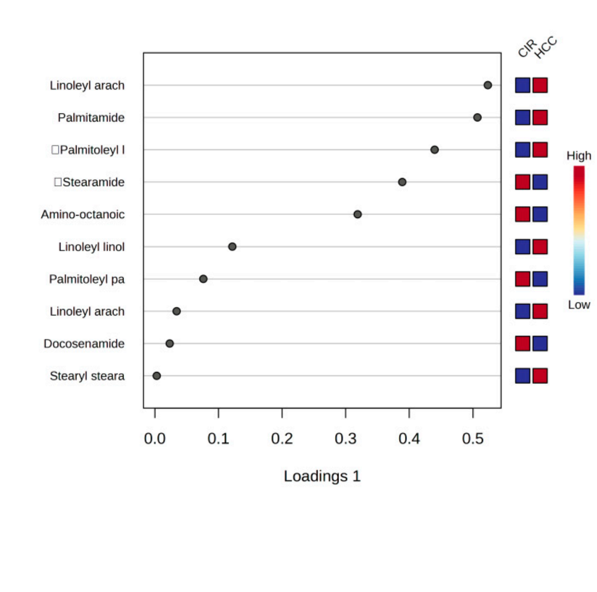

sPLSDA Loadings plot

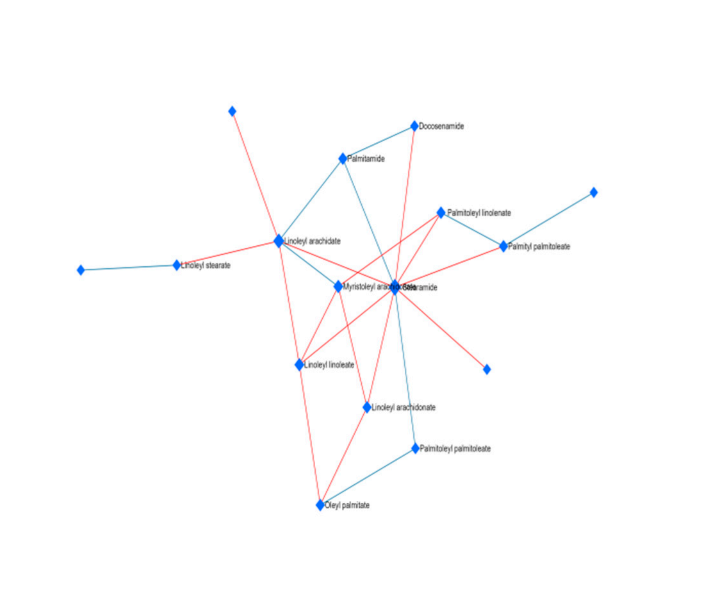

Network links

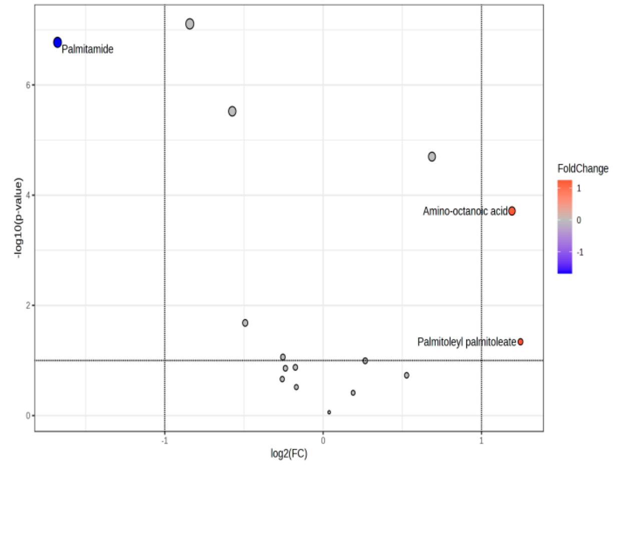

Volcano Plot

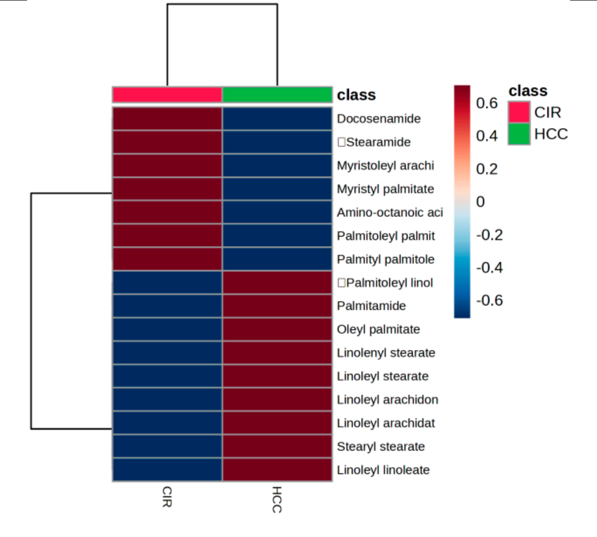

Heatmap- considering mean features per group

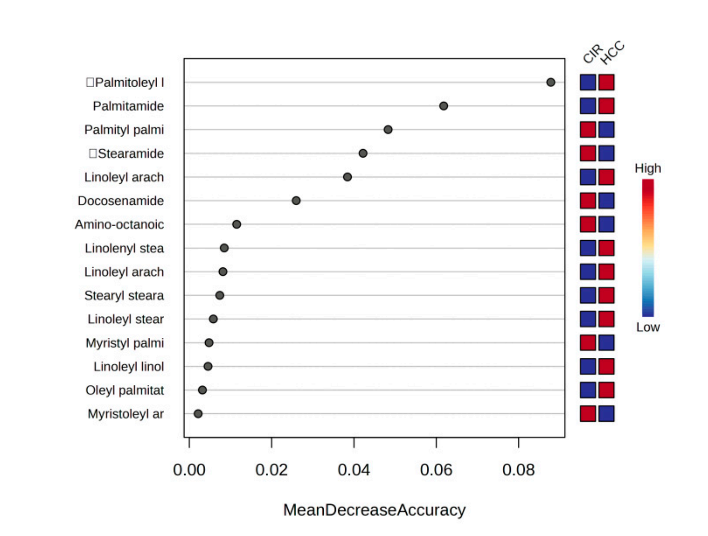

Random Forest (Rf) plot

3. Glycerophospholipids

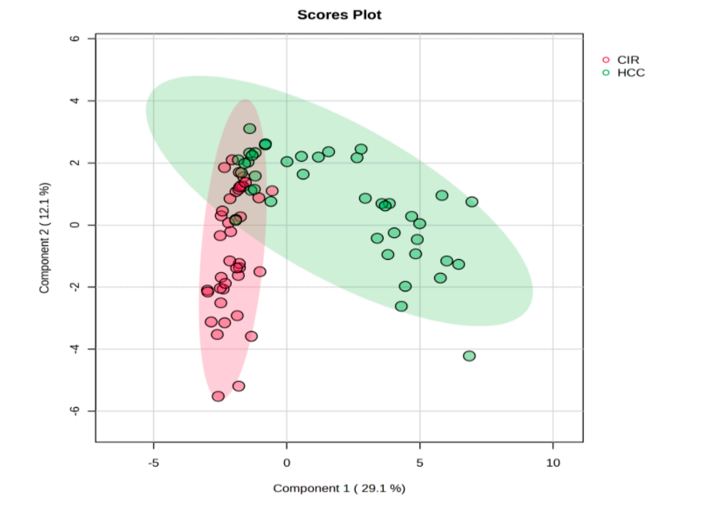

PLSDA score plot

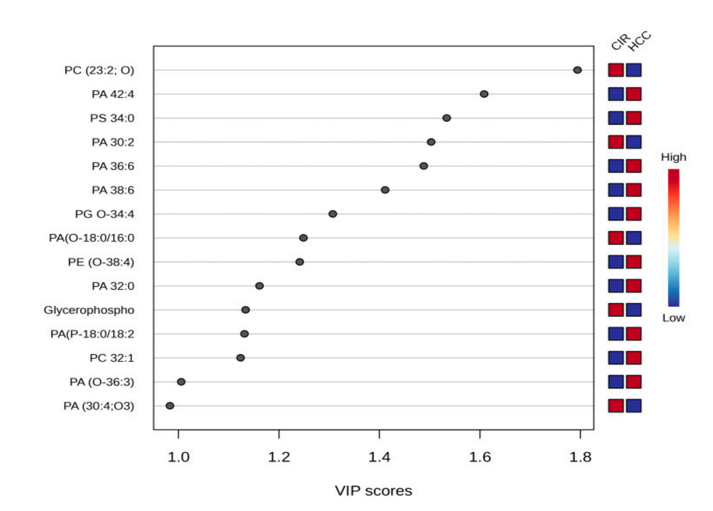

PLSDA loadings VIP score

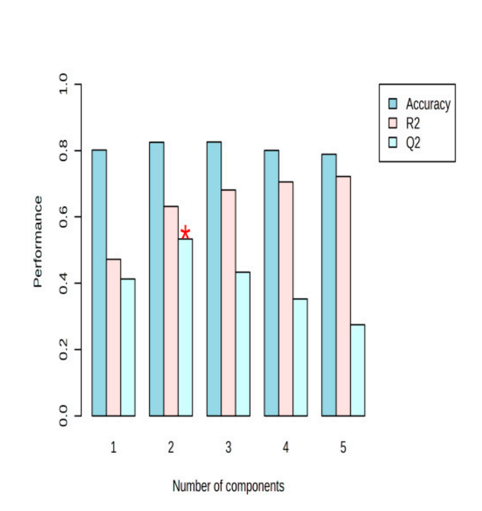

Cross Validation Graph

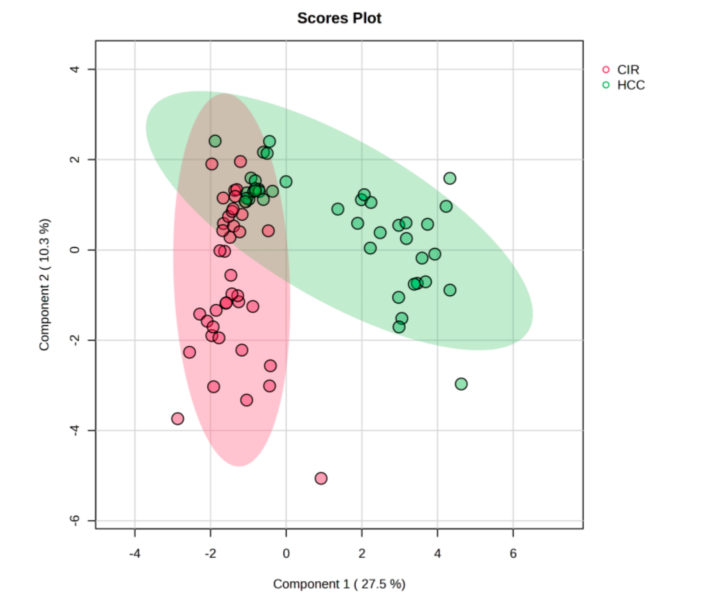

sPLSDA score plot

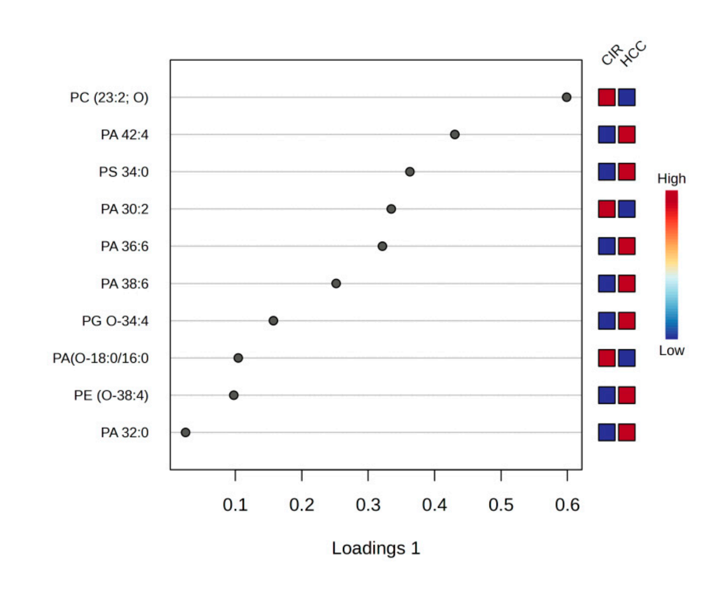

sPLSDA Loadings plot

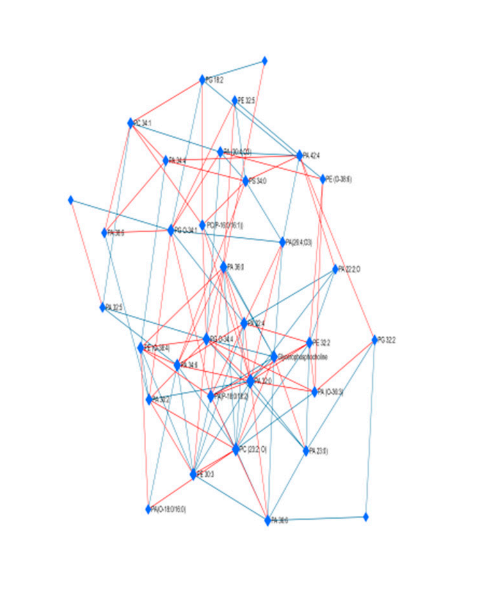

Network links

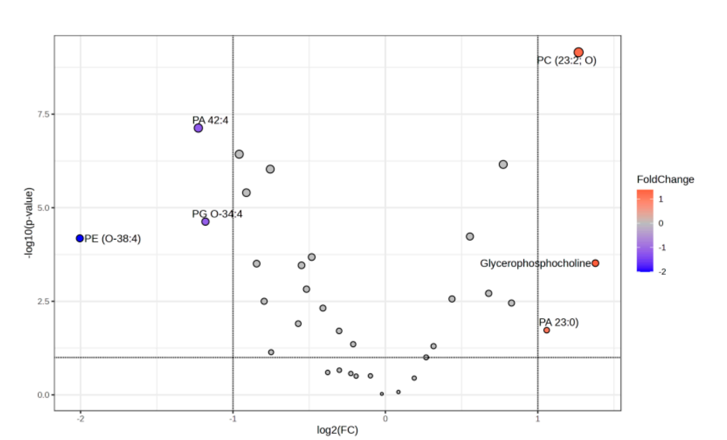

Volcano Plot

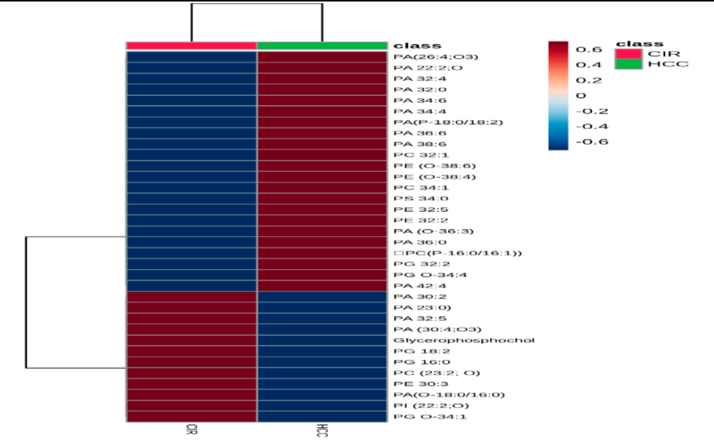

Heatmap- considering mean features per group

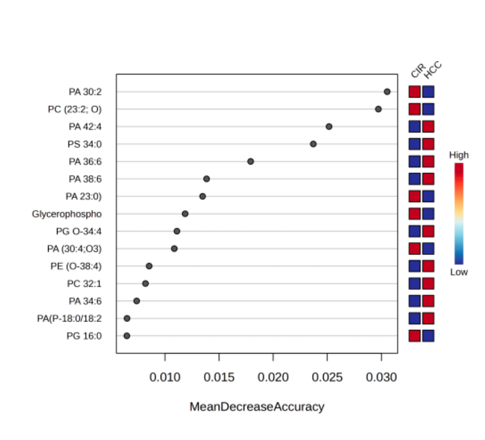

Random Forest (Rf) plot

4. Lysophospholipids

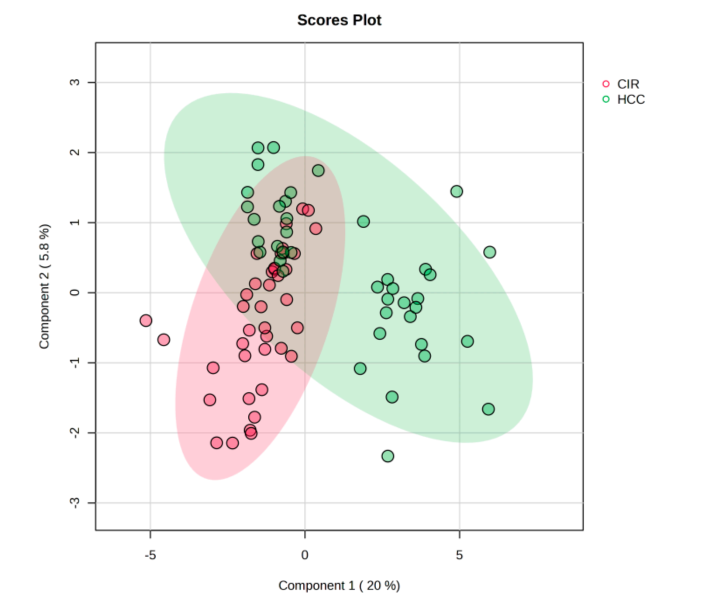

PLSDA score plot

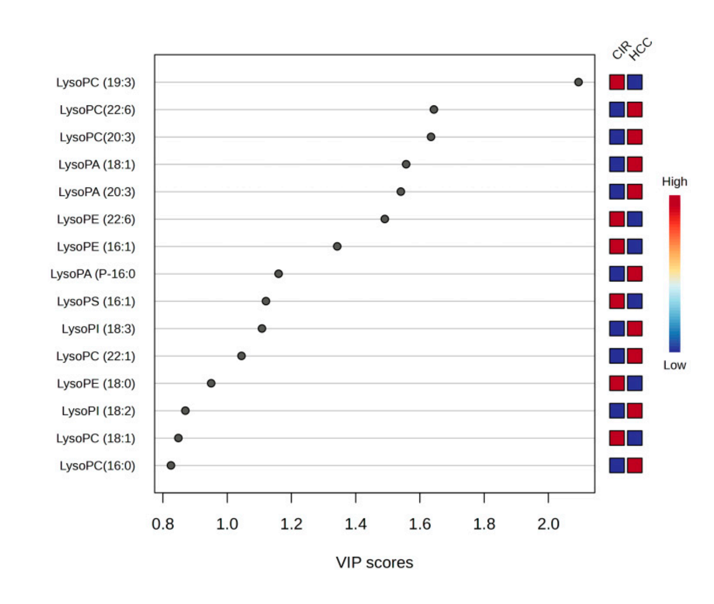

PLSDA loadings VIP score

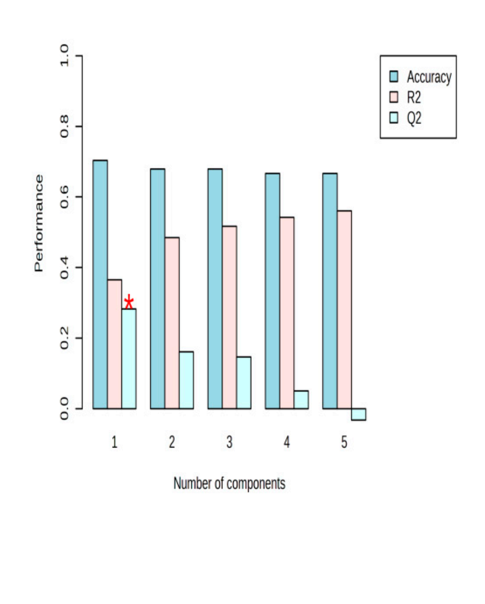

Cross Validation Graph

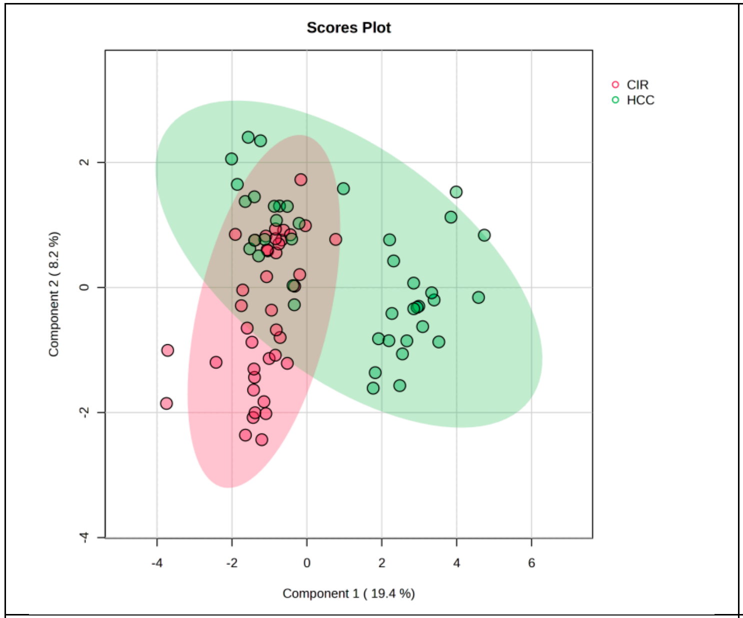

sPLSDA score plot

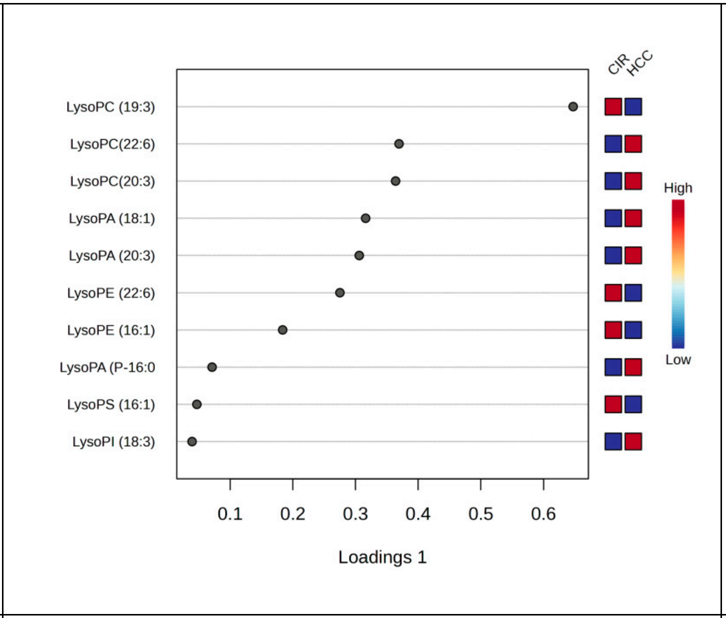

sPLSDA Loadings plot

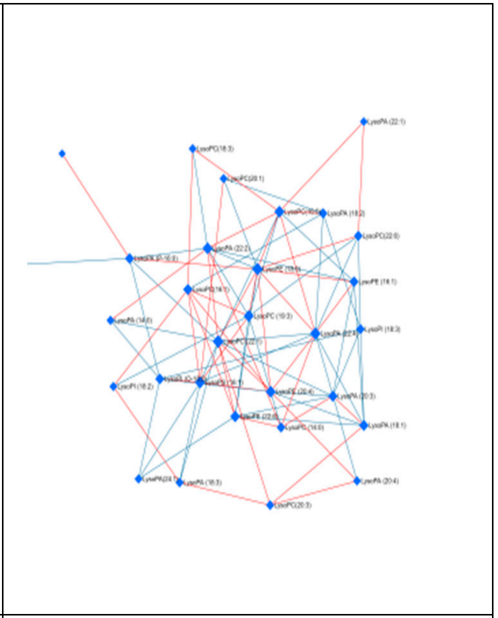

Network links

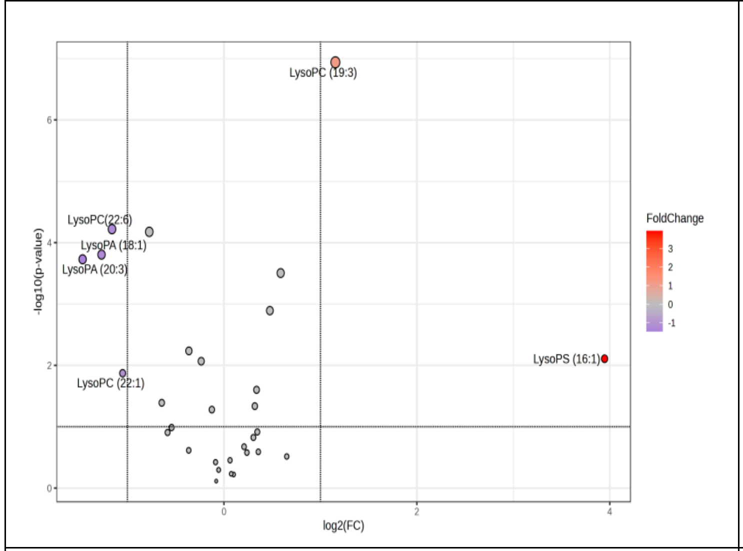

Volcano Plot

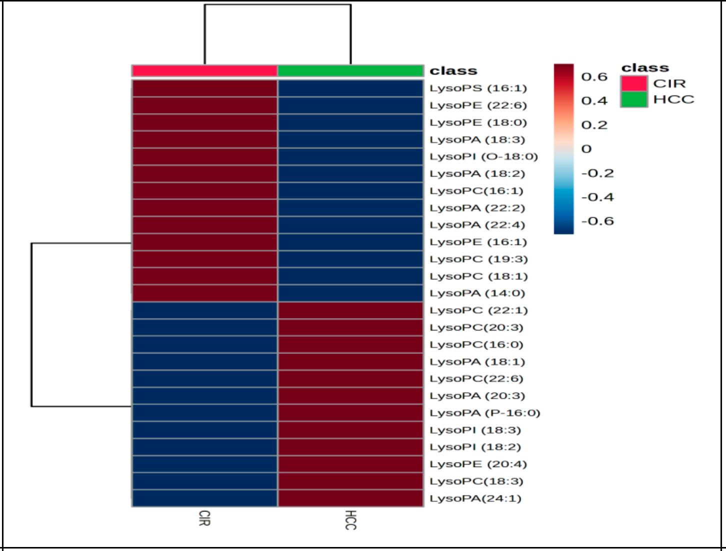

Heatmap- considering mean features per group

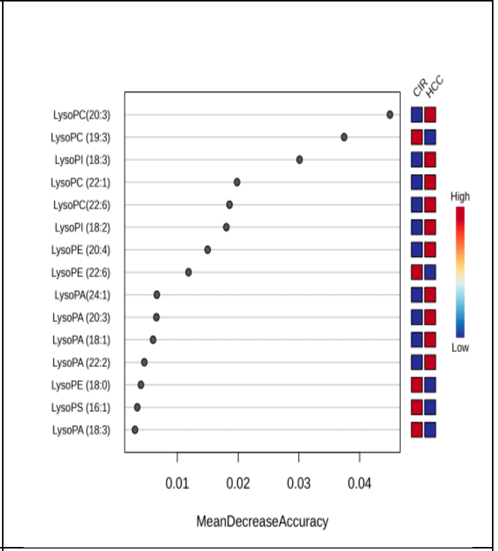

Random Forest (Rf) plot

5. Acylcarnitines

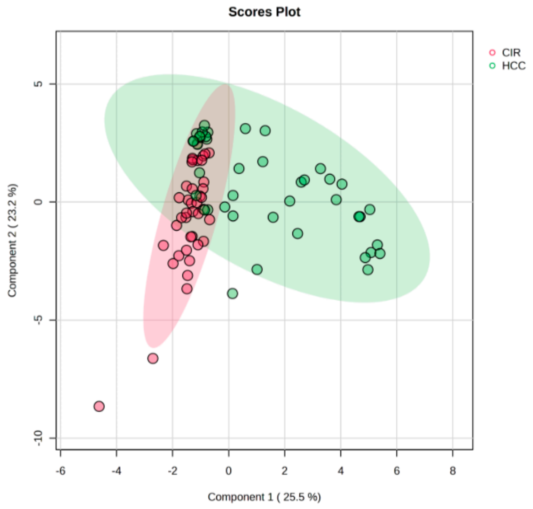

PLSDA score plot

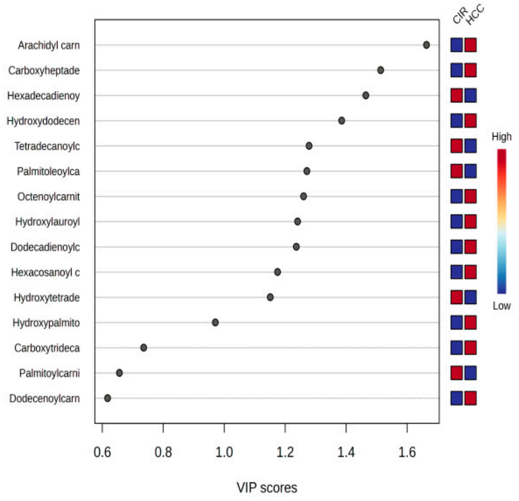

PLSDA loadings VIP score

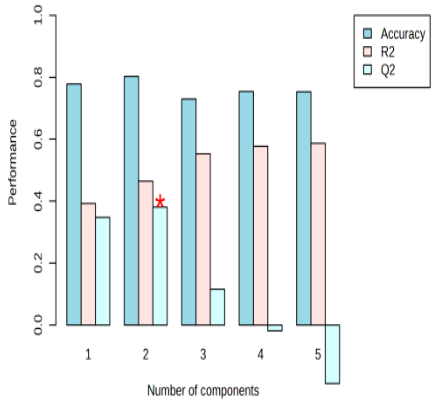

Cross Validation Graph

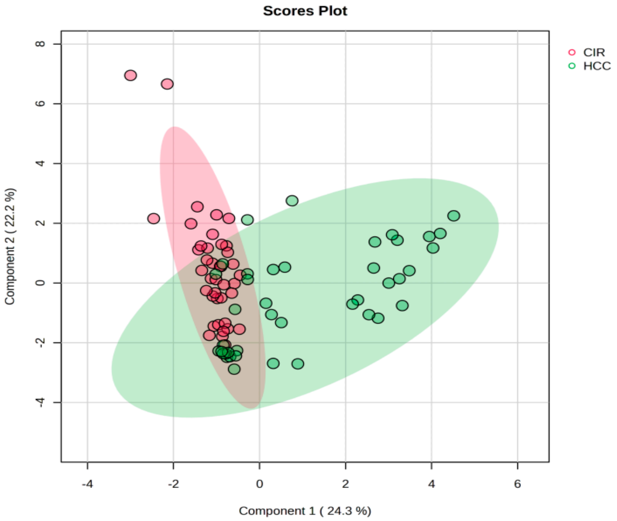

sPLSDA score plot

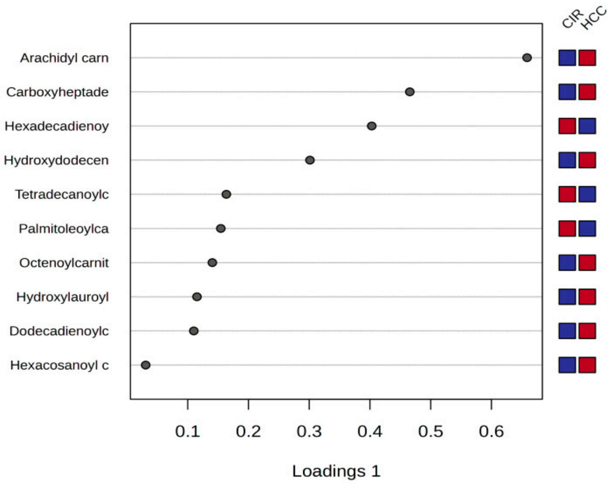

sPLSDA Loadings plot

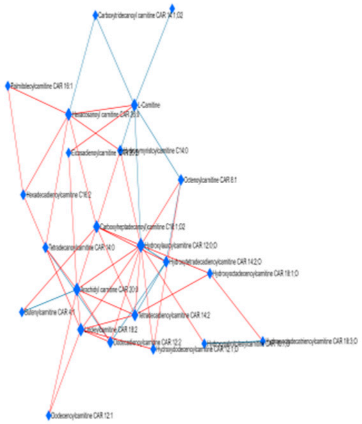

Network links

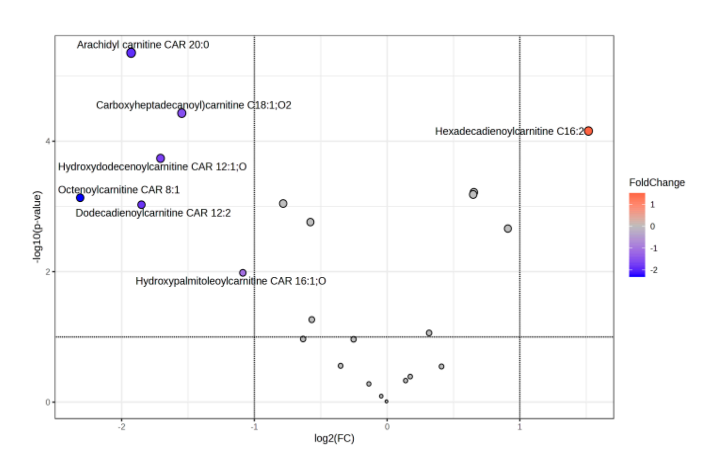

Volcano Plot

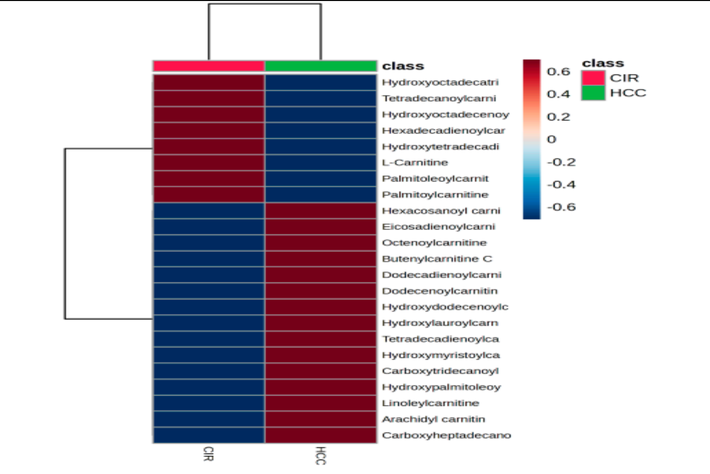

Heatmap- considering mean features per group

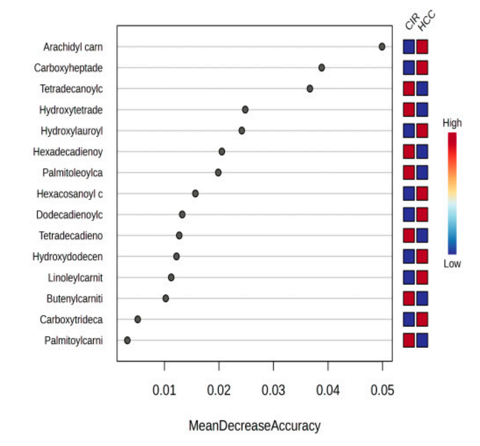

Random Forest (Rf) plot

6. MONO- and DIglycerides (MG, DG)

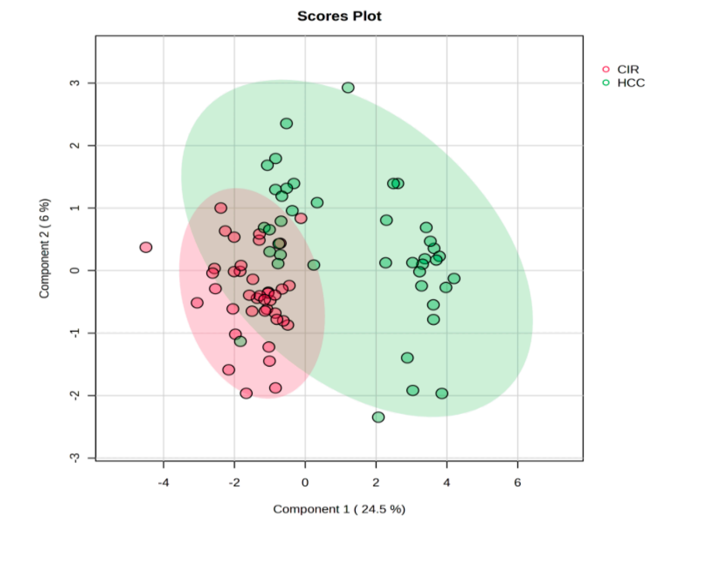

PLSDA score plot

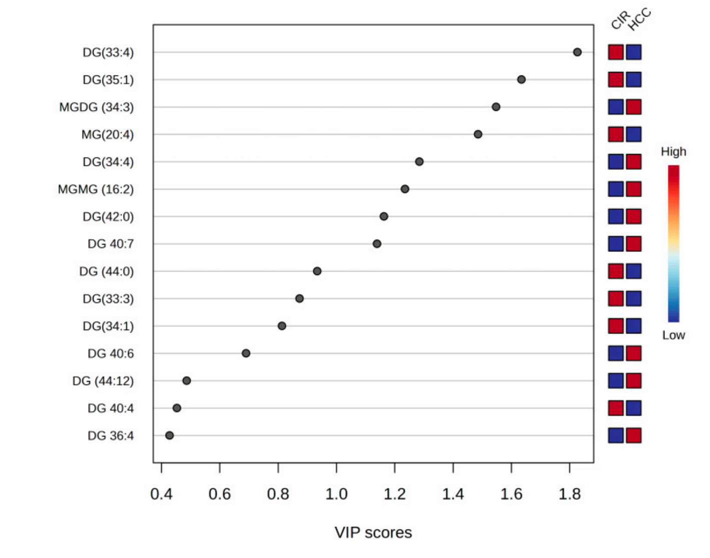

PLSDA loadings VIP score

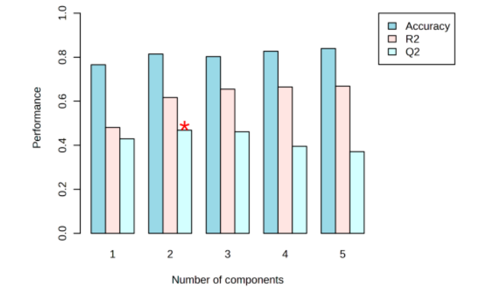

Cross Validation Graph

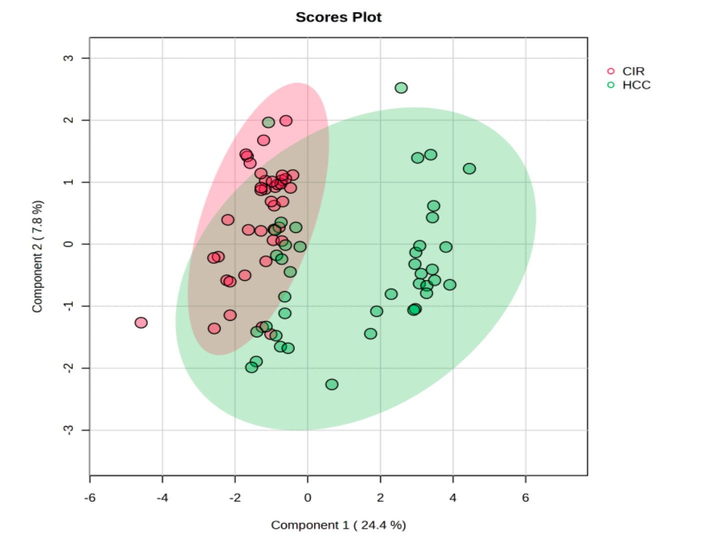

sPLSDA score plot

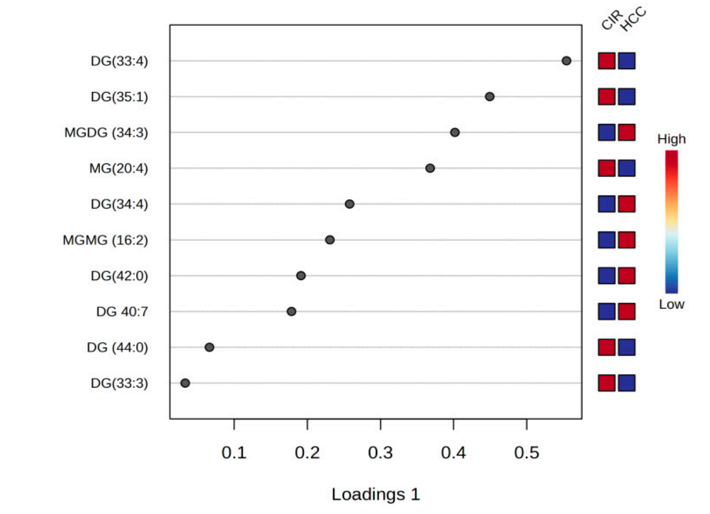

sPLSDA Loadings plot

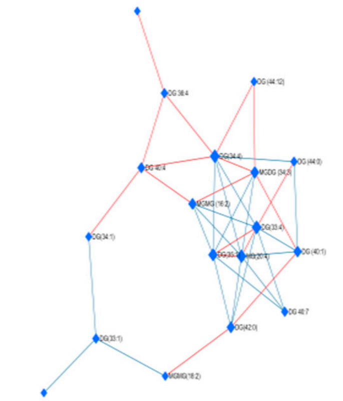

Network links

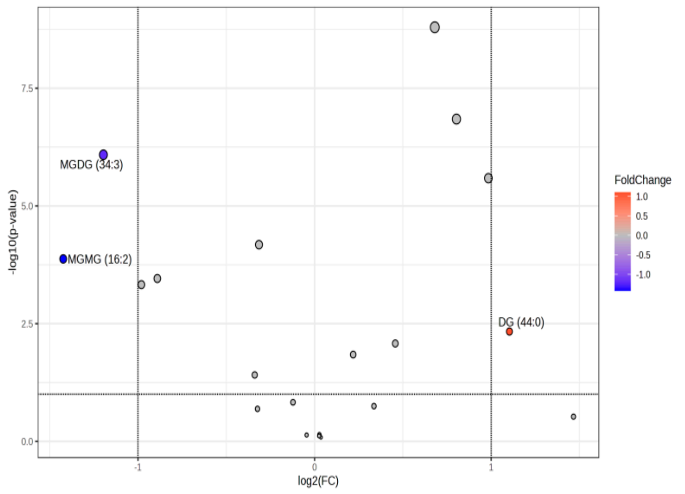

Volcano Plot

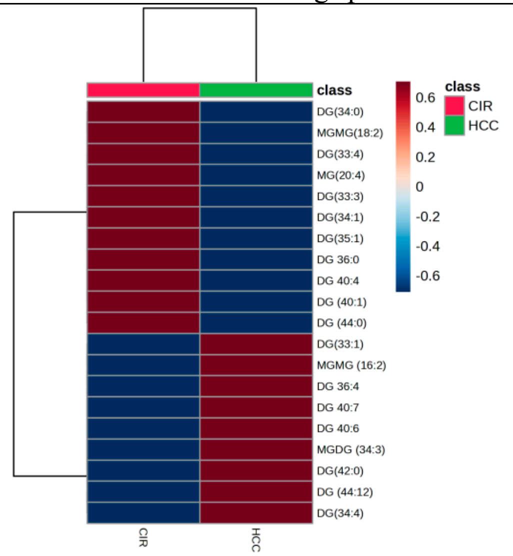

Heatmap- considering mean features per group

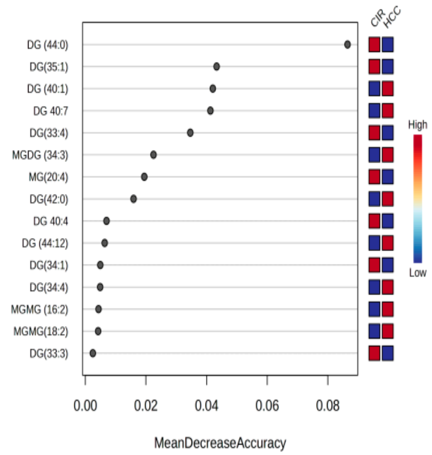

Random Forest (Rf) plot

7. Sphingolipids

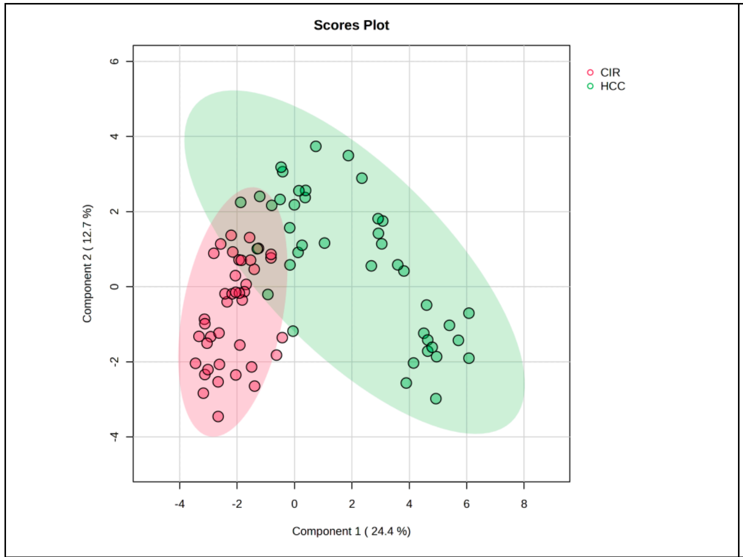

PLSDA score plot

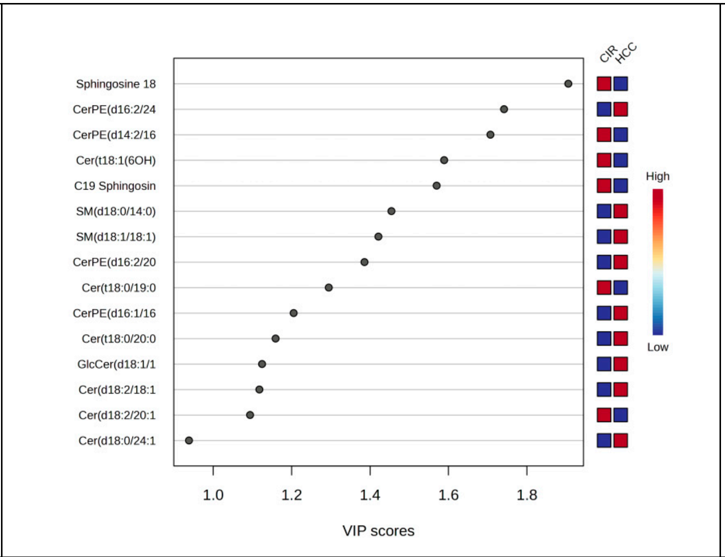

PLSDA loadings VIP score

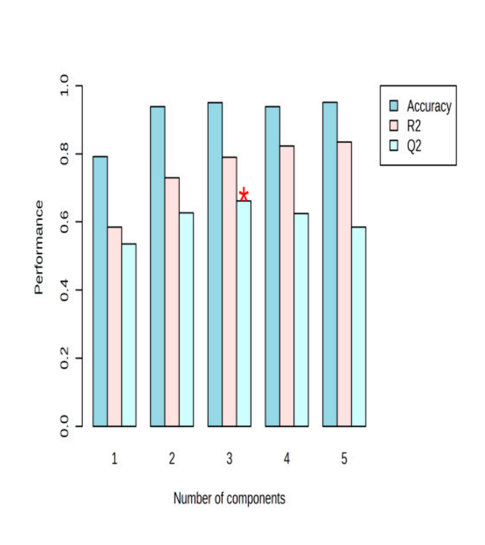

Cross Validation Graph

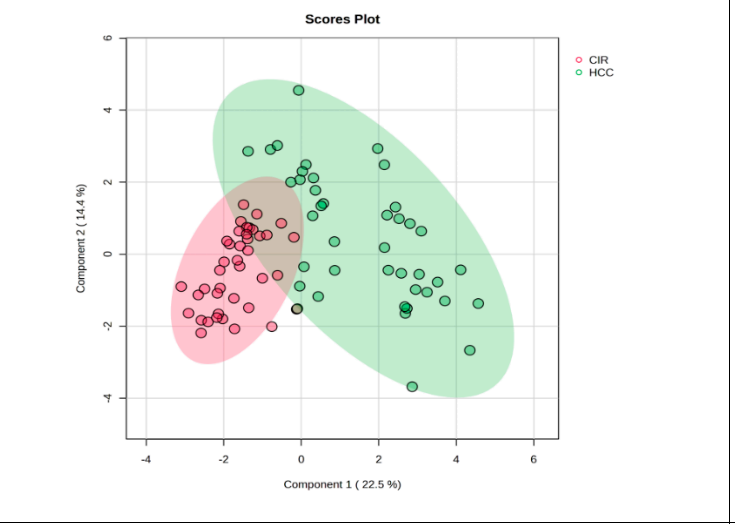

sPLSDA score plot

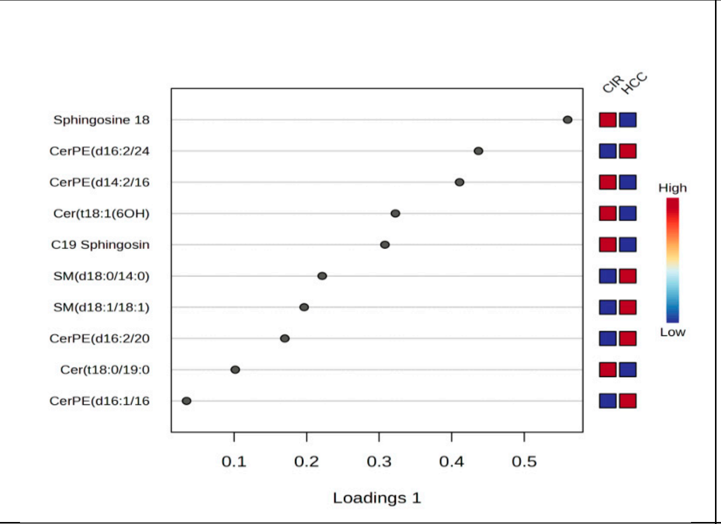

sPLSDA Loadings plot

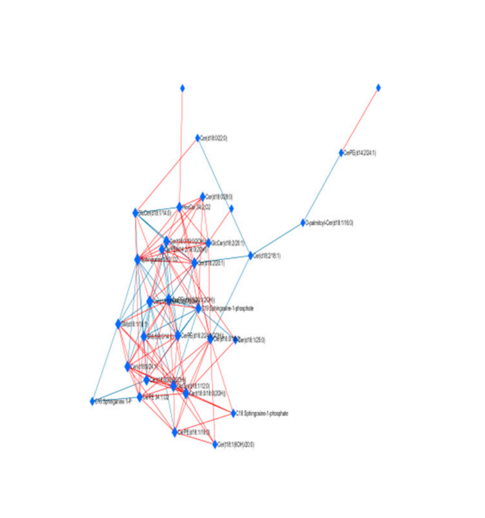

Network links

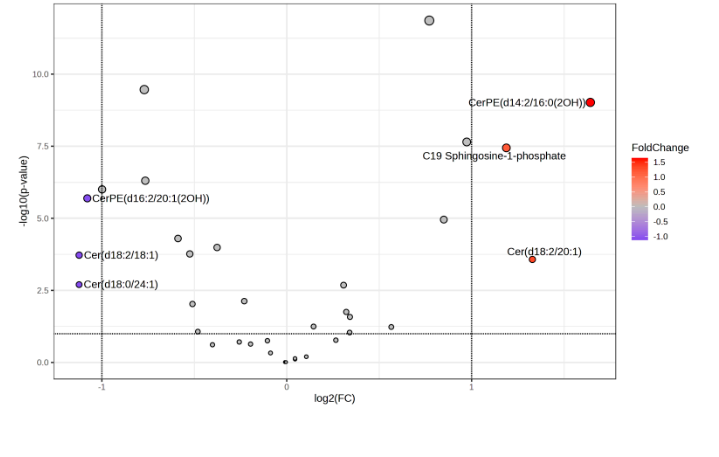

Volcano Plot

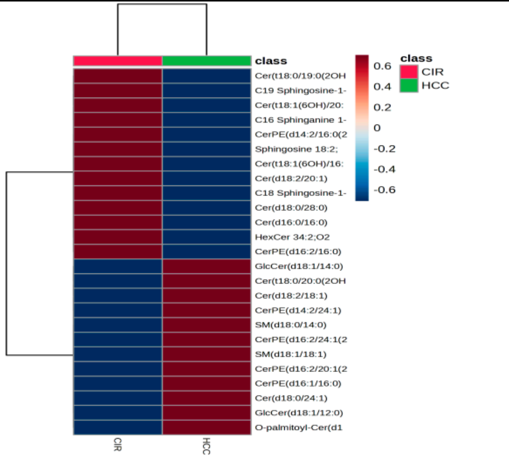

Heatmap- considering mean features per group

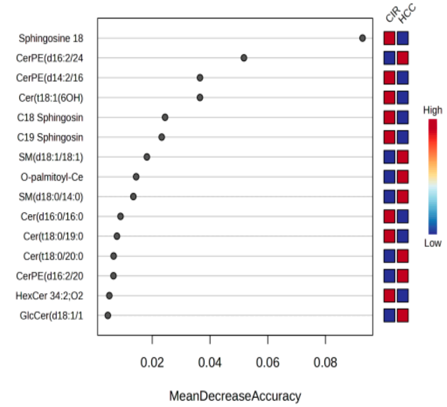

Random Forest (Rf) plot

8.Sterols

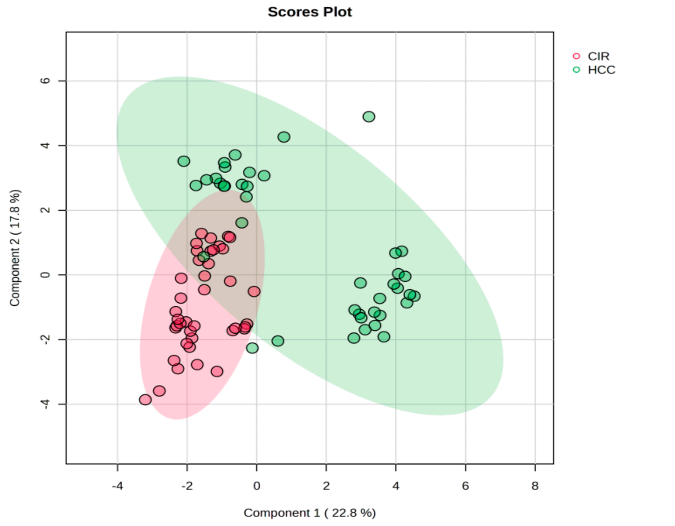

PLSDA score plot

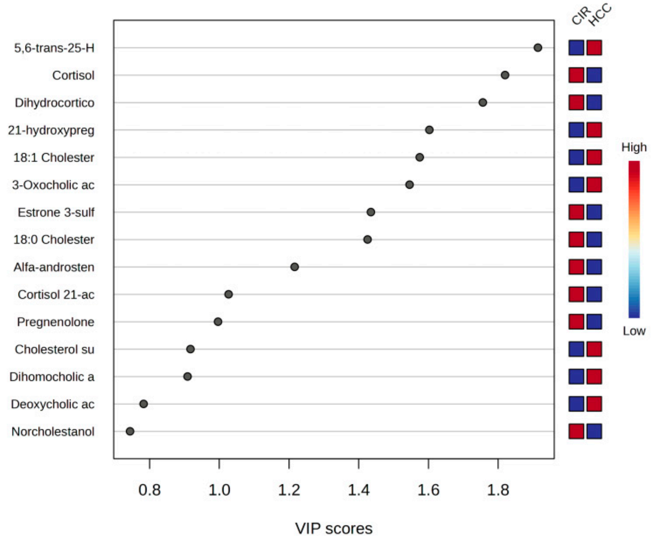

PLSDA loadings VIP score

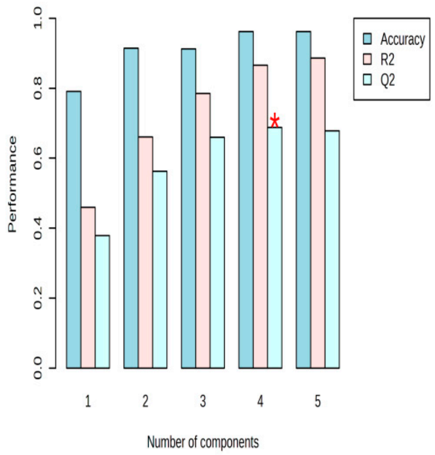

Cross Validation Graph

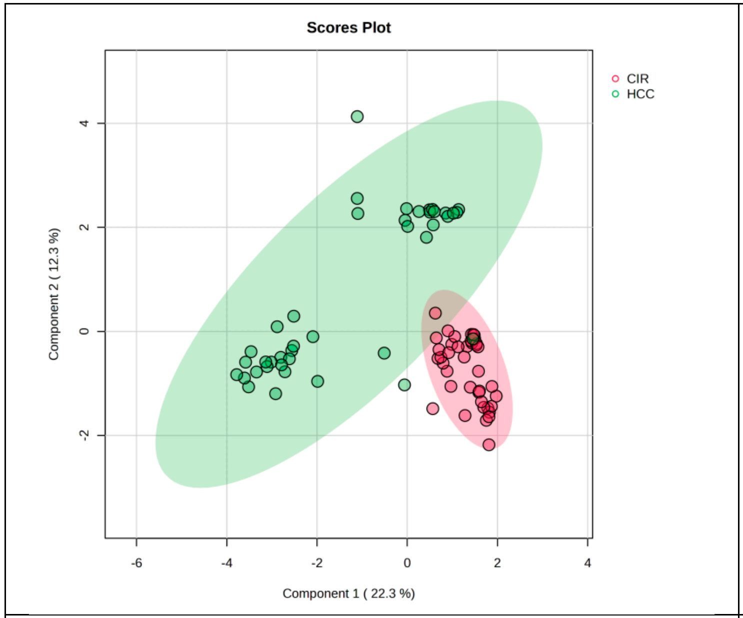

sPLSDA score plot

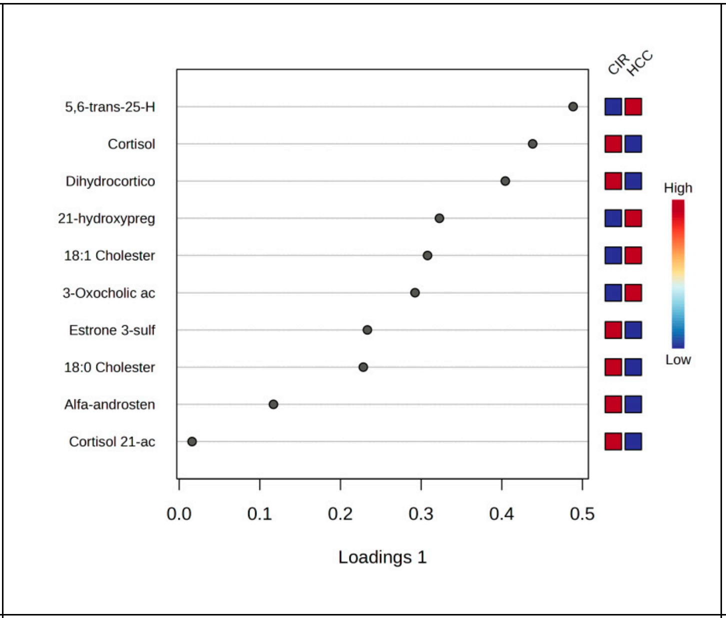

sPLSDA Loadings plot

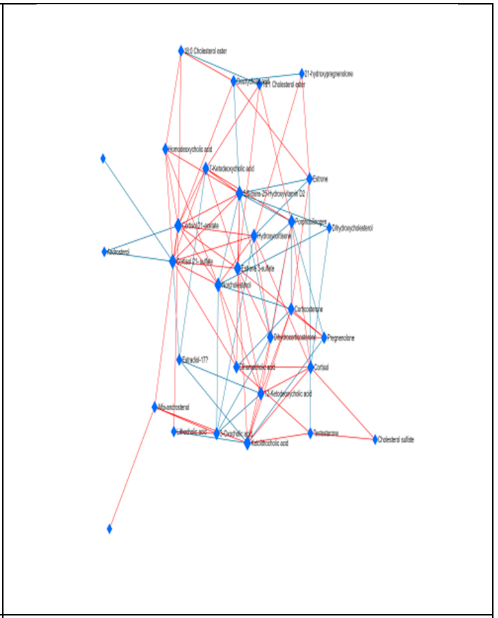

Network links

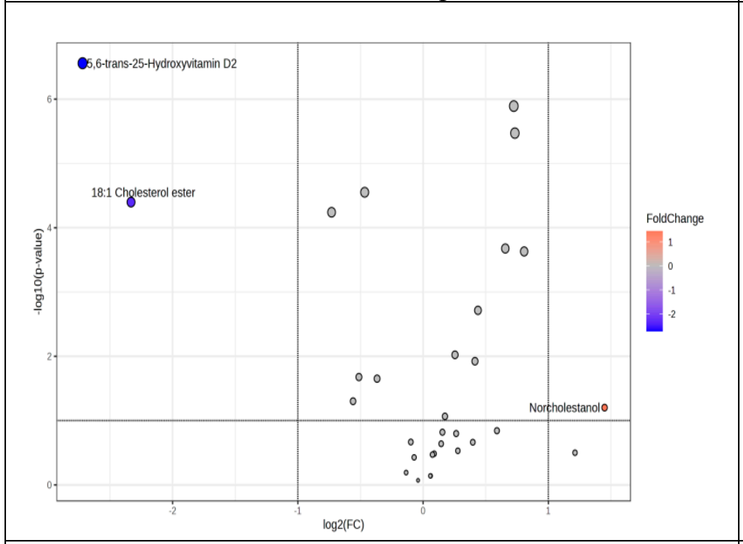

Volcano Plot

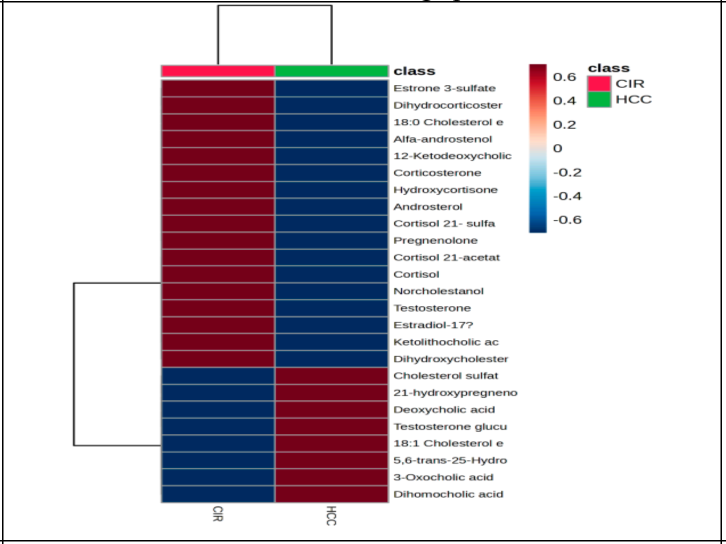

Heatmap- considering mean features per group

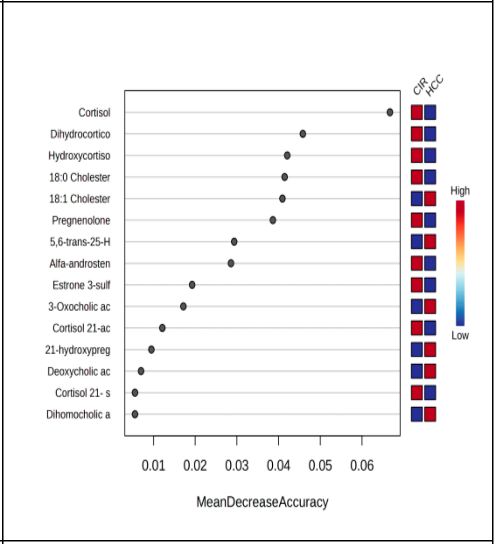

Random Forest (Rf) plot

9. Oxylipins

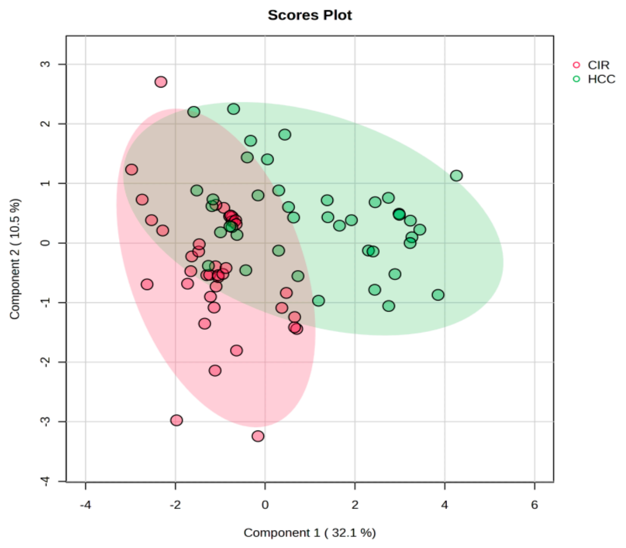

PLSDA score plot

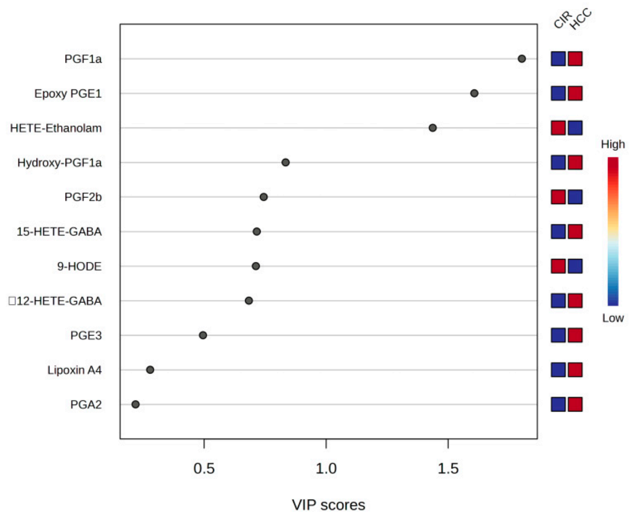

PLSDA loadings VIP score

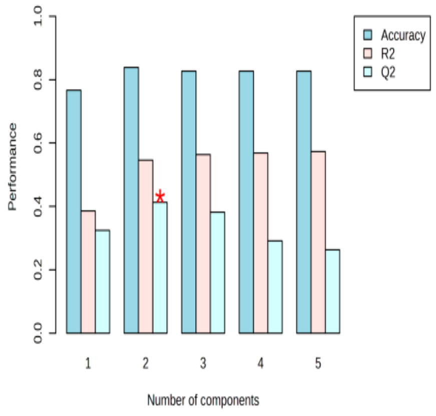

Cross Validation Graph

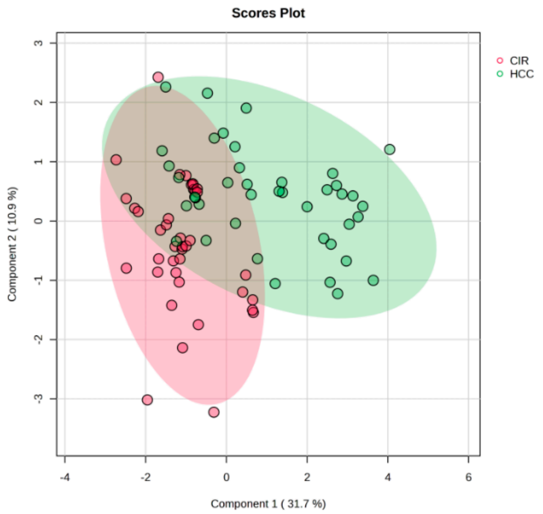

sPLSDA score plot

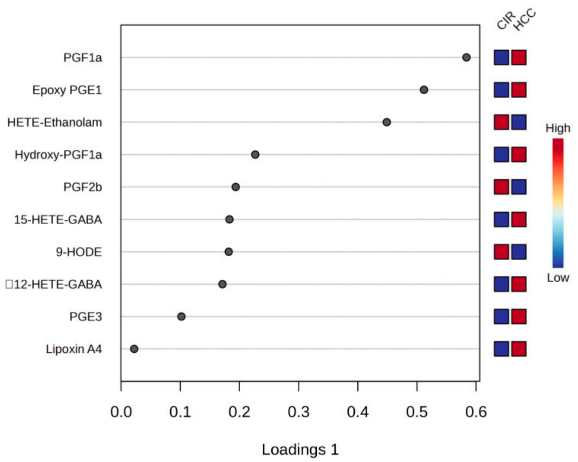

sPLSDA Loadings plot

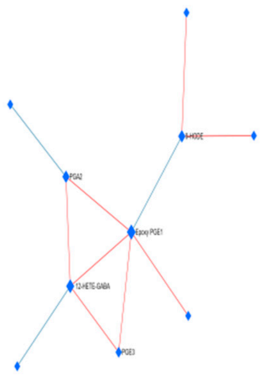

Network links

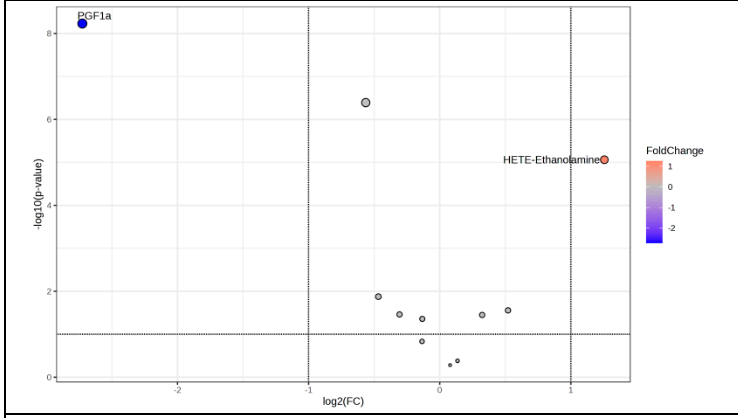

Volcano Plot

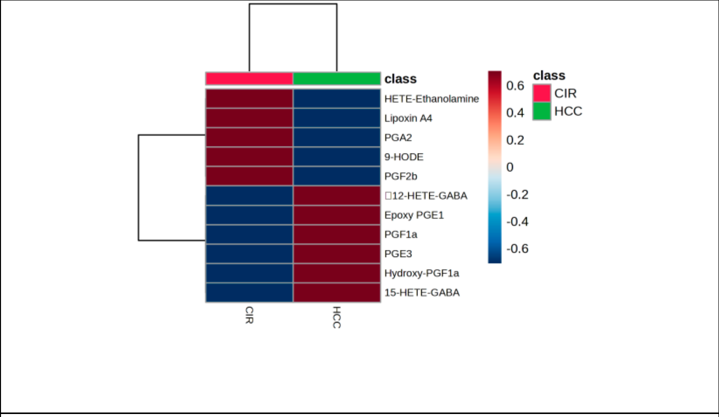

Heatmap- considering mean features per group

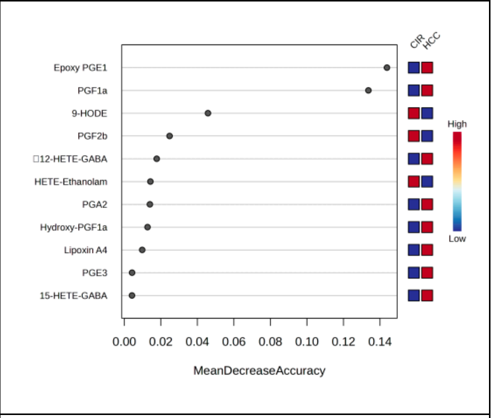

Random Forest (Rf) plot

10. Lipid Antioxidants

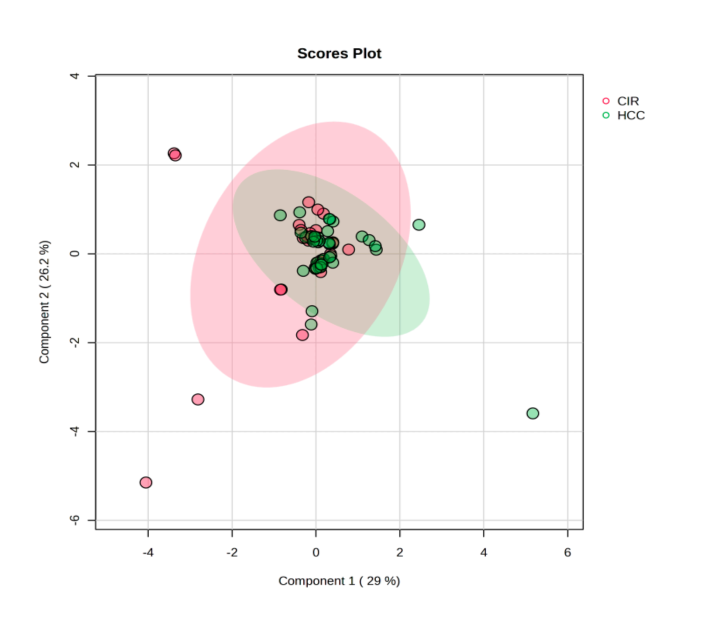

PLSDA score plot

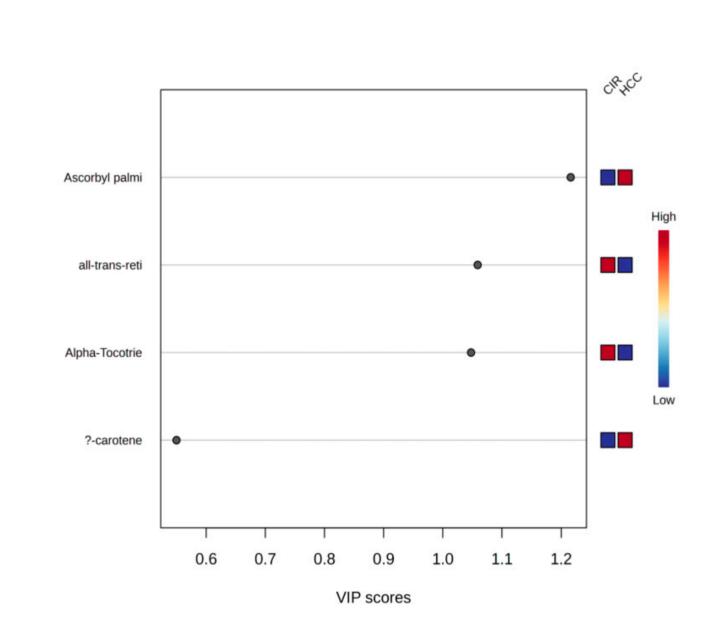

PLSDA loadings VIP score

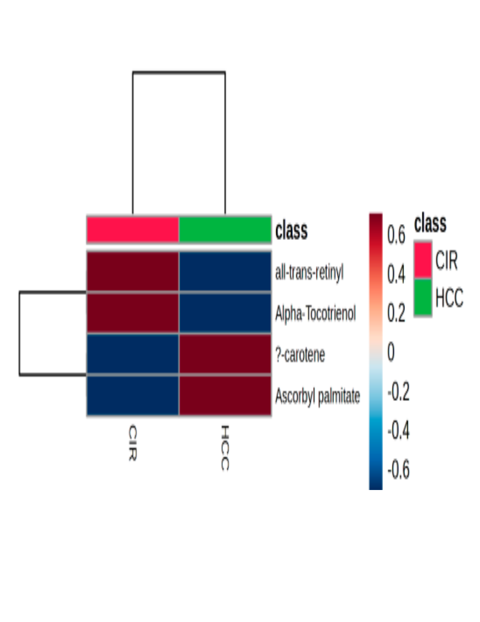

Heatmap- considering mean features per group

11. Polar molecules

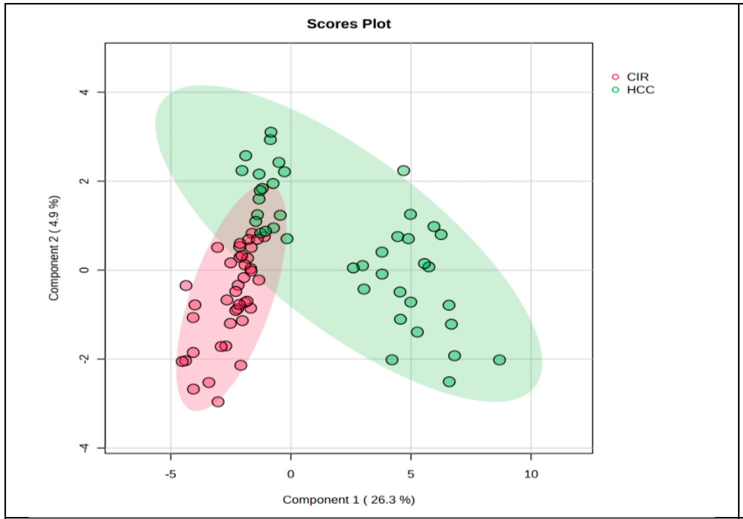

PLSDA score plot

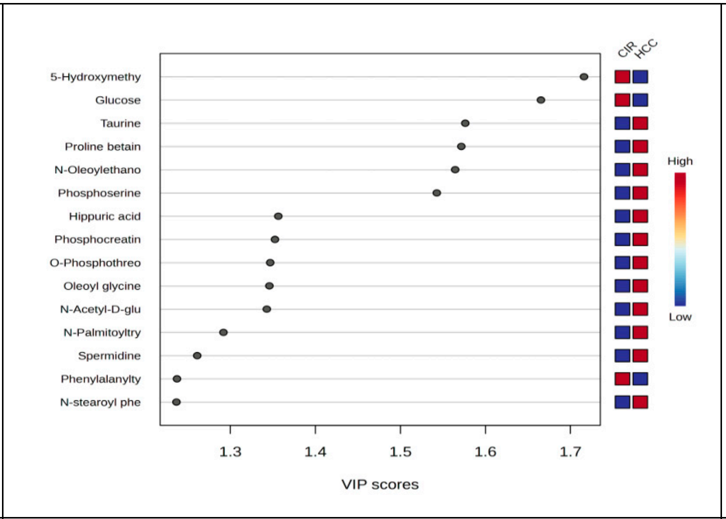

PLSDA loadings VIP score

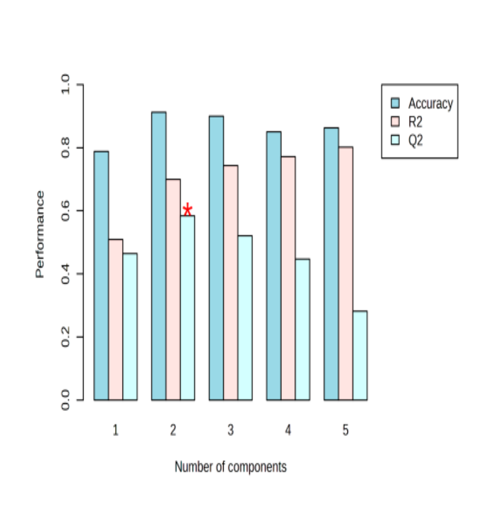

Cross Validation Graph

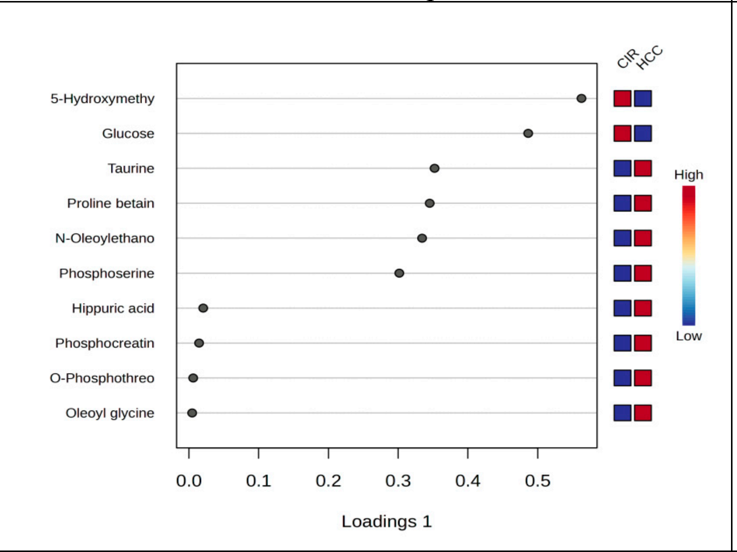

sPLSDA score plot

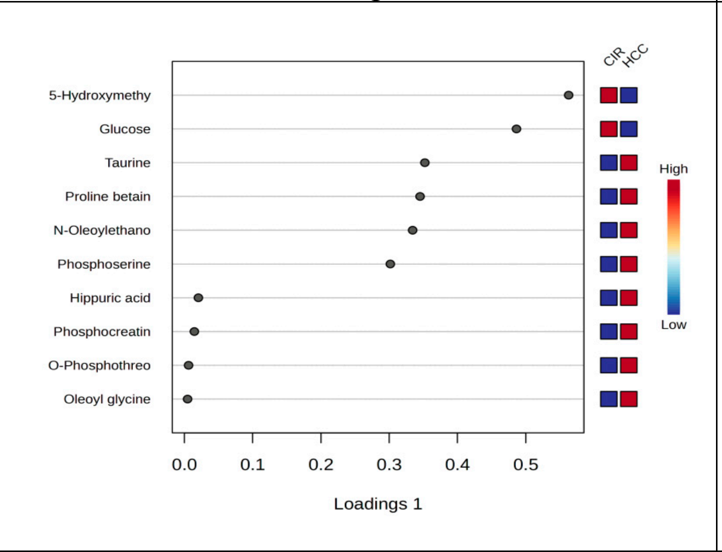

sPLSDA Loadings plot

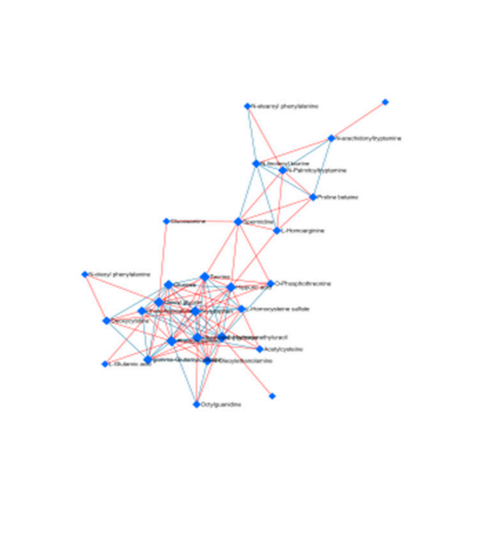

Network links

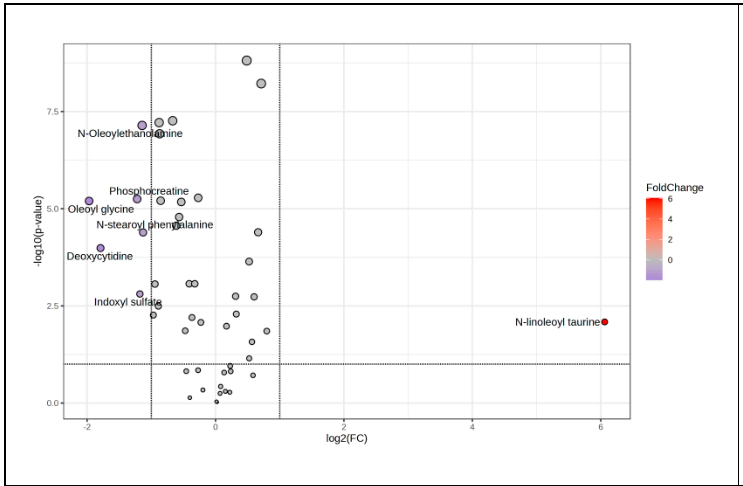

Volcano Plot

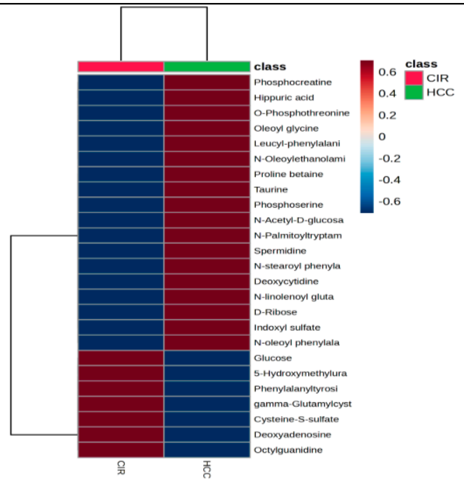

Heatmap- considering mean features per group

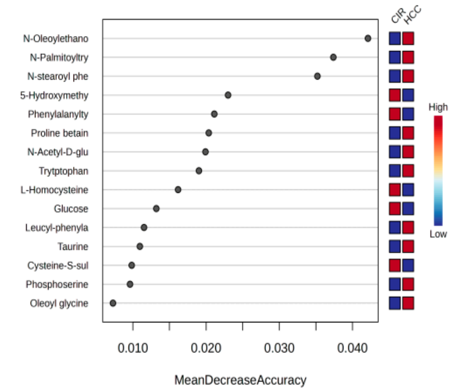

Random Forest (Rf) plot
