# Supplementary material for: Differential Metabolic Dysregulations in Hepatocellular Carcinoma and Cirrhosis: Insights into Lipidomic Signatures
Source: Biomolecules. 2025 Nov 10;15(11):1575. doi: 10.3390/biom15111575 (PMC12650657; doi:10.3390/biom15111575)
Supplement: Supplementary file 1 [file biomolecules-15-01575-s001.zip › Figure S3. The most representative molecules from each class of metabolites.pdf]

Figure S3 The most representative molecules from each class of metabolites, selected by both the semi-targeted analysis and Biomarker analysis.

A. **Free fatty acids**<br>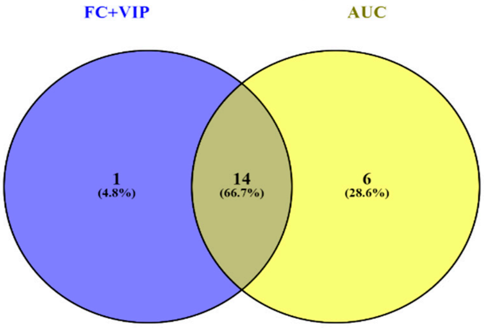 	HCC<CIR:<br>Docosapentenoic acid C22:5<br>Palmitoleic acid C16:1<br>Dodecenoic acid C12:1<br>Docosahexaenoic acid (DHA) C22:6<br>3-Hydroxysuberic acid C8:1;O3<br>Triacontatrienoic acid C30:3	**HCC>CIR**<br>Methyl-tridecanedioic acid C14:2<br>Octatriacontanoic acid C 38:0<br>Hydroxy-Eicosapentenoic acid C20:5;O<br>Tetracontahexaenoic acid C40:6<br>Stearic acid C18:0<br>10-oxo-docosanoic acid C22:1;O<br>Arachidic acid C20:0<br>Heptadecenoic acid C17:1
B. **Fatty acid derivatives**<br>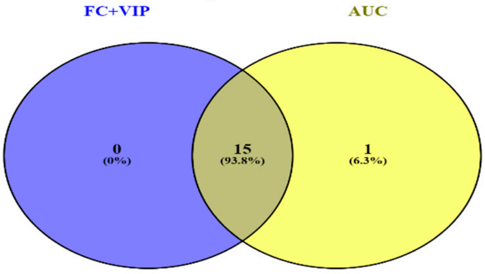 	HCC<CIR:<br>Palmitoleyl palmitoleate<br>Amino-octanoic acid<br>Stearamide<br>Myristoleyl arachidonate<br>Docosenamide<br>Palmityl palmitoleate	**HCC>CIR**<br>Linolenyl stearate<br>Stearyl stearate<br>Oleyl palmitate<br>Linoleyl arachidonate<br>Linoleyl stearate<br>Linoleyl linoleate<br>Palmitoleyl linolenate<br>Linoleyl arachidate<br>Palmitamide
C. **Glycerophospholipids**<br>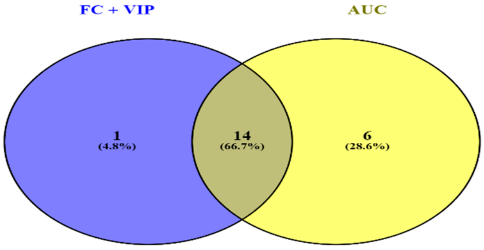 	HCC<CIR:<br>Glycerophosphocholine<br>PC (23:2; O)<br>PA 30:2<br>PA (30:4;O3)<br>PA(O-18:0/16:0)	**HCC>CIR**<br>PA 32:0<br>PA (O-36:3)<br>PC 32:1<br>PA 36:6<br>PA(P-18:0/18:2)

		PA 38:6<br>PS 34:0<br>PG O-34:4<br>PA 42:4
D. **Lysophospholipids**<br>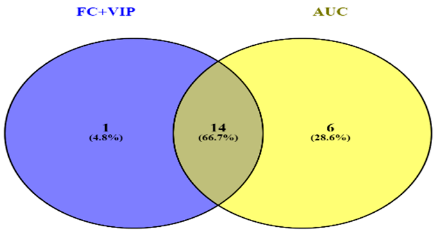 	HCC<CIR:<br>LysoPE (16:1)<br>LysoPC (19:3)<br>LysoPE (22:6)<br>LysoPE (18:0)<br>LysoPC (18:1)	**HCC>CIR**<br>LysoPC(16:0)<br>LysoPI (18:3)<br>LysoPA (P-16:0)<br>LysoPI (18:2)<br>LysoPC(20:3)<br>LysoPC (22:1)<br>LysoPC(22:6)<br>LysoPA (18:1)<br>LysoPA (20:3)
E. **Acylcarnitines**<br>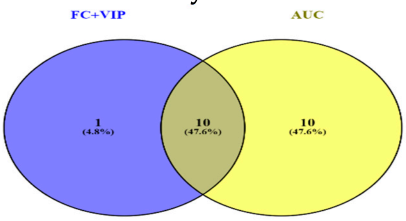 	HCC<CIR:<br>Tetradecanoylcarnitine CAR 14:0<br>Palmitoleoylcarnitine CAR 16:1	**HCC>CIR**<br>Hexacosanoyl carnitine CAR 26:0<br>Hydroxylauroylcarnitine CAR 12:0;O<br>Hydroxypalmitoleoylcarnitine CAR 16:1;O<br>Carboxyheptadecanoyl)carnitine C18:1;O2<br>Hydroxydodecenoylcarnitine CAR 12:1;O<br>Dodecadienoylcarnitine CAR 12:2<br>Arachidyl carnitine CAR 20:0<br>Octenoylcarnitine CAR 8:1
F. **Mono- and diglycerides**<br>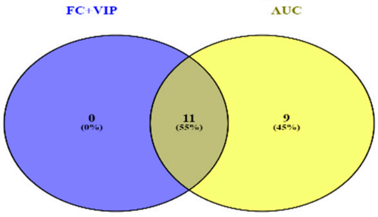 	HCC<CIR:<br>DG (44:0)<br>MG(20:4)<br>DG(35:1)<br>DG(33:4)<br>DG(33:3)	**HCC>CIR**<br>DG(34:4)<br>DG(42:0)<br>DG 40:7<br>MGDG (34:3)<br>MGMG (16:2)

	DG(34:1)	
G. **Sphingolipids**<br>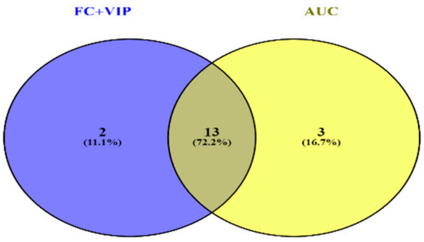 	HCC<CIR:<br>CerPE(d14:2/16:0(2OH)<br>Cer(d18:2/20:1)<br>C19 Sphingosine-1-phosphate<br>Cer(t18:1(6OH)/16:0(2OH))<br>Cer(t18:0/19:0(2OH))<br>Sphingosine 18:2; O2	**HCC>CIR**<br>Cer(t18:0/20:0(2OH))<br>GlcCer(d18:1/14:0)<br>CerPE(d16:1/16:0)<br>SM(d18:0/14:0)<br>CerPE(d16:2/24:1(2OH))<br>SM(d18:1/18:1)<br>CerPE(d16:2/20:1(2OH))
H. **Sterol lipids**<br>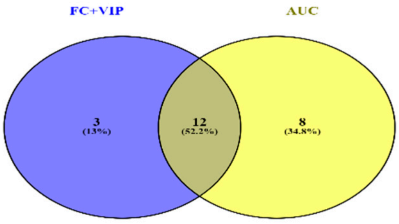 	HCC<CIR:<br>18:0 Cholesterol ester<br>Dihydrocorticosterone<br>Cortisol<br>Estrone 3-sulfate<br>Alfa-androstenol<br>Cortisol 21-acetate	HCC>CIR<br>Dihomocholic acid<br>21-hydroxypregnenolone<br>Cholesterol sulfate<br>Deoxycholic acid<br>3-Oxocholic acid<br>25-Hydroxyvitamin D2
I. **Oxilipins and antioxidants**<br>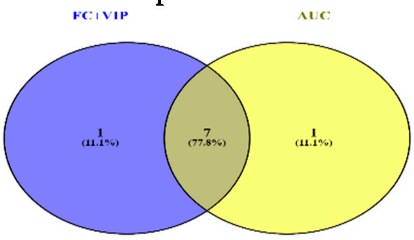 	HCC<CIR:<br>HETE-Ethanolamine<br>Alpha-Tocotrienol<br>all-trans-retinyl oleate	**HCC>CIR**<br>Ascorbyl palmitate<br>Hydroxy-PGF1a<br>Epoxy PGE1<br>PGF1a
J. **Polar metabolites**	HCC<CIR:<br>Glucose	**HCC>CIR**<br>N-Acetyl-D-glucosamine

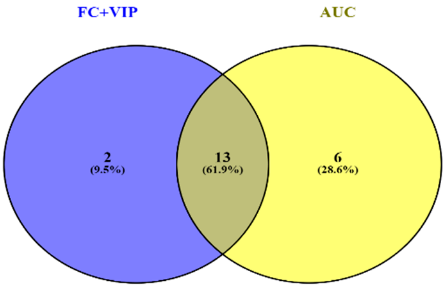	5-Hydroxymethyluracil<br><br>Hippuric acid	Spermidine<br><br>Taurine<br><br>O-Phosphothreonine<br><br>Phosphoserine<br><br>Proline betaine<br><br>N-stearoyl phenylalanine<br><br>N-Oleoylethanolamine<br><br>N-Palmitoyltryptamine<br><br>Oleoyl glycine
