# Supplementary material for: Differential Metabolic Dysregulations in Hepatocellular Carcinoma and Cirrhosis: Insights into Lipidomic Signatures
Source: Biomolecules. 2025 Nov 10;15(11):1575. doi: 10.3390/biom15111575 (PMC12650657; doi:10.3390/biom15111575)
Supplement: Supplementary file 1 [file biomolecules-15-01575-s001.zip › Table S1. Identification of different classes of metabolites.pdf]

Table S1. Identification of 11 classes of metabolites, according to international databases: HMDB (hmdb.ca) and or Lipidmaps (lipidmaps.org). The m/z values correspond to the precursor ion (adduct M+H+] and identified by comparison with the average isotopic mass and a mass tolerance of 0.05 Da, according to HMDB and LipidMaps databases (IDs included).

m/z	Free fatty acids (n=53)	
149.0845	Mevalonic acid C6:0	LMFA01050352
155.0327	Nonadienoic acid C9:2	LMFA01030448
161.0827	2-hydroxy caprylic acid C8:0; O	LMFA01050020
167.1298	Decatrienoic acid C10:3	LMFA01030962
169.0971	Decadienoic acid C10:2	LMFA01030106
171.1179	Decenoic acid C10:1	LMFA01030198
173.1402	Capric acid C10:0	LMFA01010010
183.1239	Undecadienoic acid C11:2	LMFA01030222
191.1488	3-Hydroxysuberic acid C8:1;O3	LMFA01170093
193.1114	Dodecadiynoic acid C12:4	LMFA01030464
199.1284	Dodecenoic acid C12:1	LMFA01030227
207.1496	Lipoic acid C8:8; 2S	LMFA01130001
213.1311	Traumatin C12:2;O	LMFA01060167
217.1401	Hydroxylauric acid C12:0;O	LMFA01050363
229.1247	Myristic acid C14:0	LMFA01010014<br>HMDB0000806
249.1877	Hexadecatetraenoic acid C16:4	LMFA01030280
253.1584	Hexadecadienoic acid C16:2	LMFA01030109
255.1813	Palmitoleic acid C16:1	LMFA01030055<br>HMDB0003229
259.1474	Methyl-tridecanedioic acid C14:2	LMFA01170015
267.2165	Heptadecynoic acid C17:2	LMFA01030483
269.1894	Heptadecenoic acid C17:1	LMFA01030284
277.1967	Stearidonic acid C18:4	LMFA01030357
279.2121	alpha-Linolenic acid C18:3	LMFA01030152<br>HMDB0003073
285.2081	Stearic acid C18:0	LMFA01010018<br>HMDB0000827
295.2051	Hydroxy linolenic acid C18:3;O	LMFA02000239
297.1184	Eicosatetraynoic acid C20:8	LMFA01030690
301.1212	Eicosatetraynoic acid C20:6	LMFA01030692<br>HMDB0002925
307.2366	Eicosatrienoic acid FA 20:3	LMFA01030157<br>HMDB0002231
311.2334	Eicosenoic acid FA 20:1	LMFA01030082

		HMDB0002212
313.2105	Arachidic acid C20:0	LMFA01010020
319.2627	Hydroxy-Eicosapentenoic acid C20:5;O	LMFA01050603
321.2171	Hydroxy-Eicosatetraenoic acid C20:4;O	LMFA01030719
327.1995	Hydroxy-eicosenoic acid C20:1;O	LMFA01050256
329.2041	Docosahexaenoic acid (DHA) C22:6	LMFA01030185 HMDB0002183
331.1863	Docosapentenoic acid C22:5	LMFA01030181
333.2363	Docosatetraenoic acid C22:4	LMFA01030177
336.2925	Docosatrienoic acid C22:3	LMFA04000087
337.2118	Docosadienoic acid acid C 22:2	LMFA01170127
343.2717	Eicosanedioic acid C 20:1; O2	LMFA01170035
355.3458	10-oxo-docosanoic acid C22:1;O	LMFA01060139
359.2081	Tetracosapentaenoic acid C24:5	LMFA01030820
369.3581	Tetracosanoic (lignoceric) acid C24:0	LMFA01010024
371.2473	Hydroxy-tricosanoic acid	LMFA01050212
441.2965	Triacontahexaenoic acid C 30:6	LMFA01030840
443.2221	Triacontapentaenoic acid C 30:5	LMFA01030833
445.2792	Triacontatetraenoic acid C 30:3	LMFA01030826
447.2602	Triacontatrienoic acid C30:3	LMFA01030872
449.2797	FA 30:2	LMFA01020366
449.3638	Triacontadienoic acid C30:2	LMFA01030879
451.3296	Triacontenoic acid C30:1	LMFA01030097
493.3864	FA 33:1	LMFA07040087
565.2916	Octatriacontanoic acid C 38:0	LMFA01010038
581.4321	Tetracontahexaenoic acid C40:6	LMFA01031241

**m/z**	**Fatty acid derivatives (n=17)**	
159.9651	Amino-octanoic acid	LMFA01100020
256.2820	Palmitamide	LMFA08010009
284.3125	Stearamide	LMFA08010003
338.3189	Docosenamide	LMFA08010028
356.3231	Eicosanoyl-Ethanolamine C20:0	LMFA08040038
453.3094	Myristyl palmitate	LMFA07010002
477.2508	Palmitoleyl palmitoleate	LMFA07010113
479.2790	Palmityl palmitoleate	LMFA07010020
499.3314	Myristoleyl arachidonate	LMFA07010119
501.3406	Palmitoleyl linolenate	LMFA07010120
507.2873	Oleyl palmitate	LMFA07010133

529.3627	Linoleyl linoleate	LMFA07010146
531.3477	Linolenyl stearate	LMFA07010148
533.3127	Linoleyl stearate	LMFA07010152
537.2990	Stearyl stearate	LMFA07010054
553.4020	Linoleyl arachidonate	LMFA07010160
561.3585	Linoleyl arachidate	LMFA07010164

m/z	Glycerophospholipids (n=36)	
258.2611	Glycerophosphocholine	HMDB0000086
485.3436	PG 16:0	LMGP04050008
509.3808	PG 18:2	LMGP04050014
521.2643	PA 22:2;O	LMGP20070019
523.3129	PA 23:0)	HMDB0115486
605.3795	PA(26:4;O3)	LMGP20070030
617.2816	PA 30:2	LMGP10010051
620.3899	PC (23:2; O)	LMGP20010028
639.3582	PA 32:5	LMGP10010061
641.3486	PA 32:4	LMGP10010060
649.4018	PA 32:0	LMGP10010013<br>HMDB0000674
658.4619	PE 30:3	LMGP02010710
661.3087	PA (30:4;O3)	LMGP20070034
663.4132	PA(O-18:0/16:0)	LMGP10020075
665.3864	PA 34:6	LMGP10010130
669.3689	PA 34:4	LMGP10010065<br>HMDB0114785
682.5273	PE 32:5	LMGP02010376
683.3811	PI (22:2;O)	LMGP20050019
685.3431	PA(P-18:0/18:2)	LMGP10030040
685.4529	PA (O-36:3)	LMGP10020030
688.4706	PE 32:2	LMGP02010108
693.4258	PA 36:6	LMGP10010378<br>HMDB0114792
705.3302	PA (O-38:7)	LMGP10030093
705.4526	PA 36:0	LMGP10010028
716.5002	PC(P-16:0/16:1))	LMGP01030026
719.3355	PG 32:2	LMGP04010060
721.4558	PA 38:6	LMGP10010038
729.3659	PG O-34:4	LMGP04020010

732.4949	PC 32:1	LMGP01010566<br>HMDB0007969
735.4778	PG O-34:1	LMGP04020044
749.3520	PG 34:1	LMGP04010002
750.5099	PE (O-38:6)	LMGP02020020
754.5682	PE (O-38:4)	LMGP02020092
760.5254	PC 34:1	LMSP02010178
765.4801	PS 34:0	LMGP03010888<br>HMDB0012376
781.4709	PA 42:4	LMGP10010805

m/z	Lyso Phospholipids n=33)	
383.1772	LysoPA (14:0)	HMDB0062321
395.2127	LysoPA (P-16:0)	HMDB0011154
433.2288	LysoPA (18:3)	LMGP10050023
435.3363	LysoPA (18:2)	LMGP10050017
437.3274	LysoPA (18:1)	LMGP10050008
452.3622	LysoPE (16:1)	LMGP02050010
459.4699	LysoPA (20:4)	LMGP10050013
461.2562	LysoPA (20:3)	LMGP10050028
468.3565	LysoPC (14:0)	LMGP01050012
470.2415	LysoPS (14:0)	LMGP03050009
482.3704	LysoPE (18:0)	LMGP02050001
487.3248	LysoPA (22:4)	LMGP10050020
491.2613	LysoPA (22:2)	LMGP10050030
493.2751	LysoPA (22:1)	LMGP10050029<br>HMDB0114750
494.5327	LysoPC(16:1)	LMGP01050021<br>HMDB0010383
496.2877	LysoPS (16:1)	LMGP03050010
496.3850	LysoPC(16:0)	LMGP01050018<br>HMDB0010382
502.6465	LysoPE (20:4)	LMGP02050009
511.2020	LysoPG (18:1)	LMGP04050006
511.3582	LysoPG (18:1)	LMGP04050006
518.1193	LysoPC(18:3)	LMGP01050038<br>HMDB0010387
521.3344	LysoPA(24:1)	HMDB0114757
522.5693	LysoPC (18:1)	LMGP01050029<br>HMDB0002815
526.4009	LysoPE (22:6)	LMGP02050013

532.3435	LysoPC (19:3)	LMGP01050003
546.4505	LysoPC(20:3)	LMGP01050133 HMDB0010393
550.5915	LysoPC(20:1)	LMGP01050047
568.4321	LysoPC(22:6)	LMGP01050056 HMDB0010402
573.2664	LysoPI (16:0)	LMGP06050002
578.4322	LysoPC (22:1)	LMGP01050134
587.2802	LysoPI (O-18:0)	LMGP06060002
595.3004	LysoPI (18:3)	LMGP06050016
597.2971	LysoPI (18:2)	LMGP06050010

m/z	Acyl carnitines (n=25)	
162.0379	L-Carnitine	HMDB0000062
216.2162	Propenoylcarnitine	HMDB0013124
230.2325	Butenylcarnitine CAR 4:1	HMDB0013126
286.2906	Octenoylcarnitine CAR 8:1	LMFA07070014 HMDB0013324
290.2639	Adipoylcarnitine CAR 6:1;O2	LMFA07070087
340.2336	Dodecadienoylcarnitine CAR 12:2	LMFA07070124
342.3522	Dodecenoylcarnitine CAR 12:1	LMFA07070115 HMDB0013326
358.3477	Hydroxydodecenoylcarnitine CAR 12:1;O	LMFA07070024
360.3375	Hydroxylauroylcarnitine CAR 12:0;O	HMDB0013164
368.4010	Tetradecadienoylcarnitine CAR 14:2	LMFA07070020
372.3210	Tetradecanoylcarnitine CAR 14:0	LMFA07070107 HMDB0005066
384.1681	Hydroxytetradecadienoylcarnitine CAR 14:2;O	LMFA07070019
388.3763	Hydroxymyristoylcarnitine C14:0	LMFA07070033
396.4302	Hexadecadienoylcarnitine C16:2	HMDB0240757
398.3344	Palmitoleoylcarnitine CAR 16:1	LMFA07070097 HMDB0006317
400.3499	Palmitoylcarnitine	HMDB0000222
402.3815	Carboxytridecanoyl carnitine CAR 14:1;O2	LMFA07070084
414.4193	Hydroxypalmitoleoylcarnitine CAR 16:1;O	LMFA07070044
424.3344	Linoleylcarnitine CAR 18:2	LMFA07070092
438.3259	Hydroxyoctadecatrienoylcarnitine CAR 18:3;O	LMFA07070027

442.3655	Hydroxyoctadecenoylcarnitine CAR 18:1;O	LMFA07070025
452.4949	Eicosadienoylcarnitine CAR 20:2	LMFA07070011
456.2183	Arachidyl carnitine CAR 20:0	LMFA07070052 HMDB0006460
458.6888	Carboxyheptadecanoyl)carnitine C18:1;O2	LMFA07070085
540.4090	Hexacosanoyl carnitine CAR 26:0	LMFA07070069

**m/z**	**Mono-, di- and triglycerols (n=22)**	
379.2681	MG(20:4)	HMDB0011578
489.3286	MGMG (16:2)	LMGL04010008
513.2037	DG(28:0)	LMGL02010321
517.3342	MGMG(18:2)	LMGL04010010
575.3719	DG(33:4)	LMGL02010368
577.3358	DG(33:3)	LMGL02010019
581.3243	DG(33:1)	LMGL02010013
589.3852	DG(34:4)	LMGL02010028 HMDB0007025
595.3371	DG(34:1)	LMGL02010004 HMDB0007021
597.4253	DG(34:0)	LMGL02010003 HMDB0007020
609.3146	DG(35:1)	LMGL02010029
617.4101	DG 36:4	LMGL02010063
625.4789	DG 36:0	LMGL02010002
667.5154	DG 40:7	LMGL02010197
669.4772	DG 40:6	LMGL02010186
673.4108	DG 40:4	LMGL02010163
675.3198	DG 40:3	LMGL02010151
679.4573	DG (40:1)	LMGL02010129
709.4060	DG(42:0)	LMGL02010200
713.3923	DG (44:12)	LMGL02010307
737.4811	DG (44:0)	LMGL02010258
753.4235	MGDG (34:3)	LMGL05010056

**m/z**	**Sphingolipids (n=37)**	
298.3292	Sphingosine 18:2; O2	LMSP01080010
354.3855	C16 Sphinganine 1-P	LMSP01050006
380.3119	C18 Sphingosine-1-phosphate	HMDB0000277

394.3223	C19 Sphingosine-1-phosphate	LMSP01050004
512.3787	Cer(d16:0/16:0)	LMSP02020068
556.4008	Cer(t18:1(6OH)/14:0(2OH))	LMSP02010195
562.3407	Cer(d18:2/18:1)	LMSP02010026
570.4160	Cer(t18:1(6OH)/16:0(2OH))	LMSP02030080<br>HMDB0010402
590.2267	CerP(d18:1/14:0)	LMSP02050013
590.3339	Cer(d18:2/20:1)	LMSP02010027
600.4239	Cer(t18:0/18:0(2OH))	LMSP02030016
610.5345	Cer(t18:1(6OH)/20:0)	LMSP02010144
614.4391	Cer(t18:0/19:0(2OH))	LMSP02030065
619.4381	CerPE(d14:2/16:0(2OH))	LMSP03020066
623.4862	Cer (d18:1/22:0)	LMSP02010008
625.3454	Cer(d18:0/22:0)	LMSP02030017
628.4536	Cer(t18:0/20:0(2OH))	LMSP02030017
631.2975	CerPE(d16:2/16:0)	LMSP03020047
633.4093	CerPE(d16:1/16:0)	LMSP03020019
636.5482	Cer(d16:2/24:0(2OH))	LMSP02010093
638.5607	Cer(t18:1(6OH)/22:0)	LMSP02010142
644.4472	GlcCer(d18:1/12:0)	LMSP0501AA01<br>HMDB0004969
650.2559	Cer(d18:0/24:1)	LMSP02020011
661.4330	CerPE 34:1;O2	LMSP03020020
664.5769	Cer(d18:1/25:0)	LMSP02010013
666.5900	Cer(d18:1/24:0(2OH))	LMSP02010161
672.4771	GlcCer(d18:1/14:0)	LMSP0501AA26
677.4322	SM(d18:0/14:0)	LMSP03010031<br>HMDB0012085
698.5223	HexCer 34:2;O2	LMSP05010088
701.4462	CerPE(d16:2/20:1(2OH))	LMSP03020090
708.4386	Cer(d18:0/28:0)	LMSP02020049
713.5047	CerPE(d14:2/24:1)	LMSP03020037
727.3919	SM(d18:2/18:1)	LMSP03010047
729.4986	SM(d18:1/18:1)	LMSP03010029
752.4773	GlcCer(d18:2/20:1)	LMSP0501AA35
757.4159	CerPE(d16:2/24:1(2OH))	LMSP03020094
776.5222	O-palmitoyl-Cer(d18:1/16:0)	LMSP02040003

**m/z**	**Sterol lipids (n=33)**	
227.1585	Porphobilinogen	HMDB0000245

271.1712	Estrone	LMST02010005
273.1608	Estradiol-17α	LMST02010029
275.1915	Alfa-androstenol	LMST02020008
275.2403	Androsterol	HMDB0303012
287.1414	Dehydrotestosteron	LMST02020018
289.1413	Testosterone	HMDB0000234
317.1863	Pregnenolone	LMST02030088
333.1338	21-hydroxypregnenolone	LMST02030167
347.2917	Corticosterone	LMST02030186
348.2567	Dihydrocorticosterone	LMST02030280
350.9629	Estrone 3-sulfate	LMST02010043
363.2873	Cortisol	LMST02030002 HMDB0000063
375.2135	Norcholestanol	LMST01010291
377.2006	Hydroxycortisone	LMST02030308
377.3121	Lithocholic acid	LMST04010004 HMDB0000717
391.2554	Ketolithocholic acid	LMST04010146 HMDB0000328
391.3150	12-Ketodeoxycholic acid	HMDB0000518
393.2387	Deoxycholic acid	LMST04010040
393.2828	3-Oxocholic acid	HMDB0000502
401.2646	5,6-trans-25-Hydroxyvitamin D2	HMDB0006721
405.2316	Cortisol 21-acetate	LMST02030093
407.3084	7-Ketodeoxycholic acid	LMST04010184 HMDB0000502
409.2355	Ursocholic acid	LMST04010088 HMDB0000917
419.2405	Dihydroxycholesterol	LMST04030178
421.2214	Homodeoxycholic acid	LMST04070031
437.2550	Dihomocholic acid	LMGP10050008
443.3031	Cortisol 21- sulfate	LMST05020020
465.3589	Testosterone glucuronide	LMST05010012
467.2805	Cholesterol sulfate	HMDB0000653
573.3914	Vitamin D2 3-glucuronide	LMST03010072
651.5252	18:1 Cholesterol ester	LMST01020003
653.3467	18:0 Cholesterol ester	LMST01020007

m/z	Oxylipins (n=11)	
297.2215	9-HODE	LMFA02000218
335.2043	PGA2	HMDB0001138

351.2261	PGE3	LMFA03010135 HMDB0002664
353.2089	Lipoxin A4	LMFA03040001 HMDB0060041
355.2534	PGF2a	LMFA03010025 HMDB0001139
357.2349	PGF1a	LMFA03010138 HMDB0002685
364.3155	HETE-Ethanolamine	LMFA08040039
369.2729	Epoxy PGE1	LMFA03020068
373.2079	Hydroxy-PGF1α	LMFA03010038
406.0417	12-HETE-GABA	LMFA08020147
406.3015	15-HETE-GABA	LMFA08020148

m/z	Lipid Antioxidants (n=5)	
**415.2636**	Ascorbyl palmitate	LMFA07010788
**425.2080**	Alpha-Tocotrienol	LMPR02020054
**537.4125**	β-carotene	LMPR01070001
**551.3144**	all-trans-retinyl oleate	LMFA07011033
**569.3515**	Lutein	LMPR01070031

m/z	Polar metabolites (n=51)	ID (HMDB/Lipidmaps)
123.0498	Erthrithol	HMDB0002994
125.9760	Taurine	HMDB0000251
134.9958	L-aspartic acid	HMDB0000191
143.0953	5-Hydroxymethyluracil	HMDB0000469
143.9933	Proline betaine	HMDB0004827
146.0266	Spermidine	HMDB0001257
148.0525	L-Glutamic acid	HMDB0000148
151.0863	D-Ribose	HMDB0000283
158.1423	Tiglylglycine	HMDB0000959
164.0681	Acetylcysteine	HMDB0001890
165.1019	Phenylpyruvic acid	HMDB0000205
167.0921	Methylxanthine	HMDB0001991
172.1510	Octylguanidine	HMDB0255917
172.1927	L-Homocysteine sulfate	HMDB0002238
180.0048	Glucosamine	HMDB0001514
180.1224	Hippuric acid	HMDB0000714
181.1081	Glucose	HMDB0003345
183.0683	Sorbitol	HMDB0000247

185.0252	Phosphorylcholine	HMDB0001565
186.2054	Phosphoserine	HMDB0000272
187.1071	2-Phosphoglyceric Acid	HMDB0000362
189.0742	L-Homoarginine	HMDB0000670
195.1191	D-Glucuronic Acid	HMDB0000127
200.2218	O-Phosphothreonine	HMDB0251566
202.2014	Cysteine-S-Sulfate	HMDB0000731
205.1166	Trytptophan	HMDB0000929
212.2219	Phosphocreatine	HMDB0001511
214.2373	Indoxyl Sulfate	HMDB0000682
222.1666	N-Acetyl-D-Glucosamine	HMDB0000215
228.2520	Deoxycytidine	HMDB0000014
235.1515	5-Methoxytryptophan	HMDB0002339
243.1196	Thymidine	HMDB0000274
245.0651	Uridine	HMDB0000296
250.0699	Cysteinyl-Glutamine	HMDB0028773
250.9820	Gamma-Glutamylcysteine	HMDB0001049
252.9801	Deoxyadenosine	HMDB0000101
279.1399	Leucyl-phenylalanine	HMDB0013243
326.9618	N-Oleoylethanolamine	LMFA08040015
328.9810	Phenylalanyltyrosine	HMDB0029007
340.3707	Oleoyl glycine	LMFA08020082
382.4177	N-Stearoyl Proline	LMFA08020119
389.2227	N-Linoleoyl Taurine	LMFA08020138
399.2791	N-Palmitoyltryptamine	HMDB0040815
408.3390	N-Linolenoyl Glutamic Acid	LMFA08020214
410.0358	N-Octadecadienoyl-Glutamic Acid	LMFA08020217
427.2586	N-Stearoyl Arginine	LMFA08020136
430.5908	N-Oleoyl Phenylalanine	LMFA08020092
432.3028	N-Stearoyl Phenylalanine	LMFA08020093
455.3069	N-Arachidonyltryptamine	HMDB0040817
463.2573	N-Arahidonyl Serotonin	LMFA08020141
469.2976	N-Oleoyl Tryptophan	LMFA08020096
