# Supplementary material for: Differential Metabolic Dysregulations in Hepatocellular Carcinoma and Cirrhosis: Insights into Lipidomic Signatures
Source: Biomolecules. 2025 Nov 10;15(11):1575. doi: 10.3390/biom15111575 (PMC12650657; doi:10.3390/biom15111575)
Supplement: Supplementary file 1 [file biomolecules-15-01575-s001.zip › Table S2. The VIP scores, Fold-change (FC), log2(FC), p-values and RF ranking of molecules.pdf]

**Table S2.** The VIP scores, Fold-change (FC), log2(FC), p-values (as determined by Volcano plot and T-test) and RF ranking of molecules from each of the 11 metabolite classes which may discriminate group CIR *vs* HCC.

| 1. **Fatty acids** | VIP SCORE | FC | log2FC | p-value | MDA | Relative variation |
|---|---|---|---|---|---|---|
| Docosapentenoic acid C22:5 | 1.279 | 3.217 | 1.686 | 3.32E-05 | 0.0258 | HCC<CIR |
| Palmitoleic acid C16:1 | 1.441 | 2.262 | 1.177 | 2.03E-06 | 0.0108 | HCC<CIR |
| Dodecenoic acid C12:1 | 1.380 | 2.219 | 1.150 | 6.11E-06 | <0.01 | HCC<CIR |
| Docosahexaenoic acid (DHA) C22:6 | 1.378 | 1.932 | 0.950 | 6.31E-06 | 0.0147 | HCC<CIR |
| 3-Hydroxysuberic acid C8:1;O3 | 1.403 | 1.671 | 0.741 | 4.05E-06 | 0.0080 | HCC<CIR |
| Triacontatrienoic acid C30:3 | 1.512 | 1.652 | 0.724 | 4.99E-07 | 0.0137 | HCC<CIR |
| Methyl-tridecanedioic acid C14:2 | 1.352 | 0.675 | -0.566 | 9.97E-06 | <0.01 | HCC>CIR |
| Octatriacontanoic acid C 38:0 | 1.636 | 0.603 | -0.731 | 3.24E-08 | <0.01 | HCC>CIR |
| Hydroxy-Eicosapentenoic acid C20:5;O | 1.808 | 0.562 | -0.832 | 3.66E-10 | 0.0168 | HCC>CIR |
| Tetracontahexaenoic acid C40:6 | 1.714 | 0.457 | -1.130 | 4.75E-09 | 0.0281 | HCC>CIR |
| Stearic acid C18:0 | 1.286 | 0.392 | -1.351 | 2.99E-05 | <0.01 | HCC>CIR |
| 10-oxo-docosanoic acid C22:1;O | 1.503 | 0.342 | -1.548 | 5.99E-07 | 0.0081 | HCC>CIR |
| Arachidic acid C20:0 | 1.773 | 0.135 | -2.893 | 1.00E-09 | 0.0188 | HCC>CIR |
| Heptadecenoic acid C17:1 | 1.786 | 0.122 | -3.039 | 6.85E-10 | 0.0436 | HCC>CIR |
| Triacontatetraenoic acid CC30:4 | 1.557 | 0.087 | -3.531 | 1.95E-07 | <0.01 | HCC>CIR |

| 1. **FA derivatives** | VIP SCORE | FC | log2(FC) | p-value | MDA | Relative variation |
|---|---|---|---|---|---|---|
| Palmitoleyl palmitoleate | 0.722 | 2.372 | 1.246 | 4.58E-02 | 0.0020 | HCC<CIR |
| Amino-octanoic acid | 1.307 | 2.285 | 1.192 | 1.94E-04 | 0.0115 | HCC<CIR |
| Stearamide | 1.476 | 1.610 | 0.687 | 2.00E-05 | 0.0422 | HCC<CIR |
| Myristoleyl arachidonate | 0.482 | 1.441 | 0.527 | 1.86E-01 | 0.0021 | HCC<CIR |
| Docosenamide | 0.595 | 1.202 | 0.266 | 1.01E-01 | 0.0260 | HCC<CIR |
| Palmityl palmitoleate | 0.317 | 1.140 | 0.189 | 3.86E-01 | 0.0483 | HCC<CIR |
| Linolenyl stearate | 0.375 | 0.889 | -0.169 | 3.05E-01 | 0.0085 | HCC>CIR |
| Stearyl stearate | 0.546 | 0.885 | -0.175 | 1.33E-01 | 0.0074 | HCC>CIR |
| Oleyl palmitate | 0.539 | 0.848 | -0.238 | 1.39E-01 | 0.0032 | HCC>CIR |
| Linoleyl arachidonate | 0.621 | 0.838 | -0.254 | 8.69E-02 | 0.0082 | HCC>CIR |
| Linoleyl stearate | 0.449 | 0.836 | -0.258 | 2.18E-01 | 0.0058 | HCC>CIR |
| Linoleyl linoleate | 0.832 | 0.711 | -0.492 | 2.08E-02 | 0.0045 | HCC>CIR |
| Palmitoleyl linolenate | 1.598 | 0.672 | -0.574 | 3.01E-06 | 0.0483 | HCC>CIR |
| Linoleyl arachidate | 1.800 | 0.558 | -0.842 | 7.79E-08 | 0.0878 | HCC>CIR |
| Palmitamide | 1.761 | 0.313 | -1.677 | 1.69E-07 | 0.0618 | HCC>CIR |

| 2. **Glycerophospholipids** | VIP SCORE | FC | log2(FC) | p-value | MDA | Relative variation |
|---|---|---|---|---|---|---|
| Glycerophosphocholine | 1.134 | 2.599 | 1.378 | 3.03E-04 | 0.0119 | HCC<CIR |
| PC (23:2; O) | 1.794 | 2.408 | 1.268 | 7.02E-10 | 0.0297 | HCC<CIR |
| PA 30:2 | 1.503 | 1.709 | 0.773 | 6.95E-07 | 0.0305 | HCC<CIR |
| PA (30:4;O3) | 0.983 | 1.600 | 0.678 | 1.94E-03 | 0.0109 | HCC<CIR |

| | | | | | | |
|---|---|---|---|---|---|---|
| PA(O-18:0/16:0) | 1.249 | 1.469 | 0.555 | 5.87E-05 | <0.006 | HCC<CIR |
| PA 32:0 | 1.161 | 0.715 | -0.483 | 2.08E-04 | <0.006 | HCC>CIR |
| PA (O-36:3) | 1.005 | 0.698 | -0.518 | 1.50E-03 | <0.006 | HCC>CIR |
| PC 32:1 | 1.124 | 0.683 | -0.550 | 3.46E-04 | 0.0082 | HCC>CIR |
| PA 36:6 | 1.488 | 0.592 | -0.755 | 9.30E-07 | 0.0179 | HCC>CIR |
| PA(P-18:0/18:2) | 1.131 | 0.557 | -0.845 | 3.12E-04 | 0.0065 | HCC>CIR |
| PA 38:6 | 1.411 | 0.531 | -0.912 | 3.96E-06 | 0.0138 | HCC>CIR |
| PS 34:0 | 1.534 | 0.514 | -0.959 | 3.70E-07 | 0.0237 | HCC>CIR |
| PG O-34:4 | 1.307 | 0.441 | -1.181 | 2.35E-05 | 0.0111 | HCC>CIR |
| PA 42:4 | 1.609 | 0.427 | -1.227 | 7.41E-08 | 0.0252 | HCC>CIR |
| PE (O-38:4) | 1.241 | 0.249 | -2.005 | 6.56E-05 | 0.0085 | HCC>CIR |

| **3. Lysophospholipids** | VIP SCORE | FC | log2(FC) | p-value | MDA | Relative variation |
|---|---|---|---|---|---|---|
| LysoPE (16:1) | 1.343 | 15.412 | 3.946 | 1.28E-03 | 0.0034 | HCC<CIR |
| LysoPS (16:1) | 1.121 | 15.412 | 3.946 | 7.81E-03 | 0.0034 | HCC<CIR |
| LysoPC (19:3) | 2.094 | 2.227 | 1.155 | 1.15E-07 | 0.0374 | HCC<CIR |
| LysoPE (22:6) | 1.491 | 1.503 | 0.588 | 3.13E-04 | 0.0119 | HCC<CIR |
| LysoPE (18:0) | 0.950 | 1.264 | 0.338 | 2.50E-02 | 0.0040 | HCC<CIR |
| LysoPC (18:1) | 0.848 | 1.248 | 0.320 | 4.62E-02 | <0.003 | HCC<CIR |
| LysoPC(16:0) | 0.825 | 0.917 | -0.125 | 5.26E-02 | <0.003 | HCC>CIR |
| LysoPI (18:3) | 1.108 | 0.849 | -0.236 | 8.55E-03 | 0.0301 | HCC>CIR |
| LysoPA (P-16:0) | 1.160 | 0.777 | -0.363 | 5.81E-03 | <0.003 | HCC>CIR |
| LysoPI (18:2) | 0.870 | 0.640 | -0.645 | 4.08E-02 | 0.0181 | HCC>CIR |
| LysoPC(20:3) | 1.635 | 0.585 | -0.774 | 6.67E-05 | 0.0450 | HCC>CIR |
| LysoPC (22:1) | 1.045 | 0.483 | -1.050 | 1.34E-02 | <0.003 | HCC>CIR |
| LysoPC(22:6) | 1.643 | 0.447 | -1.161 | 6.02E-05 | 0.0186 | HCC>CIR |
| LysoPA (18:1) | 1.557 | 0.415 | -1.269 | 1.57E-04 | 0.0060 | HCC>CIR |
| LysoPA (20:3) | 1.541 | 0.362 | -1.465 | 1.87E-04 | 0.0066 | HCC>CIR |

| **5. Acylcarnitines** | VIP SCORE | FC | log2(FC) | p-value | MDA | Relative variation |
|---|---|---|---|---|---|---|
| Hexadecadienoylcarnitine C16:2 | 1.464 | 2.862 | 1.517 | 6.98E-05 | 0.0205 | HCC<CIR |
| Tetradecanoylcarnitine CAR 14:0 | 1.278 | 1.573 | 0.654 | 6.08E-04 | 0.0367 | HCC<CIR |
| Palmitoleoylcarnitine CAR 16:1 | 1.271 | 1.567 | 0.648 | 6.50E-04 | 0.0199 | HCC<CIR |
| Hexacosanoyl carnitine CAR 26:0 | 1.175 | 0.670 | -0.578 | 1.57E-02 | 0.0157 | HCC>CIR |
| Hydroxylauroylcarnitine CAR 12:0;O | 1.241 | 0.581 | -0.783 | 9.02E-04 | 0.0242 | HCC>CIR |
| Hydroxypalmitoleoylcarnitine CAR 16:1;O | 0.971 | 0.471 | -1.087 | 1.04E-02 | <0.01 | HCC>CIR |
| Carboxyheptadecanoyl)carnitine C18:1;O2 | 1.513 | 0.342 | -1.547 | 3.71E-05 | 0.0389 | HCC>CIR |
| Hydroxydodecenoylcarnitine CAR 12:1;O | 1.385 | 0.306 | -1.707 | 1.83E-04 | 0.0122 | HCC>CIR |
| Dodecadienoylcarnitine CAR 12:2 | 1.237 | 0.277 | -1.851 | 9.41E-04 | 0.0133 | HCC>CIR |
| Arachidyl carnitine CAR 20:0 | 1.663 | 0.263 | -1.928 | 4.41E-06 | 0.0499 | HCC>CIR |
| Octenoylcarnitine CAR 8:1 | 1.260 | 0.201 | -2.312 | 7.36E-04 | 0.0242 | HCC>CIR |

| 6. Mono- and diglycerides | VIP SCORE | FC | log2(FC) | P-value | MDA | Relative variation |
|---|---|---|---|---|---|---|
| DG (44:0) | 0.934 | 2.149 | 1.103 | 4.64E-03 | 0.0865 | HCC<CIR |
| MG(20:4) | 1.486 | 1.978 | 0.984 | 2.57E-06 | 0.0194 | HCC<CIR |
| DG(35:1) | 1.635 | 1.745 | 0.803 | 1.43E-07 | 0.0433 | HCC<CIR |
| DG(33:4) | 1.826 | 1.603 | 0.680 | 1.61E-09 | 0.0346 | HCC<CIR |
| DG(33:3) | 0.874 | 1.373 | 0.457 | 8.33E-03 | 0.0025 | HCC<CIR |
| DG(34:1) | 0.813 | 1.164 | 0.219 | 1.43E-02 | 0.0049 | HCC<CIR |
| DG(34:4) | 1.284 | 0.804 | -0.315 | 6.64E-05 | 0.0049 | HCC>CIR |
| DG(42:0) | 1.163 | 0.539 | -0.891 | 3.48E-04 | 0.0159 | HCC>CIR |
| DG 40:7 | 1.139 | 0.507 | -0.981 | 4.69E-04 | 0.0413 | HCC>CIR |
| MGDG (34:3) | 1.548 | 0.436 | -1.196 | 8.18E-07 | 0.0225 | HCC>CIR |
| MGMG (16:2) | 1.235 | 0.373 | -1.424 | 1.33E-04 | 0.0043 | HCC>CIR |

| 7. Sphingolipids | VIP SCORE | FC | log2(FC) | p-value | MDA | Relative variation |
|---|---|---|---|---|---|---|
| CerPE(d14:2/16:0(2OH)) | 1.707 | 3.121 | 1.642 | 9.56E-10 | 0.0365 | HCC<CIR |
| Cer(d18:2/20:1) | 1.094 | 2.511 | 1.329 | 2.67E-04 | 0.0005 | HCC<CIR |
| C19 Sphingosine-1-phosphate | 1.570 | 2.278 | 1.188 | 3.61E-08 | 0.0233 | HCC<CIR |
| Cer(t18:1(6OH)/16:0(2OH)) | 1.589 | 1.964 | 0.974 | 2.25E-08 | 0.0365 | HCC<CIR |
| Cer(t18:0/19:0(2OH)) | 1.295 | 1.802 | 0.850 | 1.11E-05 | 0.0365 | HCC<CIR |
| Sphingosine 18:2; O2 | 1.906 | 1.706 | 0.771 | 1.38E-12 | 0.0929 | HCC<CIR |
| Cer(t18:0/20:0(2OH)) | 1.159 | 0.770 | -0.377 | 1.03E-04 | 0.0066 | HCC>CIR |
| GlcCer(d18:1/14:0) | 1.125 | 0.695 | -0.524 | 1.72E-04 | 0.0046 | HCC>CIR |
| CerPE(d16:1/16:0) | 1.205 | 0.665 | -0.588 | 5.01E-05 | 0.0066 | HCC>CIR |
| SM(d18:0/14:0) | 1.455 | 0.588 | -0.765 | 4.97E-07 | 0.0135 | HCC>CIR |
| CerPE(d16:2/24:1(2OH)) | 1.742 | 0.586 | -0.771 | 3.44E-10 | 0.0518 | HCC>CIR |
| SM(d18:1/18:1) | 1.421 | 0.500 | -0.999 | 9.94E-07 | 0.0181 | HCC>CIR |
| CerPE(d16:2/20:1(2OH)) | 1.386 | 0.474 | -1.079 | 2.03E-06 | 0.0066 | HCC>CIR |
| Cer(d18:2/18:1) | 1.118 | 0.459 | -1.123 | 1.90E-04 | 0.0011 | HCC>CIR |
| Cer(d18:0/24:1) | 0.938 | 0.459 | -1.123 | 2.00E-03 | 0.0005 | HCC>CIR |

| 8. Sterol lipids | VIP SCORE | FC | log2(FC) | p-value | MDA | Relative variation |
|---|---|---|---|---|---|---|
| Norcholestanol | 0.744 | 2.730 | 1.449 | 6.28E-02 | 0.0008 | HCC<CIR |
| 18:0 Cholesterol ester | 1.425 | 1.749 | 0.807 | 2.34E-04 | 0.0008 | HCC<CIR |
| Dihydrocorticosterone | 1.756 | 1.662 | 0.733 | 3.38E-06 | 0.0458 | HCC<CIR |
| Cortisol | 1.820 | 1.652 | 0.724 | 1.28E-06 | 0.0667 | HCC<CIR |
| Estrone 3-sulfate | 1.435 | 1.576 | 0.656 | 2.10E-04 | 0.0193 | HCC<CIR |
| Alfa-androstenol | 1.216 | 1.355 | 0.438 | 1.93E-03 | 0.0286 | HCC<CIR |
| Pregnenolone | 0.996 | 1.332 | 0.414 | 1.19E-02 | 0.0386 | HCC<CIR |
| Cortisol 21-acetate | 1.027 | 1.193 | 0.254 | 9.48E-03 | 0.0121 | HCC<CIR |
| Dihomocholic acid | 0.909 | 0.775 | -0.368 | 5.55E-03 | 0.0055 | HCC>CIR |
| 21-hydroxypregnenolone | 1.603 | 0.724 | -0.466 | 2.81E-05 | 0.0095 | HCC>CIR |
| Cholesterol sulfate | 0.917 | 0.701 | -0.513 | 2.10E-02 | 0.0007 | HCC>CIR |

| | | | | | | |
|---|---|---|---|---|---|---|
| Deoxycholic acid | 0.783 | 0.679 | -0.559 | 7.00E-03 | 0.0070 | HCC>CIR |
| 3-Oxocholic acid | 1.546 | 0.603 | -0.731 | 5.74E-05 | 0.0172 | HCC>CIR |
| 18:1 Cholesterol ester | 1.575 | 0.199 | -2.332 | 4.00E-05 | 0.0409 | HCC>CIR |
| 25-Hydroxyvitamin D2 | 1.914 | 0.152 | -2.719 | 2.76E-07 | 0.0293 | HCC>CIR |

| 9. **Oxilipins** | Comp. 1 | FC | log2(FC) | p-value | MDA | Relative variation |
|---|---|---|---|---|---|---|
| HETE-Ethanolamine | 1.437 | 2.387 | 1.255 | 8.67E-06 | 0.0143 | HCC<CIR |
| Hydroxy-PGF1a | 0.834 | 0.634 | -0.657 | 0.001084 | 0.0127 | HCC>CIR |
| Epoxy PGE1 | 1.607 | 0.607 | -0.721 | 8.24E-08 | 0.1438 | HCC>CIR |
| PGF1a | 1.802 | 0.151 | -2.726 | 5.87E-09 | 0.1337 | HCC>CIR |

| **10. Antioxidants** | VIP SCORE | FC | log2(FC) | p-value | MDA | Relative variation |
|---|---|---|---|---|---|---|
| Alpha-Tocotrienol | 1.048 | 2.377 | 1.249 | 0.0845 | 0.0477 | HCC<CIR |
| all-trans-retinyl oleate | 1.059 | 1.557 | 0.639 | 0.0811 | 0.0180 | HCC<CIR |
| b-carotene | 0.550 | 0.626 | -0.677 | 0.3684 | 0.0136 | HCC>CIR |
| Ascorbyl palmitate | 1.216 | 0.505 | -0.987 | 0.0445 | 0.0838 | HCC>CIR |

| **11.Polar molecules** | VIP SCORE | FC | log2(FC) | p-value | MDA | Relative variation |
|---|---|---|---|---|---|---|
| Glucose | 1.665 | 1.638 | 0.711 | 6.03E-09 | 0.0132 | HCC<CIR |
| Phenylalanyltyrosine | 1.237 | 1.584 | 0.663 | 2.74E-05 | <0.007 | HCC<CIR |
| 5-Hydroxymethyluracil | 1.716 | 1.400 | 0.485 | 1.54E-09 | 0.0230 | HCC<CIR |
| Hippuric acid | 1.357 | 0.829 | -0.270 | 4.07E-05 | 0.0352 | HCC>CIR |
| N-Acetyl-D-glucosamine | 1.343 | 0.691 | -0.533 | 5.63E-06 | <0.007 | HCC>CIR |
| Spermidine | 1.261 | 0.654 | -0.612 | 6.09E-08 | 0.0204 | HCC>CIR |
| Taurine | 1.576 | 0.630 | -0.667 | 5.24E-06 | <0.007 | HCC>CIR |
| O-Phosphothreonine | 1.347 | 0.553 | -0.855 | 6.23E-06 | <0.007 | HCC>CIR |
| Phosphoserine | 1.543 | 0.547 | -0.870 | 1.64E-05 | 0.0374 | HCC>CIR |
| Proline betaine | 1.572 | 0.544 | -0.878 | 6.70E-06 | 0.0199 | HCC>CIR |
| N-stearoyl phenylalanine | 1.237 | 0.457 | -1.129 | 5.46E-08 | 0.0110 | HCC>CIR |
| N-Oleoylethanolamine | 1.565 | 0.453 | -1.144 | 7.20E-08 | 0.0199 | HCC>CIR |
| N-Palmitoyltryptamine | 1.292 | 0.453 | -1.144 | 1.18E-07 | 0.0096 | HCC>CIR |
| Phosphocreatine | 1.353 | 0.429 | -1.221 | 6.34E-06 | 0.0073 | HCC>CIR |
| Oleoyl glycine | 1.346 | 0.255 | -1.969 | 4.03E-05 | <0.007 | HCC>CIR |
