# Supplementary material for: Differential Metabolic Dysregulations in Hepatocellular Carcinoma and Cirrhosis: Insights into Lipidomic Signatures
Source: Biomolecules. 2025 Nov 10;15(11):1575. doi: 10.3390/biom15111575 (PMC12650657; doi:10.3390/biom15111575)
Supplement: Supplementary file 1 [file biomolecules-15-01575-s001.zip › Table S3. Biomarker analysis. AUC values for each class of metabolites.pdf]

Table S3. Results of Biomarker analysis considering each class of metabolites.

**Free fatty acids**	**AUC**	**T-tests**	**Log2 FC**	**Relative variation**
Heptadecenoic acid C17:1	0.934	6.85E-10	-2.218	HCC>CIR
Octatriacontanoic acid C 38:0	0.894	3.24E-08	-0.119	HCC>CIR
Tetracontahexaenoic acid C40:6	0.874	4.75E-09	-0.483	HCC>CIR
Hydroxy-Eicosapentenoic acid C20:5;O	0.864	3.66E-10	-0.181	HCC>CIR
Docosapentenoic acid C22:5	0.855	3.32E-05	2.203	HCC<CIR
Arachidic acid C20:0	0.852	1.00E-09	-2.039	HCC>CIR
Triacontatrienoic acid C30:3	0.842	4.99E-07	1.224	HCC<CIR
Methyl-tridecanedioic acid C14:2	0.810	9.97E-06	0.018	HCC<CIR
10-oxo-docosanoic acid C22:1;O	0.784	5.99E-07	-0.781	HCC>CIR
Docosatrienoic acid C22:3	0.784	4.70E-05	-0.092	HCC>CIR
Stearic acid C18:0	0.780	2.99E-05	-0.686	HCC>CIR
Myristic acid C14:0	0.776	1.24E-04	0.085	HCC<CIR
3-Hydroxysuberic acid C8:1;O3	0.771	4.05E-06	1.092	HCC<CIR
FA 30:2	0.767	3.47E-03	-0.210	HCC>CIR
Palmitoleic acid C16:1	0.763	2.03E-06	1.720	HCC<CIR
Dodecenoic acid C12:1	0.762	6.11E-06	1.791	HCC<CIR
Tetracosanoic (lignoceric) acid C24:0	0.760	3.02E-03	0.146	HCC<CIR
Docosahexaenoic acid (DHA) C22:6	0.753	6.31E-06	1.296	HCC<CIR
Triacontatetraenoic acid C 30:3	0.747	1.95E-07	-2.702	HCC>CIR
Triacontadienoic acid C30:2	0.742	9.88E-03	-0.166	HCC>CIR

**Fatty acid derivatives**	**AUC**	**T-tests**	**Log2 FC**	**Relative variation**
Linoleyl arachidate	0.810	7.79E-08	-0.785	HCC>CIR
Stearamide	0.801	2.00E-05	0.515	HCC<CIR
Palmitoleyl linolenate	0.785	3.01E-06	-0.583	HCC>CIR
Docosenamide	0.737	1.01E-01	0.243	HCC<CIR
Palmitoleyl palmitoleate	0.721	4.58E-02	0.860	HCC<CIR
Amino-octanoic acid	0.696	1.94E-04	1.019	HCC<CIR
Palmitamide	0.675	1.69E-07	-1.674	HCC>CIR
Linoleyl linoleate	0.647	2.08E-02	-0.460	HCC>CIR
Linoleyl arachidonate	0.631	8.69E-02	-0.356	HCC>CIR
Stearyl stearate	0.627	1.33E-01	-0.267	HCC>CIR
Oleyl palmitate	0.615	1.39E-01	-0.227	HCC>CIR
Myristyl palmitate	0.607	8.72E-01	0.039	HCC<CIR
Myristoleyl arachidonate	0.588	1.86E-01	0.320	HCC<CIR
Linoleyl stearate	0.547	2.18E-01	-0.327	HCC>CIR
Palmityl palmitoleate	0.538	3.86E-01	0.093	HCC<CIR
Linolenyl stearate	0.503	3.05E-01	-0.083	HCC>CIR

**Glycerophospholipids**	**AUC**	**T-tests**	**Log2 FC**	**Relative variation**

PC (23:2; O)	0.877	7.02E-10	0.661	HCC<CIR
PA 30:2	0.852	6.95E-07	0.273	HCC<CIR
PA 42:4	0.828	7.41E-08	-1.585	HCC>CIR
PS 34:0	0.804	3.70E-07	-1.340	HCC>CIR
PA(O-18:0/16:0)	0.769	5.87E-05	0.091	HCC<CIR
PA 38:6	0.767	3.96E-06	-1.381	HCC>CIR
Glycerophosphocholine	0.766	3.03E-04	0.659	HCC<CIR
PA 23:0	0.750	1.87E-02	0.473	HCC<CIR
PE 30:3	0.750	3.51E-03	0.308	HCC<CIR
PG O-34:4	0.736	2.35E-05	-1.523	HCC>CIR
PA 36:6	0.735	9.30E-07	-1.132	HCC>CIR
PA (30:4;O3)	0.734	1.94E-03	0.129	HCC<CIR
PC 32:1	0.729	3.46E-04	-0.935	HCC>CIR
PA 32:5	0.709	2.73E-03	-0.086	HCC>CIR
PA(P-18:0/18:2)	0.688	3.12E-04	-1.244	HCC>CIR
PA (O-36:3)	0.668	1.50E-03	-0.935	HCC>CIR
PG 16:0	0.662	3.57E-01	-0.208	HCC>CIR
PA 36:0	0.659	3.16E-03	-1.144	HCC>CIR
PC(P-16:0/16:1))	0.649	4.81E-03	-0.838	HCC>CIR
PA 32:0	0.648	2.08E-04	-0.896	HCC>CIR

**Lysophospholipids**	**AUC**	**T-tests**	**Log2 FC**	**Relative variation**
LysoPC(20:3)	0.793	6.67E-05	-0.769	HCC>CIR
LysoPC (19:3)	0.769	1.15E-07	0.962	HCC<CIR
LysoPC (22:1)	0.749	1.34E-02	-1.021	HCC>CIR
LysoPC(22:6)	0.737	6.02E-05	-1.122	HCC>CIR
LysoPI (18:3)	0.735	8.55E-03	-0.301	HCC>CIR
LysoPE (22:6)	0.727	3.13E-04	0.391	HCC<CIR
LysoPE (18:0)	0.672	2.50E-02	0.270	HCC<CIR
LysoPA (18:1)	0.657	1.57E-04	-1.287	HCC>CIR
LysoPE (16:1)	0.644	1.28E-03	0.421	HCC<CIR
LysoPA (P-16:0)	0.643	5.81E-03	-0.326	HCC>CIR
LysoPA (20:3)	0.640	1.87E-04	-1.392	HCC>CIR
LysoPA (18:3)	0.626	1.22E-01	0.249	HCC<CIR
LysoPA (22:2)	0.624	3.06E-01	0.221	HCC<CIR
LysoPA (22:1)	0.605	3.78E-01	-0.119	HCC>CIR
LysoPI (18:2)	0.605	4.08E-02	-0.753	HCC>CIR
LysoPC(16:0)	0.602	5.26E-02	-0.164	HCC>CIR
LysoPG (18:1)	0.595	5.86E-01	-0.043	HCC>CIR
LysoPC(16:1)	0.592	2.64E-01	0.157	HCC<CIR
LysoPA (14:0)	0.588	1.50E-01	0.150	HCC<CIR
LysoPC (18:1)	0.586	4.62E-02	0.165	HCC<CIR

AcylCarnitines	AUC	T-tests	Log2 FC	Relative variation
Hexadecadienoylcarnitine CAR16:2	0.777	6.98E-05	1.226	HCC<CIR
Tetradecanoylcarnitine CAR 14:0	0.726	6.08E-04	0.597	HCC<CIR
Carboxyheptadecanoyl)carnitine C18:1;O2	0.705	3.71E-05	-1.267	HCC>CIR
Palmitoleoylcarnitine CAR 16:1	0.702	6.56E-04	0.585	HCC<CIR
Arachidyl carnitine CAR 20:0	0.691	4.41E-06	-1.545	HCC>CIR
Hydroxytetradecadienoylcarnitine CAR 14:2;O	0.673	2.19E-03	0.672	HCC<CIR
Dodecadienoylcarnitine CAR 12:2	0.649	9.41E-04	-1.435	HCC>CIR
Hexacosanoyl carnitine CAR 26:0	0.634	1.74E-03	-0.327	HCC>CIR
Hydroxylauroylcarnitine CAR 12:0;O	0.626	9.02E-04	-0.551	HCC>CIR
Hydroxydodecenoylcarnitine CAR 12:1;O	0.622	1.83E-04	-1.115	HCC>CIR
Palmitoylcarnitine C16:0	0.601	8.70E-02	0.299	HCC<CIR
Hydroxypalmitoleoylcarnitine CAR 16:1;O	0.592	1.04E-02	-0.603	HCC>CIR
Dodecenoylcarnitine CAR 12:1	0.579	1.08E-01	-0.429	HCC>CIR
Hydroxyoctadecatrienoylcarnitine CAR 18:3;O	0.571	4.68E-01	0.170	HCC<CIR
Hydroxymyristoylcarnitine C14:0	0.560	2.77E-01	-0.040	HCC>CIR
Linoleylcarnitine CAR 18:2	0.559	1.09E-01	-0.085	HCC>CIR
Octenoylcarnitine CAR 8:1	0.549	7.36E-04	-1.843	HCC>CIR
Carboxytridecanoyl carnitine CAR 14:1;O2	0.526	5.44E-02	-0.377	HCC>CIR
Hydroxyoctadecenoylcarnitine CAR 18:1;O	0.524	4.06E-01	0.255	HCC<CIR
Butenylcarnitine CAR 4:1	0.516	8.09E-01	0.149	HCC<CIR

Mono- and Diglycerides	AUC	T-tests	Log2 FC	Relative variation
DG(33:4)	0.830	1.61E-09	0.209	HCC<CIR
DG 40:7	0.829	4.69E-04	-1.426	HCC>CIR
DG(35:1)	0.818	1.43E-07	0.296	HCC<CIR
MG(20:4)	0.813	2.57E-06	0.503	HCC<CIR
DG (44:0)	0.794	4.64E-03	0.715	HCC<CIR
MGDG (34:3)	0.781	8.18E-07	-1.804	HCC>CIR
DG (40:1)	0.757	2.98E-01	0.222	HCC<CIR
DG(34:4)	0.739	6.64E-05	-0.852	HCC>CIR
DG(42:0)	0.723	3.48E-04	-1.524	HCC>CIR
DG(34:1)	0.708	1.43E-02	-0.301	HCC>CIR
MGMG (16:2)	0.688	1.33E-04	-1.976	HCC>CIR
DG(33:3)	0.682	8.33E-03	-0.045	HCC>CIR
DG 40:6	0.666	3.88E-02	-0.879	HCC>CIR
DG (44:12)	0.642	1.48E-01	-0.671	HCC>CIR
DG 40:4	0.616	1.78E-01	-0.242	HCC>CIR
DG 36:4	0.598	2.04E-01	-0.866	HCC>CIR
DG 36:0	0.543	8.19E-01	-0.421	HCC>CIR
DG(33:1)	0.526	7.32E-01	-0.483	HCC>CIR
MGMG(18:2)	0.525	7.16E-01	-0.537	HCC>CIR
DG(34:0)	0.518	7.65E-01	-0.535	HCC>CIR

Sphingolipids	AUC	T-tests	Log2 FC	Relative variation
Sphingosine 18:2; O2	0.911	1.38E-12	0.443	HCC<CIR
CerPE(d14:2/16:0(2OH))	0.872	9.56E-10	1.110	HCC<CIR
CerPE(d16:2/24:1(2OH))	0.857	3.44E-10	-1.014	HCC>CIR
Cer(t18:1(6OH)/16:0(2OH))	0.840	2.25E-08	0.648	HCC<CIR
C19 Sphingosine-1-phosphate	0.833	3.61E-08	0.793	HCC<CIR
SM(d18:1/18:1)	0.795	9.94E-07	-1.230	HCC>CIR
SM(d18:0/14:0)	0.791	4.97E-07	-1.047	HCC>CIR
CerPE(d16:2/20:1(2OH))	0.785	2.03E-06	-1.368	HCC>CIR
Cer(t18:0/19:0(2OH))	0.775	1.11E-05	0.556	HCC<CIR
Cer(d18:2/20:1)	0.751	2.67E-04	1.026	HCC<CIR
GlcCer(d18:1/14:0)	0.731	1.72E-04	-0.801	HCC>CIR
CerPE(d16:1/16:0)	0.718	5.01E-05	-0.856	HCC>CIR
Cer(t18:0/20:0(2OH))	0.710	1.03E-04	-0.636	HCC>CIR
C18 Sphingosine-1-phosphate	0.673	2.08E-03	0.033	HCC<CIR
Cer(t18:1(6OH)/20:0)	0.667	1.77E-02	-0.024	HCC>CIR
GlcCer(d18:1/12:0)	0.666	7.48E-03	-0.502	HCC>CIR

Sterol lipids	AUC	T-tests	Log2 FC	Relative variation
25-Hydroxyvitamin D2	0.771	2.76E-07	-2.857	HCC>CIR
Cortisol	0.768	1.28E-06	0.584	HCC<CIR
3-Oxocholic acid	0.766	5.74E-05	-0.868	HCC>CIR
21-hydroxypregnenolone	0.762	2.81E-05	-0.562	HCC>CIR
Alfa-androstenol	0.727	1.93E-03	0.311	HCC<CIR
Dihydrocorticosterone	0.709	3.38E-06	0.689	HCC<CIR
Dihydroxycholesterol	0.694	3.16E-01	0.614	HCC<CIR
18:0 Cholesterol ester	0.678	2.34E-04	0.776	HCC<CIR
Dihomocholic acid	0.676	2.23E-02	-0.398	HCC>CIR
Corticosterone	0.657	1.52E-01	0.115	HCC<CIR
Estrone 3-sulfate	0.647	2.10E-04	0.626	HCC<CIR
12-Ketodeoxycholic acid	0.642	8.59E-02	0.118	HCC<CIR
Deoxycholic acid	0.637	5.00E-02	-0.592	HCC>CIR
7-Ketodeoxycholic acid	0.625	3.38E-01	-0.001	HCC>CIR
Estradiol-17?	0.621	1.59E-01	0.209	HCC<CIR
Cholesterol sulfate	0.602	2.10E-02	-0.555	HCC>CIR
Cortisol 21-acetate	0.595	9.48E-03	0.205	HCC<CIR
Ketolithocholic acid	0.593	2.29E-01	0.117	HCC<CIR
Hydroxycortisone	0.588	2.17E-01	0.236	HCC<CIR
Cortisol 21- sulfate	0.580	3.27E-01	0.040	HCC<CIR

Oxilipins	AUC	T-tests	Log2 FC	Relative variation
PGF1a	0.894	1.18E-08	-2.240	HCC>CIR
Epoxy PGE1	0.840	8.24E-08	-0.288	HCC>CIR
HETE-Ethanolamine	0.738	6.43E-06	1.234	HCC<CIR
Hydroxy-PGF1a	0.722	1.08E-03	-0.246	HCC>CIR
15-HETE-GABA	0.703	9.23E-03	-0.068	HCC>CIR
PGE3	0.657	1.05E-02	0.065	HCC<CIR
PGA2	0.614	8.78E-01	0.221	HCC<CIR
9-HODE	0.592	2.39E-01	0.556	HCC<CIR
Lipoxin A4	0.583	7.76E-01	0.286	HCC<CIR
PGF2b	0.524	8.94E-02	0.719	HCC<CIR

Antioxidants	AUC	T-tests	Log2 FC	Relative variation
Ascorbyl palmitate	0.729	0.045	-0.644	HCC>CIR
Alpha-Tocotrienol	0.594	0.084	0.588	HCC<CIR
all-trans-retinyl oleate	0.584	0.081	-0.002	HCC>CIR
beta-carotene	0.502	0.368	-0.173	HCC>CIR

Polar metabolites	AUC	T-tests	Log2 FC	Relative variation
N-Acetyl-D-glucosamine	0.870	6.70E-06	0.064	HCC<CIR
N-Palmitoyltryptamine	0.860	1.64E-05	0.036	HCC<CIR
N-Oleoylethanolamine	0.859	7.20E-08	-0.455	HCC>CIR
Glucose	0.856	6.03E-09	1.127	HCC<CIR
5-Hydroxymethyluracil	0.851	1.54E-09	0.946	HCC<CIR
Proline betaine	0.851	6.09E-08	-0.219	HCC>CIR
Phosphoserine	0.844	1.18E-07	-0.199	HCC>CIR
Taurine	0.835	5.46E-08	0.014	HCC<CIR
L-Homocysteine sulfate	0.826	1.53E-01	0.743	HCC<CIR
N-stearoyl phenylalanine	0.800	4.07E-05	-0.479	HCC>CIR
Trytptophan	0.790	5.44E-03	-0.237	HCC>CIR
N-linolenoyl glutamic acid	0.788	8.54E-04	0.194	HCC<CIR
Hippuric acid	0.779	5.24E-06	0.341	HCC<CIR
Cysteine-S-sulfate	0.770	1.79E-03	0.821	HCC<CIR
Spermidine	0.769	2.74E-05	0.038	HCC<CIR
O-Phosphothreonine	0.763	6.23E-06	-0.142	HCC>CIR
Leucyl-phenylalanine	0.759	8.55E-04	0.207	HCC<CIR
Oleoyl glycine	0.748	6.34E-06	-1.146	HCC>CIR
Deoxycytidine	0.715	1.03E-04	-1.078	HCC>CIR
